# Synthesis and characterization of new hexahydroquinoline derivatives and evaluation of their cytotoxicity, intracellular ROS production, and inhibitory effects on inflammatory mediators

**DOI:** 10.55730/1300-0527.3686

**Published:** 2024-07-23

**Authors:** Ezgi PEHLİVANLAR, Deniz Arca ÇAKIR, Sonia SANAJOU, Hülya TEZEL YALÇIN, Terken BAYDAR, Pınar ERKEKOĞLU, Hanife AVCI, Rahime ŞİMŞEK

**Affiliations:** 1 Department of Pharmaceutical Chemistry, Faculty of Pharmacy, Hacettepe University, Ankara, Turkiye; 2 Department of Toxicology, Faculty of Pharmacy, Hacettepe University, Ankara, Turkiye; 3 Department of Vaccine Technology, Faculty of Pharmacy, Hacettepe University, Ankara, Turkiye; 4 Department of Biostatistics, School of Medicine, Hacettepe University, Ankara, Turkiye

**Keywords:** 1, 4-Dihydropyridines, hexahydroquinoline, cytotoxicity, inflammation, molecular docking

## Abstract

Inflammation is a response to injury and infection in an organism. It can be categorized as acute or chronic. Chronic inflammation is the underlying cause of many diseases such as Alzheimer disease, diabetes, rheumatoid arthritis, atherosclerosis, and cardiovascular diseases. Recent studies have proven the antiinflammatory properties of 1,4-dihydropyridines (1,4-DHPs) and their derivatives, which have many biological activities including the blocking of calcium channels. In this study, 15 compounds that are condensed derivatives of 1,4-DHPs, with the general structure of hexahydroquinoline-3-carboxylate, were synthesized. These compounds, expected to show inhibitory activity against inflammatory mediators, were obtained by the reaction of 4-(difluoromethoxy)benzaldehyde, substituted/nonsubstituted 1,3-cyclohexanedione derivatives, and appropriate alkyl acetoacetate compounds in the presence of ammonium acetate as a nitrogen source according to the Hantzsch synthesis method. The structures of the synthesized compounds were elucidated by IR, ^1^H NMR, ^13^C NMR, and HRMS methods. The cytotoxic properties of the compounds were determined by 3-(4,5-dimethylthiazol-2-yl)-2,5-diphenyltetrazolium bromide (MTT) method in the 3T3 cell line. Among the 15 compounds, the three compounds with the lowest levels of cytotoxic effects were selected for further experiments. Inflammation was induced by lipoxygenase and the effects of the selected compounds on the levels of reactive oxygen species, cytokines, and complement C3 and C9 regulatory proteins were investigated. It was found that the three selected compounds decreased the levels of transforming growth factor-beta 1 (TGF-β1). Among these compounds, compound **3e** provided the most significant decrease in this cytokine. Moreover, **3e** increased both C3 and C9 levels. Molecular modeling studies also showed that **3e** had better affinity for TGF-β1. When the binding modes of these compounds in the active site of TGF-β1 were analyzed, it was found that compound **3e** had hydrophobic interactions with amino acids Leu142, Tyr84, and Ile13; halogen bond interactions with Asp92; and hydrogen bond interactions with Ser89, Gly88, and Gly14 in the active binding site. Further in vitro and in vivo studies are needed to show the possible mechanism of action of compound **3e**.

## 1. Introduction

**Figure 1:**
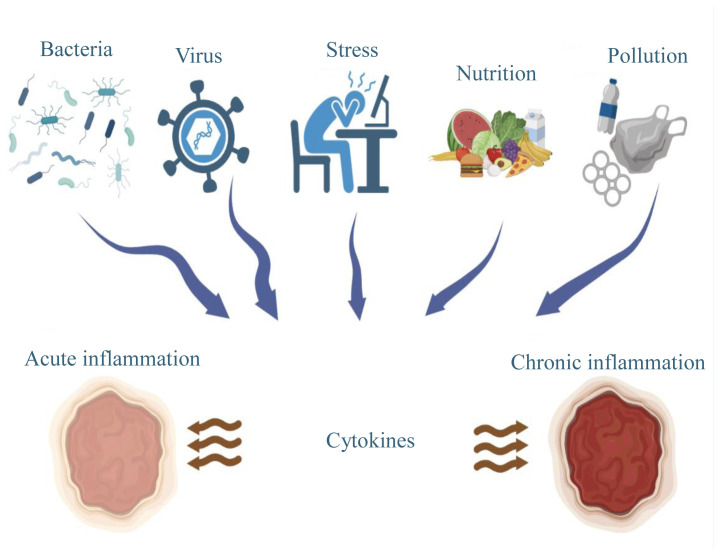
Inflammation mechanisms.

Inflammation is known as the body’s response to an injury or infection. Biological, chemical, and physical stress are factors that can trigger inflammation together with malnutrition and environmental pollution (Figure 1) [1]. However, inflammation can also develop without tissue damage as a result of inflammatory signals [2]. Senescent cells with impaired homeostasis may stimulate the release of inflammatory mediators through various signals [3]. Ischemia as well as toxins can also stimulate inflammatory mediators. Inflammation that occurs as a result of such stimuli causes the formation of an immune response, which in turn can enable the repair of damaged cells [4]. Cellular inflammation can become permanent and chronic inflammation may develop if treatment is not provided [5].

Chronic inflammation can lead to genotoxicity and, finally, mutation. The accumulation of mutations can be the underlying cause of many diseases [6]. Alzheimer disease, cancer, diabetes, atherosclerosis, arthritis, and pulmonary, autoimmune, and cardiovascular diseases may be associated with chronic inflammation [7]. Cytokines, including tumor necrosis factor-α (TNF-α), interleukin (IL)-1β, and IL-6, are among the most important mediators that generate an inflammatory response. IL-6 is the most important proinflammatory cytokine that plays a major role in cancer, cytokine storms, and autoimmune and inflammatory diseases [8–17].

**Figure 2:**
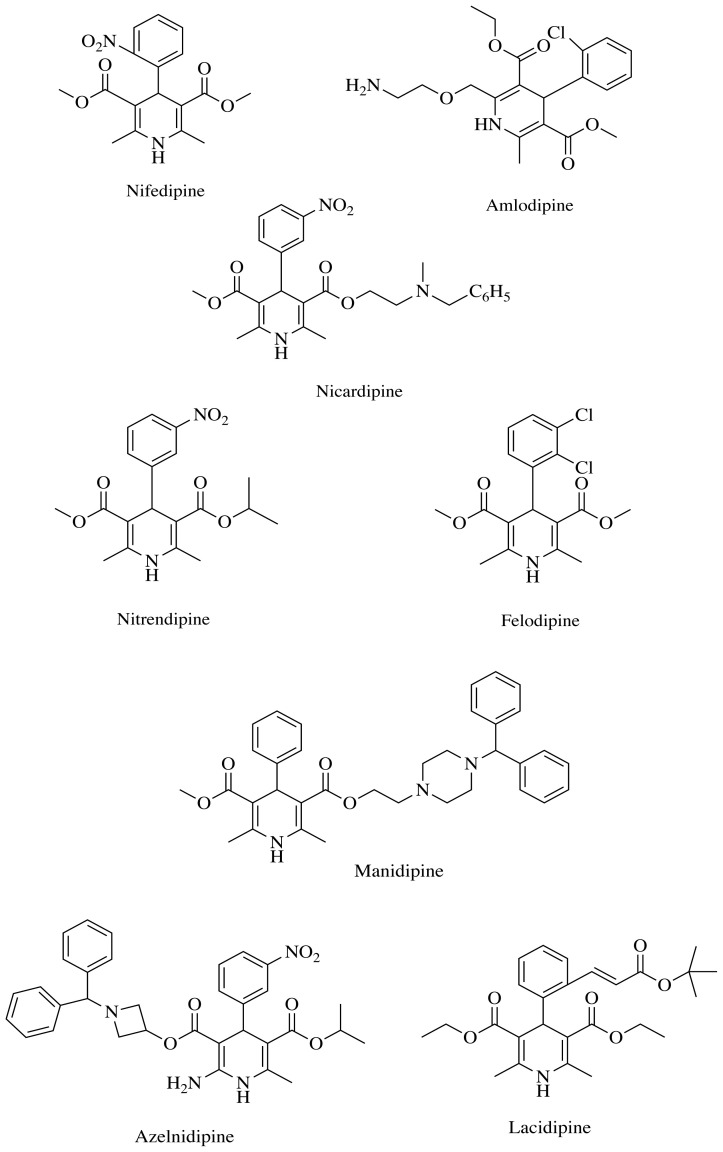
1,4-DHP derivatives used as antiinflammatory and immuno1ressive drugs.

Heterocyclic compounds are commonly used in the pharmaceutical industry as they have various biological activities [18]. For this reason, innovative and creative steps have been taken by researchers over the years in the development of new drug molecules with heterocyclic compounds. Due to their possible biological and pharmacological effects, heterocyclic compounds containing nitrogen atoms have been widely investigated in recent years. With the discovery of 1,4-dihydropyridines (1,4-DHPs), compounds containing heterocyclic rings, medicinal chemistry gained an important compound structure [19]. 1,4-DHPs, which have many synthesis methods, are biologically active compounds [20]. These compounds have been proven to have antiinflammatory, antitumor, antihypertensive, anti-Alzheimer, analgesic, and calcium channel-blocking effects [21]. Recent studies have shown that 1,4-DHP derivatives used as calcium channel blockers, such as nifedipine, amlodipine, nicardipine, nitrendipine, felodipine, manidipine, azelnidipine, and lacidipine, also have antiinflammatory and immunosuppressive effects (Figure 2) [22]. Although compounds with 1,4-DHP structures are mainly used as calcium channel blockers, they have been investigated for other indications in recent years. The effects of these compounds on inflammation mediators have also been reported [23]. It has been shown that cytokines can modulate cardiovascular function, and some drugs used in the treatment of heart failure variably modulate cytokine production. It has been reported that nifedipine, used as an antihypertensive drug, prevents nuclear factor-κB (NF-κB) activation; amlodipine inhibits TNF-α production; azelnidipine, a new-generation 1,4-DHP derivative, significantly decreases IL-8 levels; manidipine inhibits the release of IL-6 and IL-8; and lacidipine inhibits NF-κB [24–27].

**Figure 3:**
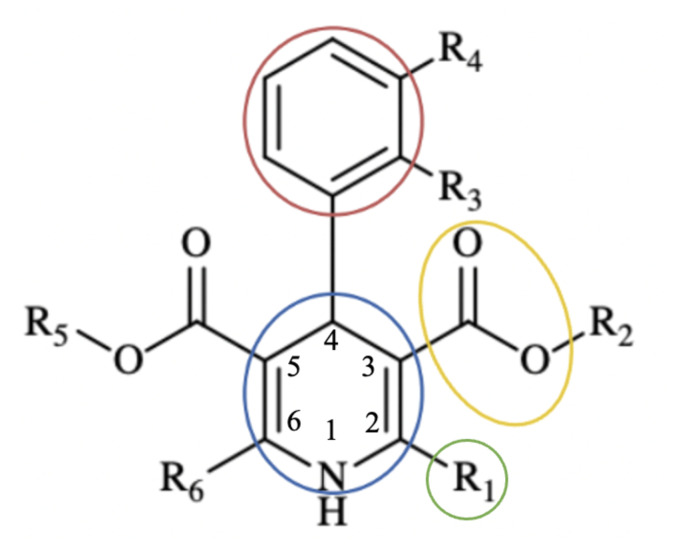
Common pharmacophore groups of 1,4-DHP derivatives known to modulate mediators of inflammation.

In light of this information, since the 1,4-DHP ring is open for various modifications and many compounds carrying this ring system have antiinflammatory activities, it can be suggested that new derivatives may also have such beneficial effects. The common features of 1,4-DHP derivatives with the potential to inhibit inflammation mediators are the presence of a methyl group at position 2 and/or 6, an ester group at position 3 and/or 5 of the ring, and a substituted aryl structure at position 4 (Figure 3).

**Figure 4:**
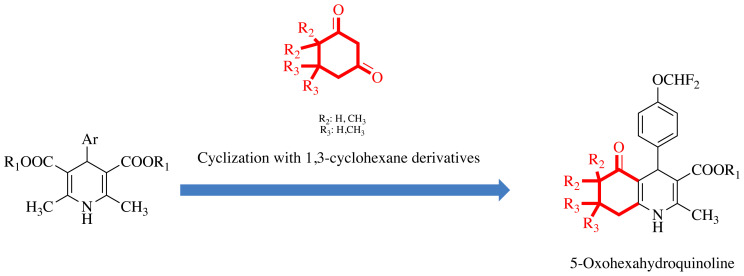
Design strategy of synthesized compounds.

Heterocyclic rings containing nitrogen atoms, such as quinolines, are known to play important roles in medicinal chemistry [28,29]. Quinolines may exert various biological activities, including antibacterial, antineoplastic, antileishmanial, antifungal, antidiabetic, antituberculosis, anti-Alzheimer, and antiinflammatory effects [30,31]. Hexahydroquinoline, formed by the condensation of a 1,4-DHP ring with a cyclohexane ring, is a heterocyclic ring with significant biological activities (Figure 4) [32–36]. Currently, 15% of the drug molecules available carry at least one fluorine atom. Fluorine is known to cause favorable changes in the physicochemical and pharmacokinetic parameters of a molecule and make compounds resistant to biotransformation reactions [37–40].

In this study, 15 compounds with the general structure of hexahydroquinoline-3-carboxylate, designed as analogs to 1,4-DHP derivatives, were synthesized. These compounds were expected to inhibit inflammation mediators and the structures of the compounds were elucidated by IR, ^1^H NMR, ^13^C NMR, and HRMS methods. All NMR spectra of the compounds are reported in the Supplementary Information.

Two of these compounds have been previously published with X-ray analysis data [41,42]. For the completeness of the study and for the purpose of presenting the results of biological activity, they were also included in the current work. The cytotoxic properties of the compounds were examined by 3-(4,5-dimethylthiazol-2-yl)-2,5-diphenyltetrazolium bromide (MTT) assay in the 3T3 cell line. Among the 15 compounds, the three compounds that exerted the lowest levels of cytotoxic effects were selected for further experiments. Inflammation was induced by lipoxygenase (LPS). The effects of the selected compounds on the levels of reactive oxygen species (ROS), cytokines, and complement C3 and C9 proteins after the induction of inflammation were investigated [43].

## 2. Materials and methods

### 2.1. Cell line, chemicals, and kits

The NIH 3T3 mouse fibroblast cell line (ATCC CRL-1658) was purchased from the American Type Culture Collection (ATCC) (Manassas, VA, USA). Penicillin-streptomycin, trypsin-ethylenediaminetetraacetic acid (EDTA), Dulbecco’s modified Eagle medium (DMEM), fetal bovine serum (FBS), and Dulbecco’s phosphate-buffered saline (DPBS) were obtained from Biowest (Nuaillé, France).

1,3-Cyclohexadione, 4,4-dimethyl-1,3-cyclohexadione, 5,5-dimethyl-cyclohexadione, 4-(difluoromethoxy)benzaldehyde, and alkyl acetoacetate derivatives were obtained from Sigma-Aldrich (Manheim, Germany). Ethyl acetate and n-hexane were obtained from Merck (Darmstadt, Germany). Kieselgel 60 F254 ready-made thin-layer chromatography (TLC) plates were also from Merck. MTT, a protein quantification kit, and ROS kit were purchased from Sigma-Aldrich. TGF-β1, IL-1a, IL-10, TNF-α, C3, and C9 kits were purchased from BT Lab (Birmingham, UK).

### 2.2. Measurements

The IR spectra of the synthesized compounds were taken with powder sample analysis and wave numbers were measured with Fourier transform infrared spectroscopy (FTIR) spectrum BX (PerkinElmer, Waltham, MA, USA) and IRAffinity-1S (Shimadzu, Kyoto, Japan) spectrophotometers. Values are shown in cm^−1^. The ^1^H NMR and ^13^C NMR spectra of the synthesized compounds were taken in deuterated dimethyl sulfoxide (DMSO-*d_6_*) with reference to tetramethylsilane (TMS) as a standard and evaluated on the δ scale by NMR (Varian Mercury 400 MHz, Agilent, Palo Alto, CA, USA; Avance Neo 500 MHz, Bruker, Billerica, MA, USA). Mass spectrum data of the synthesized compounds were obtained with a mass quadrupole time-of-flight (Q-TOF) device (6530 Accurate, Agilent). In silico data of the substances were calculated with the SwissADME program.

### 2.3. Biological activity

#### 2.3.1. Study groups

The NIH 3T3 mouse fibroblast cell line was used throughout the experiments. These cells were isolated from a *Mus musculus* embryo and consist of adherent cells with characteristics similar to those of human skin dermal fibroblasts. They were grown in flasks by adding 10% FBS and 1% penicillin-streptomycin to DMEM-low glucose medium. Cells were incubated in an incubator at 37 °C with 5% CO_2_ and were subcultured 2 or 3 times a week.

The study groups were as follows:

1. Control: Only the medium was applied.
2. LPS-applied group (L): Cells were incubated with LPS (400 ng/mL) for 24 h.
3. Compound **3e**-applied group (**3e**): Cells were incubated with LPS (400 ng/mL) and compound **3e** (40.90 μM) for 24 h.
4. Compound **3b**-applied group (**3b**): Cells were incubated with LPS (400 ng/mL) and compound **3b** (70.35 μM) for 24 h.
5. Compound **2d**-applied group (**2d**): Cells were incubated with LPS (400 ng/mL) and compound **2d** (57.93 μM) for 24 h.

The dose of LPS was chosen according to Li et al. [43].

#### 2.3.2. MTT assay

After incubating cells with various concentrations (0, 1, 5, 10, 12.5, 12.5, 25, 50, 75, 150, 150, and 200 μM) of compounds **2d**, **3b**, and **3e**, the standard MTT procedure was performed by adding MTT solution (1 mg/mL) and absorbance was measured at 570 nm with a SpectraMax M2 spectrophotometer (Molecular Devices, Sunnyvale, CA, USA). The control cells were assumed to have 100% viability and the viability of other study groups was calculated as a percentage compared to the control. The 50% inhibitory concentration (IC_50_) and 30% inhibitory concentration (IC_30_) values were calculated for each compound. For subsequent analyses, the IC_30_ values derived from MTT assay results were applied to the cells. The experiments were repeated three times on different days with two replicates on the same day. The mean of all experiments was calculated.

#### 2.3.3. Intracellular ROS determination

A ROS kit was used for the determination of intracellular ROS levels in single-step fluorometric determination of intracellular ROS with 1 h of incubation. With this kit, ROS molecules in the cell react with the cell-permeable sensor and generate a product that can be obtained by fluorometric measurement at l_excitation_ = 540 nm and l_emission_ = 570 nm. ROS levels of the control cells were assumed to be 100% and the ROS levels of other groups were expressed as a percentage of the control.

#### 2.3.4. Preparation of cell lysates

After the incubation period, media were removed and cell lysates were prepared. The lysis buffer contained a protease inhibitor cocktail (1 mL of protease inhibitor/100 mL of lysis buffer). After adding 800 μL of cell lysis buffer, cell pellets were centrifuged at 13,000 rpm for 15 min. Supernatants were collected and stored at −80 °C until analysis.

#### 2.3.5. Collection of cell culture media

At the end of the incubation period, the cell culture media of each group were collected and centrifuged at 1000 × *g* for 20 min at 2–8 °C. Parameters were then measured in the collected supernatants.

#### 2.3.6. Determination of inflammation markers and complement proteins

Levels of inflammation markers (IL-1α, IL-10, TGF-β1, and TNF-α) and complement proteins (C3 and C9) were measured in the cell culture supernatants using commercial ELISA kits. These kits use the sandwich-ELISA principle. Briefly, plates were precoated with specific antibodies related to the measured biomarkers. Samples were first added to wells and the specific proteins bound to the antibodies coated on the wells. Biotinylated antibodies that bound to the measured proteins were then added. Streptavidin-HRP, which binds to the biotinylated antibodies, was then added. After incubation, the unbound streptavidin-HRP was washed away during a washing step. Substrate solution was then added and color developed in proportion to the amount of measured protein. The reaction was terminated by the addition of an acidic stop solution and absorbance was measured at 450 nm.

#### 2.3.7. Total protein levels

Protein determination was performed using a kit based on the bicinchoninic acid (BCA) method. Briefly, the BCA protein assay combines the well-known reduction of Cu^2+^ to Cu^+^ by protein in an alkaline medium (also known as the biuret reaction) with the highly sensitive and selective colorimetric detection of the cuprous cation (Cu^+^) by BCA. Absorbance of the samples was measured at 562 nm and protein levels were calculated using bovine serum albumin (BSA) standards.

#### 2.3.8. Molecular docking analysis

Molecular modeling studies were carried out using the AutoDock Vina program to determine the interactions of the compounds synthesized within the scope of the study with the TGF-β1 enzyme active site amino acids at the molecular level [44]. The X-ray structure of the TGF-β1 enzyme (PDB: 4X2F) was downloaded from the RCSB Protein Data Bank database and the water molecules in the crystal structure were deleted in the Discovery Studio 2021 program; this process was completed by correcting incorrect and missing atoms and adding polar hydrogen atoms and Kollman energy minimization in the AutoDock Tools 1.5.7 program. The coordinates of the inhibitor compound based on the enzyme X-ray crystal structure were determined using the Discovery Studio 2021 program, and a grid of 30 × 30 × 30 Å^3^ was created for the active binding site in the AutoDock Tools 1.5.7 program. The resulting grid file was saved to be used in molecular docking studies. The compounds drawn in the Maestro interface of the Schrödinger program were transformed into their three-dimensional structures with default values using the LigPrep module (Schrödinger Release 2024-2: LigPrep, Schrödinger LLC, New York, NY, USA). To verify the molecular docking protocol to be applied, the coligands in the X-ray crystal structure of the TGF-β1 enzyme were repositioned into the relevant enzyme active sites, and the conformations obtained as a result of this process overlapped with the bioactive conformations of the relevant coligand, giving a RMSD value of 0.330 Å (PDB: 4X2F) and indicating that accuracy of the protocol was achieved. The placement of the compounds in the TGF-β1 active site was carried out using the Vina module of the AutoDock Vina program with the previously saved grid file. The obtained results were evaluated based on the connection energies and interaction types expressed in XP GScore values and the appropriate connection modes were determined.

#### 2.3.9. Statistical analysis

Statistical analysis was performed using IBM SPSS Statistics 23 (IBM Corp., Armonk, NY, USA). The differences among the groups were evaluated with Kruskal–Wallis one-way analysis of variance, followed by Mann–Whitney U tests. Results are expressed as mean ± standard deviation (SD). Values of p < 0.05 were considered statistically significant.

## 3. Results and discussion

### 3.1. Chemistry

**Figure 5:**
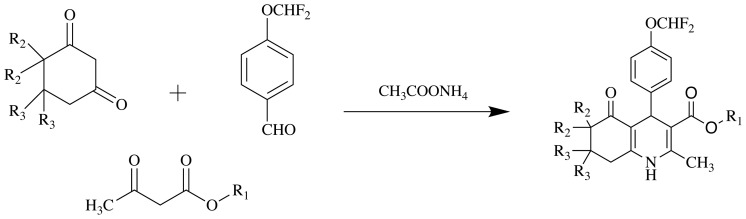
Synthesis of compounds **1a**–**3e. R_1_**: alkyl; **R_2_**, **R_3_**: methyl.

For the synthesis of compounds **1a**–**1e**, **2a**–**2e**, and **3a**–**3e**, 1 mmol 1,3-cyclohexadione derivative, 1 mmol 4-(difluoromethoxy)benzaldehyde, 1 mmol appropriate alkyl acetoacetate derivative, and 5 mmol ammonium acetate as a nitrogen source in 10 mL of methanol were heated by the Hantzsch procedure under reflux (Figure 5). The end of the reaction was monitored by TLC using an ethyl acetate and n-hexane (1:1) solvent system. The resulting compounds were purified by crystallization from methanol. Compound **2d** was previously synthesized and published by our research group, but it is included in the scope of this study for series integrity. All NMR spectra of the compounds are reported in the Supplementary Information.

### 3.2. Spectral data

Methyl 4-[4-(difluoromethoxy)phenyl]-2-methyl-5-oxo-1,4,5,6,7,8-hexahydroquinoline-3-carboxylate (**1a**): Yield: 20%; yellow solid; mp 175–176 °C; IR (ν, cm^−1^) 3186 (N-H stretching); 3071 (C-H stretching, aromatic); 2952 (C-H stretching, aliphatic); 1698 (C=O stretching, ester); 1651 (C=O stretching, ketone). ^1^H NMR (400 MHz, DMSO-*d_6_*, ppm) δ 1.73–1.77 (2H; *m*; quinoline H7), 1.85–1.91 (2H; *m*; quinoline H8), 2.12–2.23 (2H; *m*; quinoline H6), 2.26 (3H; *s*; 2-CH_3_), 3.51 (3H; *s*; COOCH_3_), 4.88 (1H; *s*; quinoline H4), 6.96 (2H; *d*; *J* = 8.4 Hz; Ar-H3, Ar-H5), 7.08 (1H; *t*; *J* = 74.4 Hz; OCHF_2_), 7.14 (2H; *d*; *J* = 8.4 Hz; Ar-H2, Ar-H6), 9.12 (1H; *s*; NH). ^13^C NMR (100 MHz, DMSO-*d_6_*, ppm): δ 18.2 (2-CH_3_), 20.7 (C-7), 26.0 (C-8), 34.8 (C-6), 36.5 (C-4), 50.6 (COOCH_3_), 102.9 (C-3), 110.9 (C-4a), 113.8 (C_3_′), 116.4, 118.3, 118.9 (OCHF_2_), 128.6 (C_2_′), 144.6 (C_1_′), 145.3 (C-2), 149.8 (C-8a), 151.4 (C_4_′), 167.2 (COOCH_3_), 194.6 (C-5). HRMS (ESI/Q-TOF): *m/z* calculated for C_19_H_19_F_2_NO_4_ [M + H]^+^ found 364.1435.

Ethyl 4-(4-(difluoromethoxy)phenyl)-2-methyl-5-oxo-1,4,5,6,7,8-hexahydroquinoline-3-carboxylate (**1b**): Yield: 23%; yellow solid; mp: 187–188 °C; IR (ν, cm^−1^) 3283 (N-H stretching); 3077 (C-H stretching, aromatic); 2959 (C-H stretching, aliphatic); 1683 (C=O stretching, ester); 1645 (C=O stretching, ketone). ^1^H NMR (500 MHz, DMSO*-d_6_*, ppm): δ 1.12 (2H; *t*; *J* = 7 Hz; COOCH_2_CH_3_), 1.74–1.76 (2H; *m*; quinoline H7), 1.88–1.91 (2H; *m*; quinoline H8), 2.15–2.22 (2H; *m*; quinoline H6), 2.28 (3H; *s*; 2-CH_3_), 3.98 (3H; *q*; *J* = 7 Hz; COOCH_2_CH_3_), 4.89 (1H; *s*; quinoline H4), 6.99 (2H; *d*; *J* = 8 Hz; Ar-H3, Ar-H5), 7.13 (1H; *t*; *J* = 74.4 Hz; OCHF_2_), 7.17 (2H; *d*; *J* = 8 Hz; Ar-H2, Ar-H6), 9.16 (1H; *s*; NH). ^13^C NMR (125 MHz, DMSO-*d_6_*, ppm): δ 14.6 (COOCH_2_CH_3_), 18.7 (2-CH_3_), 21.2 (C-7), 26.5 (C-8), 35.5 (C-6), 37.1 (C-4), 59.5 (COOCH_2_CH_3_), 103.7 (C-3), 111.4 (C-4a), 114.9 (C_3_′), 116.9, 118.7, 118.9 (OCHF_2_), 129.3 (C_2_′), 145.3 (C_1_′), 145.6 (C-2), 149.3 (C-8a), 151.9 (C_4_′), 167.2 (COOCH_2_CH_3_), 196.1 (C-5). HRMS (ESI/Q-TOF): *m/z* calculated for C_19_H_19_F_2_NO_4_ [M + H]^+^ found 378.1563.

Isopropyl 4-(4-(difluoromethoxy)phenyl)-2-methyl-5-oxo-1,4,5,6,7,8-hexahydroquinoline-3-carboxylate (**1c**): Yield: 27%; yellow solid; mp 188–189 °C; IR (ν, cm^−1^) 3197 (N-H stretching); 3070 (C-H stretching, aromatic); 2981 (C-H stretching, aliphatic); 1695 (C=O stretching, ester); 1651 (C=O stretching, ketone). ^1^H NMR (400 MHz, DMSO*-d_6_*, ppm): δ 1.00 (3H; *d*; *J* = 6; COOCH(CH_3_)_2a_), 1.14 (3H; *d*; *J* = 6; COOCH(CH_3_)_2b_), 1.72–1.74 (2H; *m*; quinoline H7), 1.84–1.90 (2H; *m*; quinoline H8), 2.15–2.21 (2H; *m*; quinoline H6), 2.25 (3H; *s*; 2-CH_3_), 4.75–4.81 (H; *m*; COOCH(CH_3_)_2_), 4.83 (H; *s*; quinoline H4), 6.96 (2H; *d*; *J* = 8 Hz; Ar-H3, Ar-H5), 7.09 (1H; *t*; *J* = 74.4 Hz; OCHF_2_), 7.15 (2H; *d*; *J* = 8 Hz; Ar-H2, Ar-H6), 9.08 (1H; *s*; NH). ^13^C NMR (100 MHz, DMSO-*d_6_*, ppm): δ 18.1 (2-CH_3_), 20.7 (C-7), 21.4 (COOCH(CH_3_)_2a_), 21.7 (COOCH(CH_3_)_2b_), 26.0 (C-8), 35.2 (C-6), 36.6 (C-4), 66.0 (COOCH(CH_3_)), 103.6 (C-3), 110.8 (C-4a), 113.8 (C_3_′), 116.4, 118.5, 118.9 (OCHF_2_), 128.9 (C_2_′), 144.7 (C_1_′), 144.9 (C-2), 148.8 (C-8a), 151.4 (C_4_′), 166.2 (COOCH(CH_3_)_2_), 194.5 (C-5). HRMS (ESI/Q-TOF): *m/z* calculated for C_21_H_23_F_2_NO_4_ [M + H]^+^ found 392.1736.

Isobutyl 4-(4-(difluoromethoxy)phenyl)-2-methyl-5-oxo-1,4,5,6,7,8-hexahydroquinoline-3-carboxylate (**1d**): Yield: 20%; yellow solid; mp 188–189 °C; IR (ν, cm^−1^) 3296 (N-H stretching); 3081 (C-H stretching, aromatic); 2962 (C-H stretching, aliphatic); 1689 (C=O stretching, ester); 1651 (C=O stretching, ketone). ^1^H NMR (500 MHz, DMSO*-d_6_*, ppm): δ 0.78 (3H; *d*; COOCH_2_CH(CH_3_)_2a_), 0.79 (3H; *d*; COOCH_2_CH(CH_3_)_2a_) 1.78–1.80 (H; *m*; COOCH_2_CH(CH_3_)_2_), 1.87–1.92 (2H; *m*; quinoline H7), 2.17–2.22 (2H; *m*; quinoline H8), 2.31 (3H; *s*; 2-CH_3_), 2.45–2.51 (2H; *m*; quinoline H6), 3.75–3.71 (2H; *m*; COOCH_2_CH(CH_3_)_2_), 4.92 (H; *s*; quinoline H4), 6.99 (2H; *d*; *J* = 8 Hz; Ar-H3, Ar-H5), 7.11 (1H; *t*; *J* = 74.4 Hz; OCHF_2_), 7.18 (2H; *d*; *J* = 8 Hz; Ar-H2, Ar-H6), 9.17 (H; *s*; NH). ^13^C NMR (125 MHz, DMSO-*d_6_*, ppm): δ 18.8 (2-CH_3_), 19.4 (COOCH_2_CH(CH_3_)_2_), 21.2 (C-7), 26.5 (C-8), 27.7 (COOCH_2_CH(CH_3_)_2_), 35.5 (C-6), 37.1 (C-4), 69.7 (COOCHCH_2_(CH_3_)_2_), 103.4 (C-3), 111.5 (C-4a), 114.8 (C_3_′), 116.9, 118.8, 118.9 (OCHF_2_), 129.2 (C_2_′), 145.2 (C_1_′), 146.0 (C-2), 149.3 (C-8a), 151.7 (C_4_′), 167.2 (COOCH_2_CH(CH_3_)_2_), 195.1 (C-5). HRMS (ESI/Q-TOF): *m/z* calculated for C_22_H_25_F_2_NO_4_ [M + H]^+^ found 406.1794.

Tert-butyl 4-(4-(difluoromethoxy)phenyl)-2-methyl-5-oxo-1,4,5,6,7,8-hexahydroquinoline-3-carboxylate (**1e**): Yield: 23%; yellow solid; mp 174–175 °C; IR (ν, cm^−1^) 3208 (N-H stretching); 3077 (C-H stretching, aromatic); 2974 (C-H stretching, aliphatic); 1701 (C=O stretching, ester); 1670 (C=O stretching, ketone). ^1^H NMR (500 MHz, DMSO*-d_6_*, ppm): δ 1.29 (9H; *s*; COOC(CH_3_)_3_), 1.85–1.88 (2H; *m*; quinoline H7), 2.13–2.18 (2H; *m*; quinoline H8), 2.43–2.45 (2H; *m*; quinoline H6), 2.22 (3H; *s*; 2-CH_3_), 4.80 (H; *s*; quinoline H4), 6.97 (2H; *d*; *J* = 8 Hz; Ar-H3, Ar-H5), 7.08 (H; *t*; *J* = 74.4 Hz; OCHF_2_), 7.15 (2H; *d*; *J* = 8 Hz; Ar-H2, Ar-H6), 8.98 (H; *s*; NH). ^13^C NMR (125 MHz, DMSO-*d_6_*, ppm): δ 18.2 (2-CH_3_), 20.7 (C-7), 26.1 (COOC(CH_3_)_3_, 27.8 (C-8), 35.5 (C-6), 36.6 (C-4), 78.8 (COOC(CH_3_)_3_), 104.8 (C-3), 110.7 (C-4a), 113.9 (C_3_′), 116.4, 118.1, 119.0 (COHF_2_), 128.9 (C_2_′), 144.0 (C_1_′), 144.9 (C-2), 148.8 (C-8a), 151.4 (C_4_′), 166.2 (COOC(CH_3_)_3_), 194.5 (C-5). HRMS (ESI/Q-TOF): *m/z* calculated for C_22_H_25_F_2_NO_4_ [M + H]^+^ found 406.1896.

Methyl 4-(4-(difluoromethoxy)phenyl)-2,6,6-trimethyl-5-oxo-1,4,5,6,7,8-hexahydroquinoline-3-carboxylate (**2a**): Yield: 42%; white solid; mp 176–177 °C; IR (ν, cm^−1^) 3302 (N-H stretching); 2931 (C-H stretching, aliphatic); 1697 (C=O stretching, ester); 1643 (C=O stretching, ketone). ^1^H NMR (500 MHz, DMSO-*d_6_*, ppm): δ 0.90 (3H; *s*; 6-CH_3_), 0.98 (3H; *s*; 6-CH_3_), 1.71–1.72 (2H; *m*; quinoline H7), 2.50–2.52 (2H; *m*; quinoline H8), 2.28 (3H; *s*; 2-CH_3_) 3.54 (3H; *s*; COOCH_3_), 4.87 (H; *s*; quinoline H4), 6.98 (2H; *d*; *J* = 9 Hz; Ar-H3, Ar-H5), 7.13 (1H; *t*; *J* = 74.4 Hz; OCHF_2_), 7.15 (2H; *d*; *J* = 9 Hz Ar-H2, Ar-H6), 9.14 (1H; *s*; NH). ^13^C NMR (125 MHz, DMSO-*d_6_*, ppm): δ 18.7 (2-CH_3_), 23.3 (C-8), 24.5 (6-CH_3_), 25.5 (C-7), 34.5 (C-4), 35.6 (C-6), 51.1 (COOCH_3_), 103.0 (C-3), 109.5 (C-4a), 114.8 (C_3_′), 116.9, 118.7, 118.9 (OCHF_2_), 129.0 (C_2_′), 145.1 (C_1_′), 145.9 (C-2), 149.3 (C-8a), 150.2 (C_4_′), 167.7 (COOCH_3_), 199.9 (C-5). HRMS (ESI/Q-TOF): *m/z* calculated for C_21_H_23_F_2_NO_4_ [M + H]+ found 392.1834.

Ethyl 4-(4-(difluoromethoxy)phenyl)-2,6,6-trimethyl-5-oxo-1,4,5,6,7,8-hexahydroquinoline-3-carboxylate (**2b**): Yield: 20%; white solid; mp: 208–209 °C; IR (ν, cm^−1^) 3300 (N-H stretching); 2987 (C-H stretching, aromatic); 2935 (C-H stretching, aliphatic); 1733 (C=O stretching, ester); 1651 (C=O stretching, ketone). ^1^H NMR (500 MHz, DMSO-*d_6_*, ppm): δ 0.89 (3H; *s*; 6-CH_3_), 0.98 (3H; *s*; 6-CH_3_), 1.13 (3H; *t*; *J* = 7.1 Hz; COOCH_2_CH_3_), 1.69–1.73 (2H; *m*; quinoline H7), 2.50–2.51 (2H; *m*; quinoline H8), 2.27 (3H; *s*; 2-CH_3_), 3.98 (2H; *q*; *J* = 7 Hz; COOCH_2_CH_3_), 4.85 (1H; *s*; quinoline H4), 6.98 (2H; *d*; *J* = 8.5 Hz; Ar-H3, Ar-H5), 7.14 (1H; *t*; *J* = 74.4 Hz; OCHF_2_), 7.15 (2H; *d*; *J* = 8.5 Hz; Ar-H2, Ar-H6), 9.14 (1H; *s*; NH). HRMS (ESI/Q-TOF): *m/z* calculated for C_22_H_25_F_2_NO_4_ [M + H]+ found 406.1991.

Isopropyl 4-(4-(difluoromethoxy)phenyl)-2,6,6-trimethyl-5-oxo-1,4,5,6,7,8-hexahydroquinoline-3-carboxylate (**2c**): Yield: 37%; white solid; mp 213–214 °C; IR (ν, cm^−1^) 3194 (N-H stretching); 2970 (C-H stretching, aromatic); 2939 (C-H stretching, aliphatic); 1674 (C=O stretching, ester). ^1^H NMR (400 MHz, DMSO-*d_6_*, ppm): δ 0.86 (3H; *s*; 6-CH_3_), 0.96 (3H; *s*; 6-CH_3_), 1.0 (3H; *d*; *J* = 6.4 Hz; COOCH(CH_3_)_2a_), 1.15 (3H; *d*; *J* = 6.4 Hz; COOCH(CH_3_)_2b_), 1.67–1.70 (2H; *m*; quinoline H7), 2.48 (2H; *m*; quinoline H8), 2.24 (3H; *s*; 2-CH_3_), 4.77–4.82 (1H; *m*; COOCH(CH_3_)_2_), 4.81 (1H; *s*; quinoline H4), 6.95 (2H; *d*; *J* = 8 Hz; Ar-H3), 7.09 (1H; *t*; *J* = 74.4 Hz; OCHF_2_), 7.14 (2H; *d*; *J* = 8 Hz; Ar-H2, Ar-H6), 9.01 (1H; *s*; NH). ^13^C NMR (100 MHz, DMSO-*d_6_*, ppm): δ 18.2 (2-CH_3_), 21.5 (COOCH(CH_3_)_2a_), 21.8 (COOCH(CH_3_)_2b_), 22.8 (C-8), 24.0 (6-CH_3_), 25.0 (C-7), 34.0 (C-4), 35.5 (C-6), 66.0 (COOCH(CH_3_)_2_), 103.3 (C-3), 108.9 (C-4a), 113.8 (C_3_′), 116.6, 118.0, 118.9 (OCHF_2_), 128.8 (C_2_′), 144.7 (C_1_′), 144.9 (C-2), 149.3 (C-8a), 149.7 (C_4_′), 166.2 (COOCH(CH_3_)_2_), 199.3 (C-5). HRMS (ESI/Q-TOF): *m/z* calculated for C_23_H_27_F_2_NO_4_ [M + H]+ found 420.2150.

Isobutyl 4-(4-(difluoromethoxy)phenyl)-2,6,6-trimethyl-5-oxo-1,4,5,6,7,8-hexahydroquinoline-3-carboxylate (**2d**) [39]: Yield: 23%; white solid; mp 179–180 °C; IR (ν, cm^−1^) 3193 (N-H stretching); 3073 (C-H stretching, aromatic); 2957 (C-H stretching, aliphatic); 1670 (C=O stretching, ester); 1646 (C=O stretching, ketone). ^1^H NMR (400 MHz, DMSO-*d_6_*, ppm): δ 0.77 (3H; *d*; *J* = 6.4 Hz; (OCH_2_CH(CH_3_)_2a_)), 0.78 (3H; *d*; *J* = 6.4 Hz; (OCH_2_CH(CH_3_)_2b_)), 0.86 (3H; *s*; 6-CH_3_), 0.96 (3H; *s*; 6-CH_3_), 1.64–1.72 (2H; *m*; (OCH_2_CH(CH_3_)_2_)), 1.74–1.79 (2H; *m*; quinoline H7), 2.44–2.47 (2H; *m*; quinoline H8), 2.28 (3H; *s*; 2-CH_3_), 3.66–3.75 (2H; *m*; (OCH_2_CH(CH_3_)_2_)), 4.86 (H; *s*; quinoline H4), 6.96 (2H; *d*; *J* = 8.2 Hz; Ar-H3), 7.09 (1H; *t*; *J* = 74.4 Hz; OCHF_2_), 7.14 (2H; *d*; *J* = 8.2 Hz; Ar-H2), 9.09 (1H; *s*; NH). ^13^C NMR (100 MHz, DMSO-*d_6_*, ppm): δ 18.32 (2-CH_3_), 18.91 (COOCHCH_2_(CH_3_)_2a_), 18.93 (COOCH_2_CH(CH_3_)_2b_), 22.8 (C-8), 24.0 (6-CH_3_), 25.0 (C-7), 27.2 (COOCH_2_CH(CH_3_)_2_), 34.0 (C-4), 35.2 (C-6), 69.2 (COOCHCH_2_(CH_3_)_2_) 102.6 (C-3), 109.1 (C-4a), 113.8 (C_3_′), 116.3, 118.1, 118.9 (OCHF_2_), 128.6 (C_2_′), 144.7 (C_1_′), 145.4 (C-2), 148.8 (C-8a), 149.5 (C_4_′), 166.8 (COOCHCH_2_(CH_3_)_2_), 199.4 (C-5). HRMS (ESI/Q-TOF): *m/z* calculated for C_24_H_29_F_2_NO_4_ [M + H]+ found 434.2319.

Tert-butyl 4-(4-(difluoromethoxy)phenyl)-2,6,6-trimethyl-5-oxo-1,4,5,6,7,8-hexahydroquinoline-3-carboxylate (**2e**): Yield: 20%; white solid; mp 183–184 °C; IR (ν, cm^−1^) 3194 (N-H stretching); 2962 (C-H stretching, aromatic); 2931 (C-H stretching, aliphatic); 1674 (C=O stretching, ester). ^1^H NMR (400 MHz, DMSO-*d_6_*, ppm): δ 0.86 (3H; *s*; 6-CH_3_), 0.95 (3H; *s*; 6-CH_3_), 1.30 (9H; *s*; COOC(CH_3_)_3_), 1.65–1.69 (2H; *m*; quinoline H7), 2.44–2.47 (2H; *m*; quinoline H8), 2.20 (3H; *s*; 2-CH_3_), 4.76 (1H; *s*; quinoline H4), 6.96 (2H; *d*; *J* = 8.4 Hz; Ar-H3, Ar-H5), 7.10 (1H; *t*; *J* = 74.4 Hz; OCHF_2_), 7.13 (2H; *d*; *J* = 8 Hz; Ar-H2, Ar-H6), 8.95 (1H; *s*; NH). ^13^C NMR (100 MHz, DMSO-*d_6_*, ppm): δ 18.1 (2-CH_3_), 22.8 (C-8), 24.0 (6-CH_3_), 25.0 (C-7), 27.8 (COOC(CH_3_)_3_), 34.0 (C-4), 35.7 (C-6), 78.7 (COOC(CH_3_)_3_), 104.4 (C-3), 108.7 (C-4a), 113.8 (C_3_′), 116.3, 118.0, 118.9 (OCHF_2_), 128.7 (C_2_′), 143.9 (C_1_′), 144.9 (C-2), 148.7 (C-8a), 149.7 (C_4_′), 166.3 (COOC(CH_3_)_3_), 199.2 (C-5). HRMS (ESI/Q-TOF): *m/z* calculated for C_24_H_29_F_2_NO_4_ [M + H]+ found 434.2321.

Methyl 4-(4-(difluoromethoxy)phenyl)-2,7,7-trimethyl-5-oxo-1,4,5,6,7,8-hexahydroquinoline-3-carboxylate (**3a**): Yield: 59%; yellow solid; mp 205–206 °C; IR (ν, cm^−1^) 3208 (N-H stretching); 3076 (C-H stretching, aromatic); 2956 (C-H stretching, aliphatic); 1700 (C=O stretching, ester); 1649 (C=O stretching, ketone). ^1^H NMR (500 MHz, DMSO-*d_6_*, ppm): δ 0.84 (3H; *s*; 7-CH_3_), 1.00 (3H; *s*; 7-CH_3_), 1.98 (1H; *d*; *J* = 16,05; quinoline H8b), 2.17 (1H; *d*; *J* = 16.05 Hz; quinoline H8a), 2.29 (3H; s; 2-CH_3_), 2.29 (1H; *d*; *J* = 16.05 Hz; quinoline H6a), 2.30 (2H; *d*; *J* = 16.05 Hz; quinoline H6b), 3.53 (3H; *s*; COOCH_3_), 4.86 (H; *s*; quinoline H4), 6.99 (2H; *d*; *J* = 8.6 Hz; Ar-H3, Ar-H5), 7.13 (1H; *t*; *J* = 74.4 Hz; OCHF_2_), 7.17 (2H; *d*; *J* = 8.6 Hz; Ar-H2, Ar-H6), 9.14 (1H; *s*; NH). ^13^C NMR (125 MHz, DMSO-*d_6_*, ppm): δ 18.8 (2-CH_3_), 26.9 (7-CH_3_), 29.5 (C-7), 32.6 (C-8), 35.6 (C-4), 50.6 (C-6), 51.1 (COOCH_3_), 103.4 (C-3), 110.2 (C-4a), 114.8 (C_3_′), 116.9, 118.9–118.6 (OCHF_2_), 129.2 (C_2_′), 145.0 (C_1_′), 145.9 (C-2), 149.4 (C-8a), 150.06 (C_4_′), 167.6 (COOCH_3_), 194.7 (C-5). HRMS (ESI/Q-TOF): *m/z* calculated for C_21_H_23_F_2_NO_4_ [M + H]+ found 392.1825.

Ethyl 4-(4-(difluoromethoxy)phenyl)-2,7,7-trimethyl-5-oxo-1,4,5,6,7,8-hexahydroquinoline-3-carboxylate (**3b**): Yield: 59%; yellow solid; mp 178–176 °C; IR (ν, cm^−1^) 3275 (N-H stretching); 3076 (C-H stretching, aromatic); 2965 (C-H stretching, aliphatic); 1700 (C=O stretching, ester); 1650 (C=O stretching, ketone). ^1^H NMR (500 MHz, DMSO-*d_6_*, ppm): δ 0.85 (3H; *s*; 7-CH_3_), 1.01 (3H; *s*; 7-CH_3_), 1.12 (3H; *t*; *J* = 7.1 Hz; COOCH_2_CH_3_), 1.98 (1H; *d*; *J* = 16 Hz; quinoline H8a), 2.15–2.18 (1H; *d*; *J* = 16.1 Hz; quinoline H8b), 2.28 (3H; *s*; 2-CH_3_), 2.29 (1H; *d*; *J* = 14 Hz; quinoline H6a), 2.41 (1H; *d*; *J* = 17; quinoline H6b), 3.97 (2H; *q*; *J* = 7 Hz; COOCH_2_CH_3_), 4.84 (H; *s*; quinoline H4), 6.99 (2H; *d*; *J* = 8.5 Hz; Ar-H3, Ar-H5), 7.13 (1H; *t*; *J* = 74.4 Hz; OCHF_2_), 7.17 (2H; *d*; *J* = 8.5 Hz; Ar-H2, Ar-H6), 9.10 (1H; *s*; NH). ^13^C NMR (125 MHz, DMSO-*d_6_*, ppm): δ 14.6 (COOCH_2_CH_3_), 18.7 (2-CH_3_), 27.0 (7-CH_3_), 29.5 (C-7), 32.6 (C-8), 35.8 (C-4), 50.6 (C-6), 59.5 (COOCH_2_CH_3_), 103.8 (C-3), 110.2 (C-4a), 114.8 (C_3_′), 116.9, 118.5, 118.9 (OCHF_2_), 129.3 (C_2_′), 145.1 (C_1_′), 145.6 (C-2), 149.4 (C-8a), 150.0 (C_4_′), 167.2 (COOCH_3_), 194.7 (C-5). HRMS (ESI/Q-TOF): *m/z* calculated for C_22_H_25_F_2_NO_4_ [M + H]+ found 406.1916.

Isopropyl 4-(4-(difluoromethoxy)phenyl)-2,7,7-trimethyl-5-oxo-1,4,5,6,7,8-hexahydroquinoline-3-carboxylate (**3c**): Yield: 44%; yellow solid; mp 216–217 °C; IR (ν, cm^−1^) 3184 (N-H stretching); 3072 (C-H stretching, aromatic); 2964 (C-H stretching, aliphatic); 1693 (C=O stretching, ester); 1650 (C=O stretching, ketone). ^1^H NMR (500 MHz, DMSO-*d_6_*, ppm): δ 0.85 (3H; *s*; 7-CH_3_), 1.01 (3H; *s*; 7-CH_3_), 1.03 (3H; *d*; *J* = 6.2 COOCH(CH_3_)_2a_), 1.17 (3H; *d*; *J* = 6.2 Hz COOCH(CH_3_)_2b_), 1.97 (1H; *d*; *J* = 16 Hz; quinoline H8a), 2.15 (1H; *d*; *J* = 16 Hz; quinoline H8b), 2.27 (3H; *s*; 2-CH_3_), 2.29 (1H; *d*; *J* = 17 Hz; quinoline H6a), 2.41 (1H; *d*; *J* = 17 Hz; quinoline H6b), 4.79–4.82 (1H; *m*; COOCH(CH_3_)_2_), 4.82 (H; *s*; quinoline H4), 6.99 (2H; *d*; *J* = 8.8 Hz; Ar-H3, Ar-H5), 7.13 (1H; *t*; *J* = 74.4 Hz; OCHF_2_), 7.17 (2H; *d*; *J* = 8.8 Hz; Ar-H2, Ar-H6), 9.06 (1H; *s*; NH). ^13^C NMR (125 MHz, DMSO-*d_6_*, ppm): δ 18.7 (2-CH_3_), 21.9 (COOCH(CH_3_)_a_), 22.3 (COOCH(CH_3_)_b_), 27.0 (7-CH_3_), 29.5 (C-7), 32.6 (C-8), 35.9 (C-4), 50.6 (C-6), 66.6 (COOCH(CH_3_)_2_), 104.2 (C-3), 110.2 (C-4a), 114.8 (C_3_′), 116.9, 118.5, 118.9 (OCHF_2_), 129.5 (C_2_′), 145.2 (C_1_′), 145.3 (C-2), 149.3 (C-8a), 150.0 (C_4_′), 166.6 (COOCH(CH_3_)_2_), 194.7 (C-5). HRMS (ESI/Q-TOF): *m/z* calculated for C_23_H_27_F_2_NO_4_ [M + H]+ found 420.2067.

Isobutyl 4-(4-(difluoromethoxy)phenyl)-2,7,7-trimethyl-5-oxo-1,4,5,6,7,8-hexahydroquinoline-3-carboxylate (**3d**): Yield: 70%; yellow solid; mp 165–166 °C; IR (ν, cm^−1^) 3196 (N-H stretching); 3074 (C-H stretching, aromatic); 2965 (C-H stretching, aliphatic); 1676 (C=O stretching, ester); 1645 (C=O stretching, ketone). ^1^H NMR (500 MHz, DMSO-*d_6_*, ppm): δ 0.80 (3H; *d*; *J* = 6.75 Hz; (OCH_2_CH(CH_3_)_2a_)), 0.81 (3H; *d*; *J* = 6.75 Hz; (OCH_2_CH(CH_3_)_2a_)), 0.81 (3H; *s*; 7-CH_3_), 1.00 (3H; *s*; 7-CH_3_), 1.77–1.82 (H; *m*; (OCH_2_CH(CH_3_)_2_)), 1.98 (1H; *d*; *J* = 16 Hz; quinoline H8a), 2.16 (1H; d; *J* = 16 Hz; quinoline H8b), 2.29 (3H; *s*; 2-CH_3_), 2.28 (1H; *d*; *J* = 16 Hz; quinoline H6a), 2.40 (1H; *d*; *J* = 16 Hz; quinoline H6b), 3.68–3.76 (2H; *m*; (OCH_2_CH(CH_3_)_2_)), 4.80 (H; *s*; quinoline H4), 6.99 (2H; *d*; *J* = 8.6 Hz; Ar-H3, Ar-H5), 7.12 (1H; *t*; *J* = 74.4 Hz; OCHF_2_), 7.18 (2H; *d*; *J* = 8.6 Hz; Ar-H2, Ar-H6), 9.12 (1H; *s*; NH). ^13^C NMR (125 MHz, DMSO-*d_6_*, ppm): δ 18.8 (2-CH_3_), 19.4 (COOCH_2_CH(CH_3_)_2_), 26.9 (7-CH_3_), 27.7 (COOCH_2_CH(CH_3_)_2_), 29.5 (C-7), 32.6 (C-8), 35.7 (C-4), 50.6 (C-6), 69.7 (COOCH_2_CH(CH_3_)_2_), 103.52 (C-3), 110.4 (C-4a), 114.8 (C_3_′), 116.8, 118.6, 118.9 (OCHF_2_), 129.5 (C_2_′), 145.0 (C_1_′), 146.0 (C-2), 149.3 (C-8a), 149.8 (C_4_′), 167.2 (COOCH_2_CH(CH_3_)_2_), 194.7 (C-5). HRMS (ESI/Q-TOF): *m/z* calculated for C_24_H_29_F_2_NO_4_ [M + H]+ found 434.2235.

Tert-butyl 4-(4-(difluoromethoxy)phenyl)-2,7,7-trimethyl-5-oxo-1,4,5,6,7,8-hexahydroquinoline-3-carboxylate (**3e**) [40]: Yield: 65%; yellow solid; mp 214–215 °C; IR (ν, cm^−1^) 3211 (N-H stretching); 3080 (C-H stretching, aromatic); 2968 (C-H stretching, aliphatic); 1697 (C=O stretching, ester); 1641 (C=O stretching, ketone). ^1^H NMR (500 MHz, DMSO-*d_6_*, ppm): δ 0.84 (3H; *s*; 7-CH_3_), 1.00 (3H; *s*; 7-CH_3_), 1.31 (9H; *s*; COOC(CH_3_)_3_), 1.97 (1H; *d*; *J* = 16 Hz; quinoline H8a), 2.14 (1H; *d*; *J* = 16 Hz; quinoline H8b), 2.25 (3H; *s*; 2-CH_3_), 2.28 (1H; *d*; *J* = 16.95 Hz; quinoline H6a), 2.39 (1H; *d*; *J* = 16.95 Hz; quinoline H6b), 4.78 (H; *s*; quinoline H4), 7.0 (2H; *d*; *J* = 9 Hz; Ar-H3, Ar-H5), 7.14 (1H; *t*; *J* = 74.4 Hz; OCHF_2_), 7.17 (2H; *d*; *J* = 9 Hz; Ar-H2, Ar-H6), 8.99 (1H; *s*; NH). ^13^C NMR (125 MHz, DMSO-*d_6_*, ppm): δ 18.7 (2-CH_3_), 27.0 (7-CH_3_), 28.3 (COOC(CH_3_)_3_), 29.4 (C-7), 32.0 (C-8), 36.2 (C-4), 50.6 (C-6), 79.2 (COOC(CH_3_)_3_), 105.4 (C-3), 110.0 (C-4a), 114.8 (C_3_′), 116.9, 118.4, 118.9 (OCHF_2_), 129.4 (C_2_′), 144.5 (C_1_′), 145.3 (C-2), 149.3 (C-8a), 150.0 (C_4_′), 166.7 (COOC(CH_3_)_3_), 194.6 (C-5). HRMS (ESI/Q-TOF): *m/z* calculated for C_24_H_29_F_2_NO_4_ [M + H]+ found 434.2328.

### 3.3. Biological activity

Cell proliferation and viability are important parameters for biological studies on drug candidates. Cell proliferation is defined as the cellular growth rate or the quantified value for the daughter cell population. Cell viability reflects the quantification of the number of live cells and is usually expressed as a percentage of the control. As an indicator of acute toxicity, the cytotoxic effect of a potential drug molecule should be analyzed before further activity tests. This fundamental information also constitutes the basis of investigating other biological effects, such as the modulation of inflammation. For this purpose, there are several dyes including neutral red, crystal violet, and trypan blue used to stain viable or dead cells. In addition, MTT, 2-(4-iodophenyl)-3-(4-nitrophenyl)-5-(2,4-(4-disulfophenyl)-2H-tetrazolium (WST), 3-(4,5-dimethylthiazol-2-yl)-5-(3-carboxymethoxyphenyl)-2-(4-sulfophenyl)-2H-tetrazolium (MTS), and 2,3-bis-(2-methoxy-4-nitro-5-sulfophenyl)-2H-tetrazolium-5-carboxyanilide (XTT) assays can be used to determine cell viability [45]. The MTT test provides reliable data and ease to measure cell viability by comparing percentages of cell viability between control and application groups.

**Figure 6:**
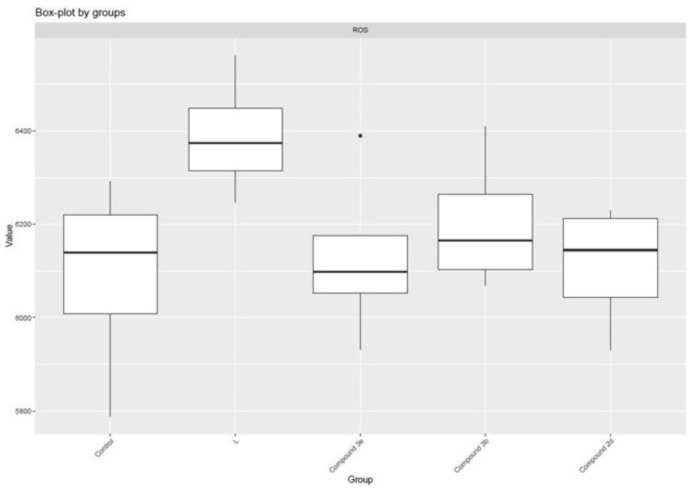
Reactive oxygen species in the study groups.

**Figure 7:**
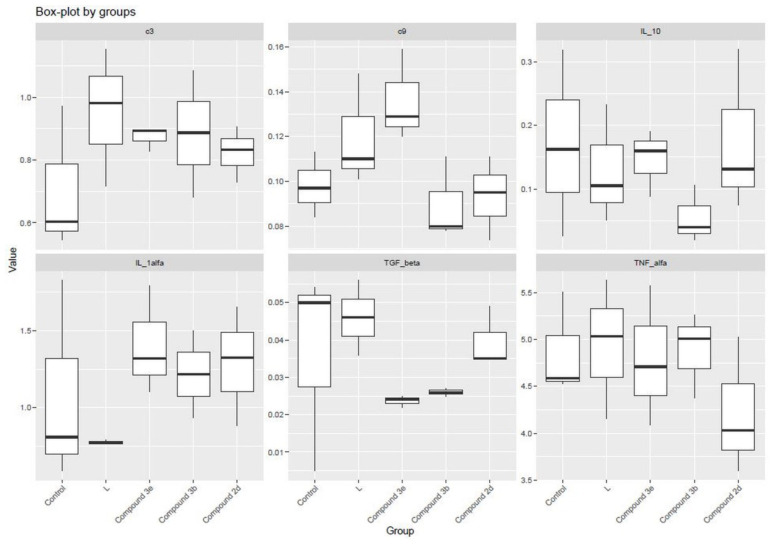
Inflammation markers and and complement protein levels in the study groups. IL-1α: interleukin-1-alpha; IL-10: interleukin-10; TGF-β1: transforming growth factor-1-beta; TNF-α: tumor necrosis factor-alpha; C3: complement C3; C9: complement C9.

Within the scope of biological activity studies, the cytotoxic properties of all synthesized compounds were tested by MTT method in the 3T3 cell line and these values are shown in Table 1. The 50% inhibitory concentration (IC_50_) and 30% inhibitory concentration (IC_30_) values of the compounds were determined. ROS levels are shown in Figure 6. The least cytotoxic compounds, namely **2d**, **3b**, and **3e**, were selected to determine their oxidative potential, inhibitory activity against inflammation mediators, and effects on complement system protein levels (Figure 7).

Procaspase-1 is recruited to the caspase recruitment domain (ASC) via a homotypic interaction of CARD domains facilitating caspase-1 activation [46]. The activation of proinflammatory protease caspase-1, an important component of the caspase pathway, occurs via recruitment to a multiprotein complex known as the inflammasome [47]. Following caspase-1-dependent processing of pro-IL-1β, mature IL-1β is rapidly secreted from the cell in response to inflammation. IL-1β, a potent proinflammatory cytokine, is a crucial factor for host-defense responses to infection and injury [48]. When compounds **2d**, **3b**, and **3e** were examined for their IL-1α inhibitory effects, it was found that although the compounds exerted antiinflammatory effects in LPS-induced cells, they did not provide any significant decrease compared to the control.

IL-10 is an antiinflammatory cytokine that is a member of the class-2 family of cytokines. IL-10 inhibits the induction of the secretion of proinflammatory cytokines TNF-α, IL-1β, IL-12, and interferon gamma (IFN-γ) by myeloid immune cells [49]. When the effects of these compounds on IL-10 levels were evaluated, no decrease was observed in comparison to the control.

TNF-α, an inflammatory cytokine, is responsible for a diverse range of intracellular signaling events. Such signaling may lead to necrosis or apoptosis. The protein is also important for resistance to infection and cancers [50]. When the effects of compounds **2d**, **3b**, and **3e** were evaluated, no decrease in TNF-α levels was observed.

In humans, the TGF-β family of growth factors controls several cellular pathways, which respond to the homeostasis of most human tissues. Several in vitro and in vivo studies have provided significant insight into the TGF-β signal transduction network, suggesting that this protein family inhibits cellular proliferation and provides tumor suppression. However, in neoplastic cells, the TGF-β family loses its antiproliferative effect and becomes an oncogenic factor. Disruption of the TGF-β pathway has been implicated in many human diseases, including solid and hematopoietic tumors [51]. When our results were evaluated, we observed that compounds **2d**, **3b**, and **3e** provided marked reduction in TGF-β1 levels compared to the control group and cells with only LPS applied. According to the biological activity results, compound **3e**, which has the structure of tert-butyl 2,7,7-trimethyl-4-(4-difluoromethoxyphenyl)-5-oxo-1,4,5,6,7,7,8-hexahydroquinoline-3-carboxylate, achieved decreases in the levels of inflammation markers. In addition to the two methyl substitutions at position 7 of the hexahydroquinoline ring, the ethyl structure in the ester group might have shown a positive effect on its antiinflammatory properties. Considering the main structure of compound **2d** and compound **3e**, it is thought that the bulky ester group at position 3 of the hexahydroquinoline ring has a positive contribution to the biological activity. Both compounds also carry iso and tert butyl groups. We can suggest that derivatives bearing a methyl substituent on the hexahydroquinoline ring’s 7th position have higher activity potential. Compound **3e** may have potential in reducing inflammation. However, more data are needed to establish a comprehensive structure–activity relationship. Mechanistic in vitro studies on other cell lines and in vivo research are needed to establish a better understanding of the biological activities of compound **3e**, particularly on the TGF-β pathway.

The complement system consists of several distinct plasma proteins. These proteins interact with one another in order to provide an immediate forceful response to pathogens even at first encounter and induce a series of inflammatory responses against infection. While complement proteins can provide a successful defense against microorganisms, they can also protect host cells from their attack in the process [52].

Complement protein C3 plays a crucial role in this process by acting as a cascade alert and playing a role in a point of convergence of activation pathways. Moreover, C3 can amplify the complement response as a direct effector and coordinator of downstream immune responses. Recent studies have shown that C3 not only fights against pathogens but also has roles in a variety of homeostatic processes including tissue regeneration, synapse pruning to clear debris, and tumor cell progression control. At the same time, its central position in immune surveillance makes C3 a target for microbial immune evasion and, if improperly engaged, a trigger point for various clinical conditions. Complement C3 levels are found to be increased in several pathological conditions, including diabetes, lymphoid cancers such as Hodgkin lymphoma, sarcomas, leukemia, diabetes, ischemic stroke, and ulcerative colitis. Concerning its crucial role in immunity, C3 is a therapeutic target and studies are ongoing to synthesize novel C3 inhibitors [53].

C9 is the last protein that binds to the assembling membrane attack complex (MAC) and this binding completes the series of events that finally leads to target membrane destruction. Even though erythrocyte lysis can occur without the presence of the enzymatic action of C9, it can increase the rate of hemolysis, which is a relatively slow and temperature-sensitive reaction. Multiple C9 molecules bind to C5b-8 in MAC, and the C9-to-C8 ratio determines the size of the transmembrane channels formed by C5b-9 [54].

This study has found that although compound **3e** caused a slight increase in complement C3, the change was not significant compared to the control. However, compound **3e** significantly increased complement C9 levels, suggesting that it can have beneficial effects in the formation of MAC. Compounds **3b** and **2d** led to insignificant decreases in C3 and C9 levels. This suggests that both can reduce the activation of the complement cascade and the assembly of MAC, and the biological importance of these results must be evaluated with further experiments.

### 3.4. Molecular docking studies

**Figure 8:**
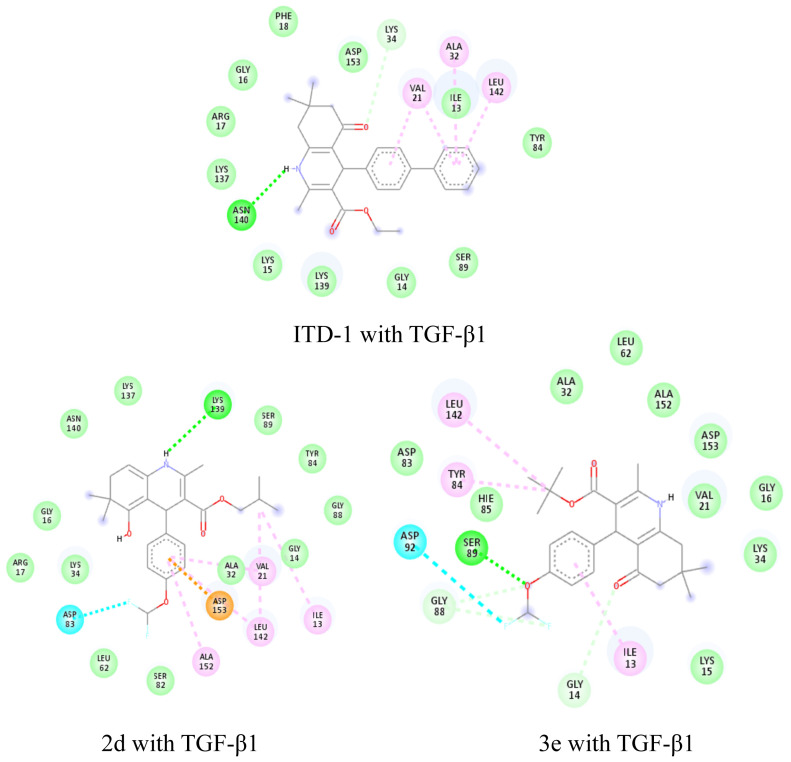
Interactions of compound **2d** and compound **3e** with the active TGF-β1 site of human TGF-β1 (PDB: 4X2F).

More data are needed to establish a comprehensive structure–activity relationship in terms of the number of compounds and the parameters examined within the scope of biological activity studies. In the molecular docking studies, the human TGF-β1 enzyme-enzyme inhibitor 4-amino-8-(4-aminophenyl)pyrido[2,3-d]pyrimidine-5(8*H*)-one compound complex (PDB: 4X2F) was used to investigate the placement of the molecules against the TGF-β1 enzyme in the enzyme active site and the interactions required for an effect. To carry out that investigation, selective and potent TGF-β1 enzyme inhibitors in the literature were screened. The compound ethyl 2,7,7-trimethyl-5-oxo-4-(4-phenylphenyl)-1,4,6,8-tetrahydroquinoline-3-carboxylate (ITD-1), the first selective TGF-β1 inhibitor (IC_50_ = 460 nm), was used as the reference compound for molecular docking studies and the binding energy in the enzyme active site (PDB: 4X2F) was calculated as −8.5 kcal/mol. When the interactions of the ITD-1 compound with the TGF-β1 enzyme were examined, it was observed that the compound exerted hydrogen bond interactions between the in-ring secondary nitrogen atom in the 1,4,6,8-tetrahydroquinoline ring with Asn140 and between the oxygen atom of the carbonyl group with Lys34, as well as hydrophobic interactions between the phenyl rings connected to the main ring at the 4th position with Val21, Ala32, and Leu142. Molecular modeling studies of the synthesized and characterized compounds were carried out, and the binding energies at the active site were calculated as −8.1 kcal/mol and −7.6 kcal/mol for compound **3e** and compound **2d**, respectively. In the active binding site of compound **2d**, hydrophobic interactions with Val21, Leu142, Ala152, Asp153, and Ile13; halogen bond interactions with Phe83; and hydrogen bonding with Lys139 were observed. Compound **3e** has better affinity for the molecular target in the protein structure. When the binding modes of these compounds in the active site of TGF-β1 were analyzed, it was found that compound **3e** had hydrophobic interactions with amino acids Leu142, Tyr84, and Ile13; halogen bond interactions with Asp92; and hydrogen bond interactions with Ser89, Gly88, and Gly14 in the active binding site. Hydrophobic interactions, hydrogen bonds, and halogen bonds are shown with pink, green, and cyan dashed lines, respectively, in Figure 8.

### 3.5. In silico studies

The parameters of the in silico studies of the molecules are shown in Table 2.

## 4. Conclusion

A drug candidate should not exert significant toxicity to a biological system and must be safe to use. Moreover, its effect on a target protein or pathway should be significant. In vitro tests are the first step to determine whether a drug candidate is safe for a biological system or not. Therefore, in this study, we first measured the cytotoxic potential of the synthesized drug candidates in 3T3 cells. After determining the least cytotoxic compounds, further experiments were carried out. Although none of the three selected compounds showed significant effects on IL-1α, IL-10, TNF-α, C3, or C9, all of the compounds provided a marked decrease in TGF-β levels. Since TGF-β signaling is involved in the pathogenesis and progression of various conditions, TGF-β inhibitors are suggested to be promising novel candidates for the treatment of muscular dystrophy, osteoporosis, fibrosis, and cancer. Therefore, further experiments could be carried out with these three compounds (**2d**, **3b**, and **3e**) to determine their possible TGF-β-inhibiting effects in other cell lines as well as in animals. Moreover, as compound **3e** (bearing bulky ester groups at position 3 and methyl groups at position 7 of the hexahydroquinoline ring) had the most significant effect on TGF-β, its mechanism of action on TGF-β signaling should be determined by pathway analysis. The findings were supported by molecular docking studies. Our future aim is to perform in vivo experiments on these three compounds, particularly on compound **3e**.

## Supplementary Information



## Figures and Tables

**Table 1:** Results of cytotoxicity experiments.

Compound	IC_30_ (mM)	IC_50_ (mM)
**1a**	47.80	105.98
**1b**	84.00	125.65
**1c**	70.98	114.12
**1d**	55.64	96.06
**1e**	37.80	83.56
**2a**	105.35	140.02
**2b**	35.81	85.11
**2c**	52.65	103.91
**2d**	57.93	98.49
**2e**	86.86	127.27
**3a**	104.23	150.75
**3b**	70.35	121.04
**3c**	91.77	131.03
**3d**	59.60	97.44
**3e**	40.90	90.65

MTT assay was performed to evaluate cytotoxicity.

Results were obtained from three different studies and the mean inhibitory concentrations were calculated.

IC_30_: inhibitory concentration 30; IC_50_: inhibitory concentration 50.

**Table 2:** In silico studies of compounds.

Compounds	MW	#RB	#HB acceptors	#HB donors	TPSA	iLOGP	ESOL Log S	GI absorption	Lipinski #violations	Veber #violations	Egan #violations	Muegge #violations
**1a**	363.36	5	6	1	64.63	2.97	−4.05	High	0	0	0	0
**1b**	311.37	4	3	1	55.40	2.88	−3.46	High	0	0	0	0
**1c**	325.40	4	3	1	55.40	3.05	−3.82	High	0	0	0	0
**1d**	339.43	5	3	1	55.40	3.25	−4.17	High	0	0	0	0
**1e**	339.43	4	3	1	55.40	3.22	−4.01	High	0	0	0	0
**2a**	391.41	5	6	1	64.63	3.25	−4.80	High	0	0	0	0
**2b**	405.44	6	6	1	64.63	3.57	−5.04	High	0	0	0	0
**2c**	419.46	6	6	1	64.63	3.65	−5.40	High	0	0	0	1
**2d**	433.49	7	6	1	64.63	3.61	−5.75	High	0	0	0	1
**2e**	433.49	6	6	1	64.63	3.72	−5.59	High	0	0	0	1
**3a**	391.41	5	6	1	64.63	3.29	−4.74	High	0	0	0	0
**3b**	405.44	6	6	1	64.63	3.48	−4.98	High	0	0	0	0
**3c**	419.46	6	6	1	64.63	3.68	−5.34	High	0	0	0	0
**3d**	433.49	7	6	1	64.63	3.92	−5.69	High	0	0	0	1
**3e**	433.49	6	6	1	64.63	3.85	−5.53	High	0	0	0	1

MW: Molecular weight, RB: Rotatable bond, HB: Hydrogen bond.

## References

[1] Nathan C Nonresolving inflammation redux Immunity 2022 55 4 592 605 10.1016/j.immuni.2022.03.016 35417674 PMC9003810

[2] Chen L Deng H Cui H Fang J Zuo Z Inflammatory responses and inflammation-associated diseases in organs Oncotarget 2018 9 6 7204 7218 10.18632/oncotarget.23208 29467962 PMC5805548

[3] Rocha DARF Morais NDS Priore SE Franceschini SDCC Inflammatory biomarkers and components of metabolic syndrome in adolescents: a systematic review Inflammation 2022 45 14 30 10.1007/s10753-021-01549-1 34546513

[4] Michels N Aart CV Morisse J Mullee A Huybrechts I Chronic inflammation towards cancer incidence: a systematic review and meta-analysis of epidemiological studies Critical Reviews in Oncology/Hematology 2021 157 103177 10.1016/j.critrevonc.2020.103177 33264718

[5] Meizlish ML Franklin RA Zhou X Medzhitov R Tissue homeostasis and inflammation Annual Review of Immunology 2021 39 557 581 10.1146/annurev-immunol-061020-053734 33651964

[6] Aguilar-Cazares D Chavez-Dominguez R Carlos-Reyes A Lopez-Camarillo C Hernadez de la Cruz ON Contribution of angiogenesis to inflammation and cancer Frontiers in Oncology 2019 9 1399 https://doi.org/10.3389%2Ffonc.2019.01399 31921656 10.3389/fonc.2019.01399 PMC6920210

[7] Singh N Baby D Rajguru JP Patil PB Thakkannavar SS Inflammation and cancer Annals of African Medicine 2019 18 3 121 10.4103/aam.aam_56_18 31417011 PMC6704802

[8] Hirano T IL-6 in inflammation, autoimmunity and cancer International Immunology 2021 33 3 127 148 10.1093/intimm/dxaa078 33337480 PMC7799025

[9] Riley JS Tait SW Mitochondrial DNA in inflammation and immunity EMBO Reports 2020 21 4 e49799 10.15252/embr.201949799 32202065 PMC7132203

[10] Chou TC Yang SP Pei D Amlodipine inhibits pro-inflammatory cytokines and free radical production and inducible nitric oxide synthase expression in lipopolysaccharide/interferon-γ-stimulated cultured vascular smooth muscle cells Japanese Journal of Pharmacology 2002 89 2 157 63 10.1254/jjp.89.157 12120758

[11] Salomon BL Leclerc M Tosello J Ronin E Piaggio E Tumor necrosis factor α and regulatory T cells in oncoimmunology Frontiers in Immunology 2018 9 444 10.3389/fimmu.2018.00444 29593717 PMC5857565

[12] Loo GV Bertrand MJ Death by TNF: a road to inflammation Nature Reviews Immunology 2023 23 5 289 303 https://doi.org/10.1038%2Fs41577-022-00792-3 10.1038/s41577-022-00792-3 PMC9665039 36380021

[13] Tanaka T Narazaki M Kishimoto T IL-6 in inflammation, immunity, and disease Cold Spring Harbor Perspectives in Biology 2014 6 10 a016295 10.1101/cshperspect.a016295 25190079 PMC4176007

[14] Kang S Narazaki M Metwally H Kishimoto T Historical overview of the interleukin-6 family cytokine Journal of Experimental Medicine 2020 217 5 e20190347 10.1084/jem.20190347 32267936 PMC7201933

[15] Choy EH Benedetti FD Takeuchi T Hashizume M John MR Translating IL-6 biology into effective treatments Nature Reviews Rheumatology 2020 16 6 335 345 10.1038/s41584-020-0419-z 32327746 PMC7178926

[16] Wooff Y Man SM Aggio-Bruce R Natoli R Fernando N IL-1 family members mediate cell death, inflammation and angiogenesis in retinal degenerative diseases Frontiers in Immunology 2019 10 1618 https://doi.org/10.3389%2Ffimmu.2019.01618 31379825 10.3389/fimmu.2019.01618 PMC6646526

[17] Wu YD Zhou B TNF-α/NF-κB/Snail pathway in cancer cell migration and invasion British Journal of Cancer 2010 102 4 639 644 https://doi.org/10.1038%2Fsj.bjc.6605530 20087353 10.1038/sj.bjc.6605530 PMC2837572

[18] Sohal HS A review on recent trends in synthesis and applications of 1, 4-dihydropyridines Materials Today: Proceedings 2022 48 1163 1170 10.1016/j.matpr.2021.08.209

[19] Pansuriya K Lalpara JN Hadiyal SD Dhaduk BB Dubal G Phenylboronic acid catalyzed synthesis of polysubstituted 1,4-dihydropyridine derivatives as promising antioxidant agents correlated with molecular docking Chemical Data Collections 2022 42 100946 10.1016/j.cdc.2022.100946

[20] Abdelwahab RE Darweesh AF Ragheb MA Abdelhamid IA Elwahy AH Synthesis of New 2-(4-(1, 4-Dihydropyridin-4-yl) Phenoxy)-N-Arylacetamides and Their Heterocyclic-Fused Derivatives via Hantzsch-Like Reaction Polycyclic Aromatic Compounds 2023 43 3 1974 1986 10.1080/10406638.2022.2039240

[21] Fadda AA Berghot MA Amer FA Badawy DS Bayoumy NM Synthesis and antioxidant and antitumor activity of novel pyridine, chromene, thiophene and thiazole derivatives Archiv der Pharmazie 2012 345 5 378 385 10.1002/ardp.201100335 22189501

[22] Velena A Zarkovic N Gall Troselj K Bisenieks E Krauze A 1,4-dihydropyridine derivatives: dihydronicotinamide analogues—model compounds targeting oxidative stress Oxidative Medicine and Cellular Longevity 2016 1 1892412 10.1155/2016/1892412 PMC4736762 26881016

[23] Choe SH Choi EY Hyeon JY Keum BR Choi IS Effect of nifedipine, a calcium channel blocker, on the generation of nitric oxide and interleukin-1β by murine macrophages activated by lipopolysaccharide from *Prevotella intermedia* Naunyn-Schmiedeberg’s Archives of Pharmacology 2021 394 59 71 10.1007/s00210-020-01958-3 32780228

[24] Facchin BM Lubschinski TL Moon YJK Fragoso de Oliveira PG Beck BK Evaluation of the anti-inflammatory effect of 1, 4-dihydropyridine derivatives Fundamental & Clinical Pharmacology 2023 38 1 168 182 10.1111/fcp.12945 37558213

[25] Matsumori A Nunokawa Y Sasayama S Nifedipine inhibits activation of transcription factor NF-κB Life Sciences 2000 67 21 2655 2661 10.1016/s0024-3205(00)00849-3 11104367

[26] Komoda H Inoue T Node K Anti-inflammatory properties of azelnidipine, a dihydropyridine-based calcium channel blocker Clinical and Experimental Hypertension 2010 32 2 121 128 10.3109/10641960903254414 20374186

[27] Li XQ Cao W Li T Zeng AG Hao LL Amlodipine inhibits TNF-α production and attenuates cardiac dysfunction induced by lipopolysaccharide involving PI3K/Akt pathway International Immunopharmacology 2009 9 9 1032 1041 10.1016/j.intimp.2009.04.010 19393774

[28] Shalaby MA Fahim AM Rizk SA Microwave-assisted synthesis, antioxidant activity, docking simulation, and DFT analysis of different heterocyclic compounds Scientific Reports 2023 13 1 4999 10.1038/s41598-023-31995-w 36973332 PMC10042854

[29] Faizan M Kumar R Mazumder A Salahuddin Kukreti N Hantzsch reaction: The important key for pyridine/dihydropyridine synthesis Synthetic Communications 2024 54 15 1221 1244 10.1080/00397911.2024.2377738

[30] Chu XM Wang C Liu W Liang LL Gong KK Quinoline and quinolone dimers and their biological activities: an overview European Journal of Medicinal Chemistry 2019 161 101 117 10.1016/j.ejmech.2018.10.035 30343191

[31] Devi L Robert AR Ganja H Maddila S Jonnalagadda SB A rapid, sustainable and environmental friendly protocol for the catalyst-free synthesis of 2-methyl-5-oxo-hexahydroquinoline-3-carboxylate via ultrasonic irradiation Chemical Data Collections 2020 28 100432 10.1016/j.cdc.2020.100432

[32] Ranjbar S Edraki N Firuzi O Khoshneviszadeh M Miri R 5-Oxo-hexahydroquinoline: an attractive scaffold with diverse biological activities Molecular Diversity 2019 23 2 471 508 10.1007/s11030-018-9886-4 30390186

[33] Bladen C Gadotti VM Gündüz MG Berger ND Şimşek R 1,4-Dihydropyridine derivatives with T-type calcium channel blocking activity attenuate inflammatory and neuropathic pain Pflügers Archiv-European Journal of Physiology 2015 467 1237 1247 10.1007/s00424-014-1566-3 24990197

[34] Rose U Draeger M Synthesis, configuration, and calcium modulatory properties of enantiomerically pure 5-oxo-1,4,5,6,7,8-hexahydroquinoline-3-carboxylates Journal of Medicinal Chemistry 1992 35 12 2238 2243 10.1021/jm00090a014 1319494

[35] Ranjbar S Khoshneviszadeh M Tavakkoli M Miri R Edraki N 5-Oxo-hexahydroquinoline and 5-oxo-tetrahydrocyclopentapyridine derivatives as promising antiproliferative agents with potential apoptosis-inducing capacity Molecular Diversity 2022 26 3 1481 1500 10.1007/s11030-021-10281-9 34671894

[36] Zahedi M Asgari Q Badakhshan F Sakhteman A Ranjbar S Anti-Toxoplasma gondii activity of 5-oxo-hexahydroquinoline derivatives: synthesis, in vitro and in vivo evaluations, and molecular docking analysis Research in Pharmaceutical Sciences 2020 15 4 367 380 https://doi.org/10.4103%2F1735-5362.293515 33312215 10.4103/1735-5362.293515 PMC7714012

[37] Teraiya N Karki SS Chauhan A Synthesis, cytotoxicity evaluation and molecular docking of fluorine containing hexahydroquinoline-3-carbonitrile derivatives Current Drug Discovery Technologies 2022 19 1 54 64 10.2174/1570163817666201229154848 33372877

[38] Kirk KL Fluorine in medicinal chemistry: recent therapeutic applications of fluorinated small molecules Journal of Fluorine Chemistry 2006 127 8 1013 1029 10.1016/j.jfluchem.2006.06.007

[39] Wang J Sánchez-Roselló M Aceña JL Pozo CD Sorochinsky AE Fluorine in pharmaceutical industry: fluorine-containing drugs introduced to the market in the last decade (2001–2011) Chemical Reviews 2014 114 4 2432 2506 10.1021/cr4002879 24299176

[40] Gillis EP Eastman KJ Hill MD Donnelly DJ Meanwell NA Applications of fluorine in medicinal chemistry Journal of Medicinal Chemistry 2015 58 21 8315 8359 10.1021/acs.jmedchem.5b00258 26200936

[41] Yıldırım SÖ Akkurt M Çetin G Şimşek R Butcher RJ Synthesis, characterization, crystal structure and Hirshfeld surface analysis of isobutyl 4-[4-(difluoromethoxy) phenyl]-2, 6, 6-trimethyl-5-oxo-1, 4, 5, 6, 7, 8-hexahydroquinoline-3-carboxylate Acta Crystallographica Section E: Crystallographic Communications 2023 79 12 1132 1136 https://doi.org/10.1107%2FS2056989023009623 38313124 10.1107/S2056989023009623 PMC10833413

[42] Pehlivanlar E Yıldırım SÖ Şimşek R Akkurt M Butcher RJ Synthesis, crystal structure and Hirshfeld surface analysis of tert-butyl 4-[4-(difluoromethoxy) phenyl]-2, 7, 7-trimethyl-5-oxo-1, 4, 5, 6, 7, 8-hexahydroquinoline-3-carboxylate Acta Crystallographica Section E: Crystallographic Communications 2023 79 7 664 668 10.1107/S2056989023005455 37601569 PMC10439425

[43] Li XJ Huang FZ Wan Y Li YS Zhang WK Lipopolysaccharide stimulated the migration of NIH3T3 cells through a positive feedback between β-catenin and COX-2 Frontiers in Pharmacology 2018 9 1487 10.3389/fphar.2018.01487 30618773 PMC6305731

[44] Trott O Olson AJ AutoDock Vina: improving the speed and accuracy of docking with a new scoring function, efficient optimization, and multithreading Journal of Computational Chemistry 2010 31 2 455 461 10.1002/jcc.21334 19499576 PMC3041641

[45] Mani S Swargiary G In vitro cytotoxicity analysis: MTT/XTT, trypan blue exclusion Kalyuzhny AE Animal Cell Culture: Principles and Practice Cham Springer International Publishing 2023 267 284 10.1007/978-3-031-19485-6_18

[46] Lopez-Castejon G Brough D Understanding the mechanism of IL-1β secretion Cytokine Growth Factor Reviews 2011 22 4 189 195 10.1016/j.cytogfr.2011.10.001 22019906 PMC3714593

[47] Thornberry NA Bull HG Calaycay JR Chapman KT Howard AD A novel heterodimeric cysteine protease is required for interleukin-1 beta processing in monocytes Nature 1992 356 6372 768 774 10.1038/356768a0 1574116

[48] Dinarello CA Biologic basis for interleukin-1 in disease Blood 1996 87 6 2095 2147 10.1182/blood.V87.6.2095.bloodjournal8762095 8630372

[49] Loonen AJM Putative role of immune reactions in the mechanism of tardive dyskinesia Brain, Behavior & Immunity Health 2023 33 100687 10.1016/j.bbih.2023.100687 PMC10550815 37810262

[50] Idriss HT Naismith JH TNFa and the TNF receptor superfamily: structure-function relationship(s) Microscopy Research and Technique 2000 50 3 184 95 10.1002/1097-0029(20000801)50:3<184::AID-JEMT2>3.0.CO;2-H 10891884

[51] Kubiczkova L Sedlarikova L Hajek R Sevcikova S TGF-β - an excellent servant but a bad master Journal of Translational Medicine 2012 10 1 24 10.1186/1479-5876-10-183 22943793 PMC3494542

[52] Ricklin D Reis ES Mastellos DC Gros P Lambris JD Complement component C3–The “Swiss Army Knife” of innate immunity and host defense Immunological Reviews 2016 274 1 33 58 10.1111/imr.12500 27782325 PMC5427221

[53] Yang P Zhu Z Zang Y Bu X Xu T Increased serum complement C3 levels are associated with adverse clinical outcomes after ischemic stroke Stroke 2021 52 3 868 877 10.1161/STROKEAHA.120.031715 33517703

[54] Dudkina NV Spicer BA Reboul CF Conroy PJ Lukoyanova N Structure of the poly-C9 component of the complement membrane attack complex Nature Communications 2016 7 1 10588 10.1038/ncomms10588 PMC4742998 26841934

