# Supplementary material for: Synthesis and characterization of new hexahydroquinoline derivatives and evaluation of their cytotoxicity, intracellular ROS production, and inhibitory effects on inflammatory mediators
Source: Turk J Chem. 2024 Jul 23;48(4):659–75. doi: 10.55730/1300-0527.3686 (PMC11407359; doi:10.55730/1300-0527.3686)

**Synthesis and Characterization of New Hexahydroquinoline Derivatives, Evaluation of Their Cytotoxicity, Intracellular ROS Production and Inhibitory Effects on Inflammatory Mediators**

IR spectrum of compound **1a**


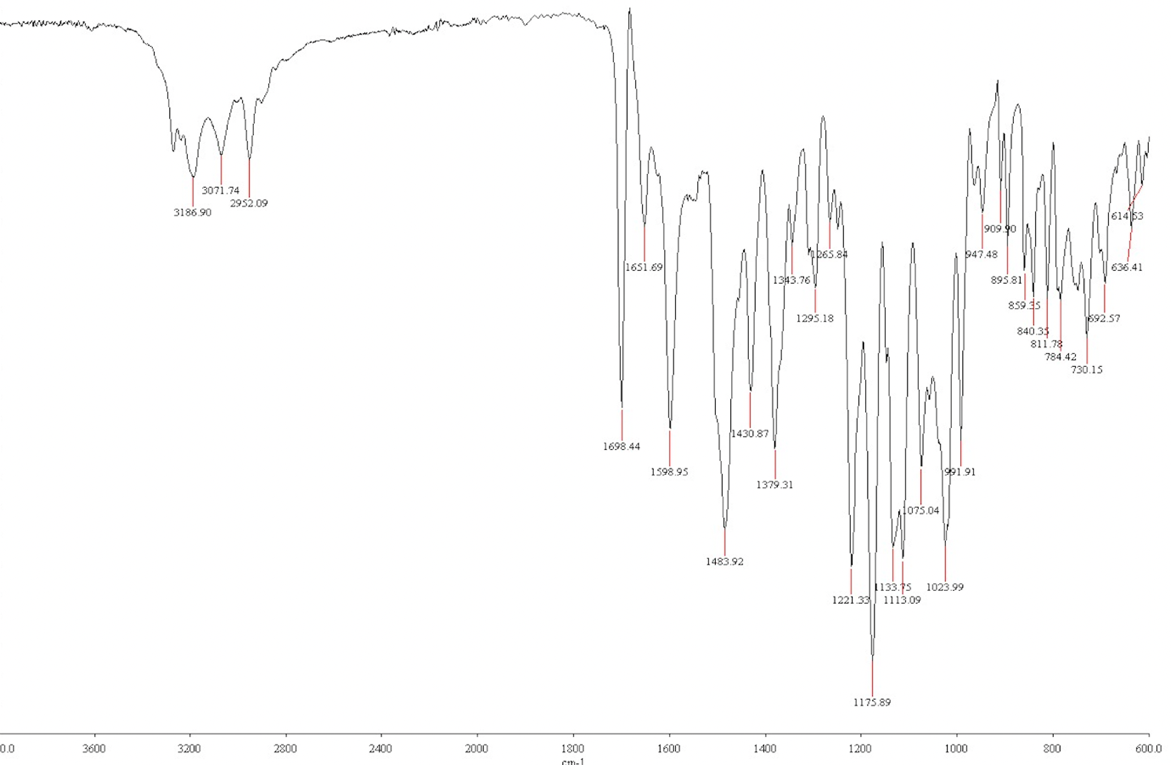


^1^H NMR spectrum of compound **1a**


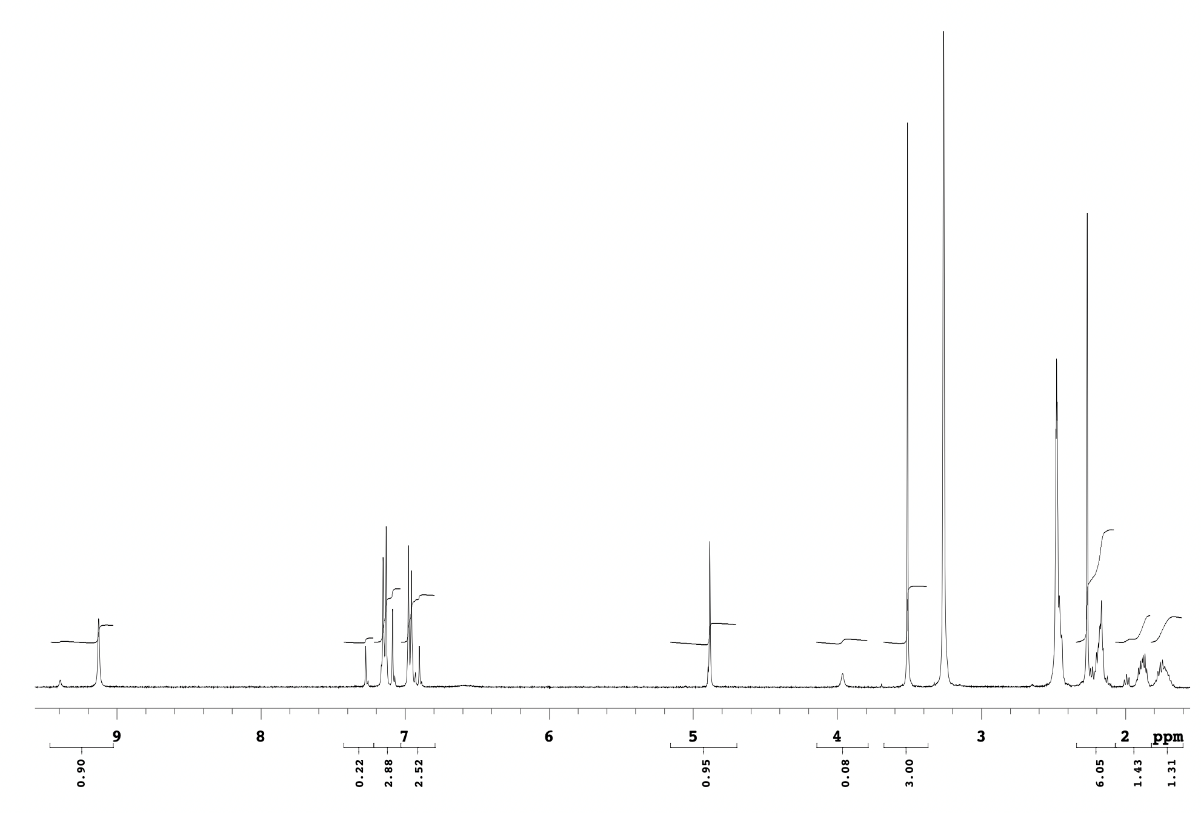

^13^C NMR spectrum of compound **1a**


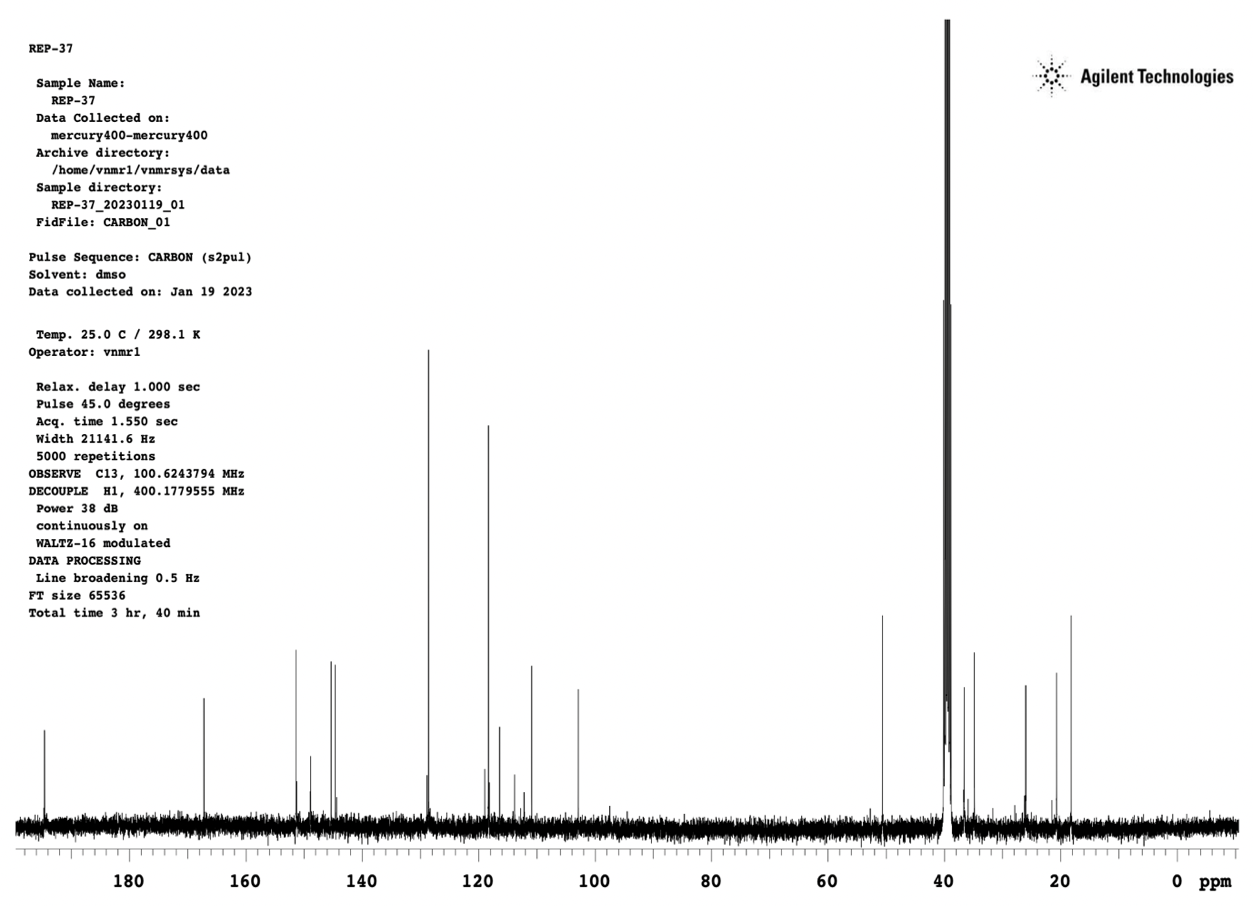

Mass-spectrum of compound **1a**


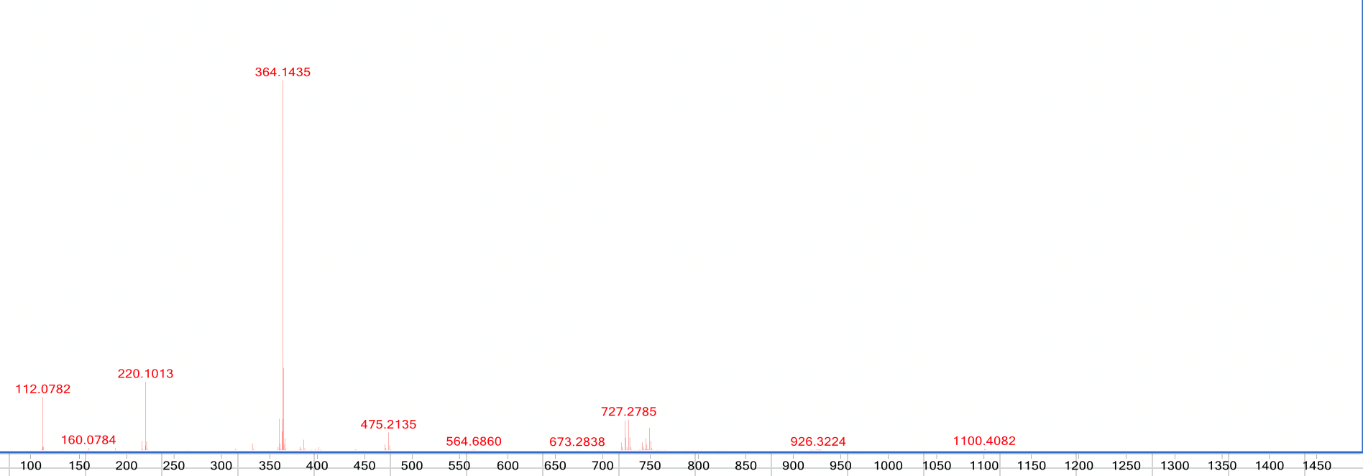


IR spectrum of compound **1b**


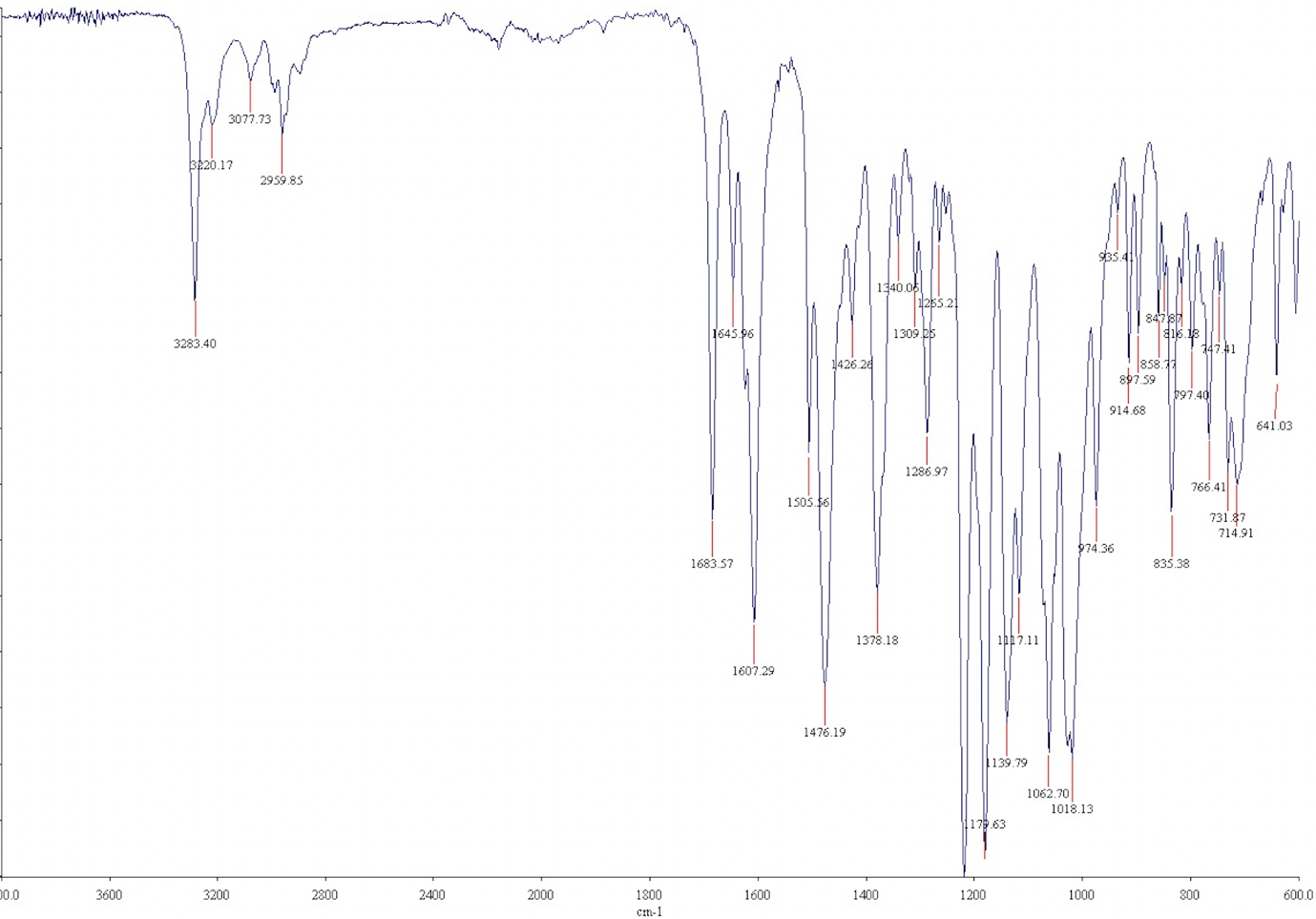


^1^H NMR spectrum of compound **1b**


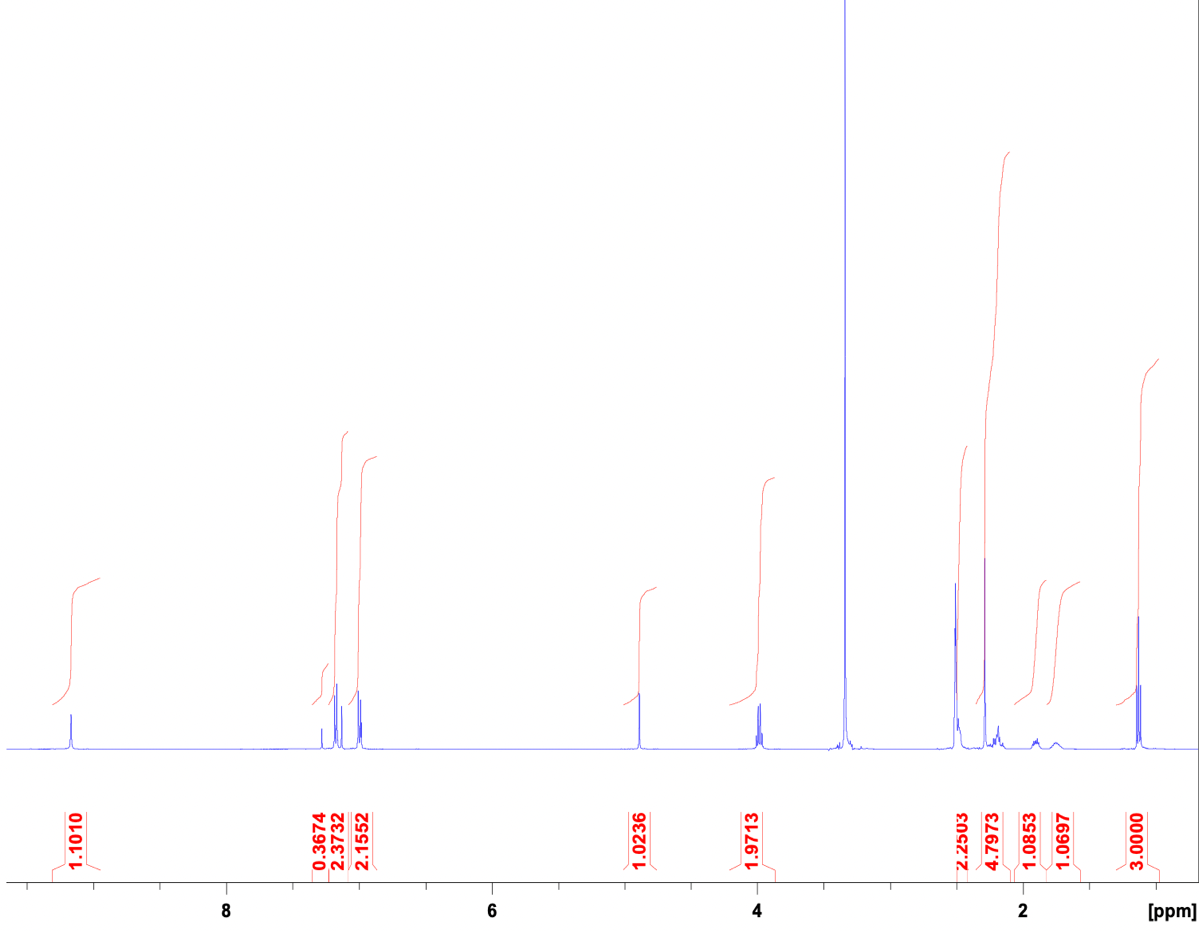

^13^C NMR spectrum of compound **1b**


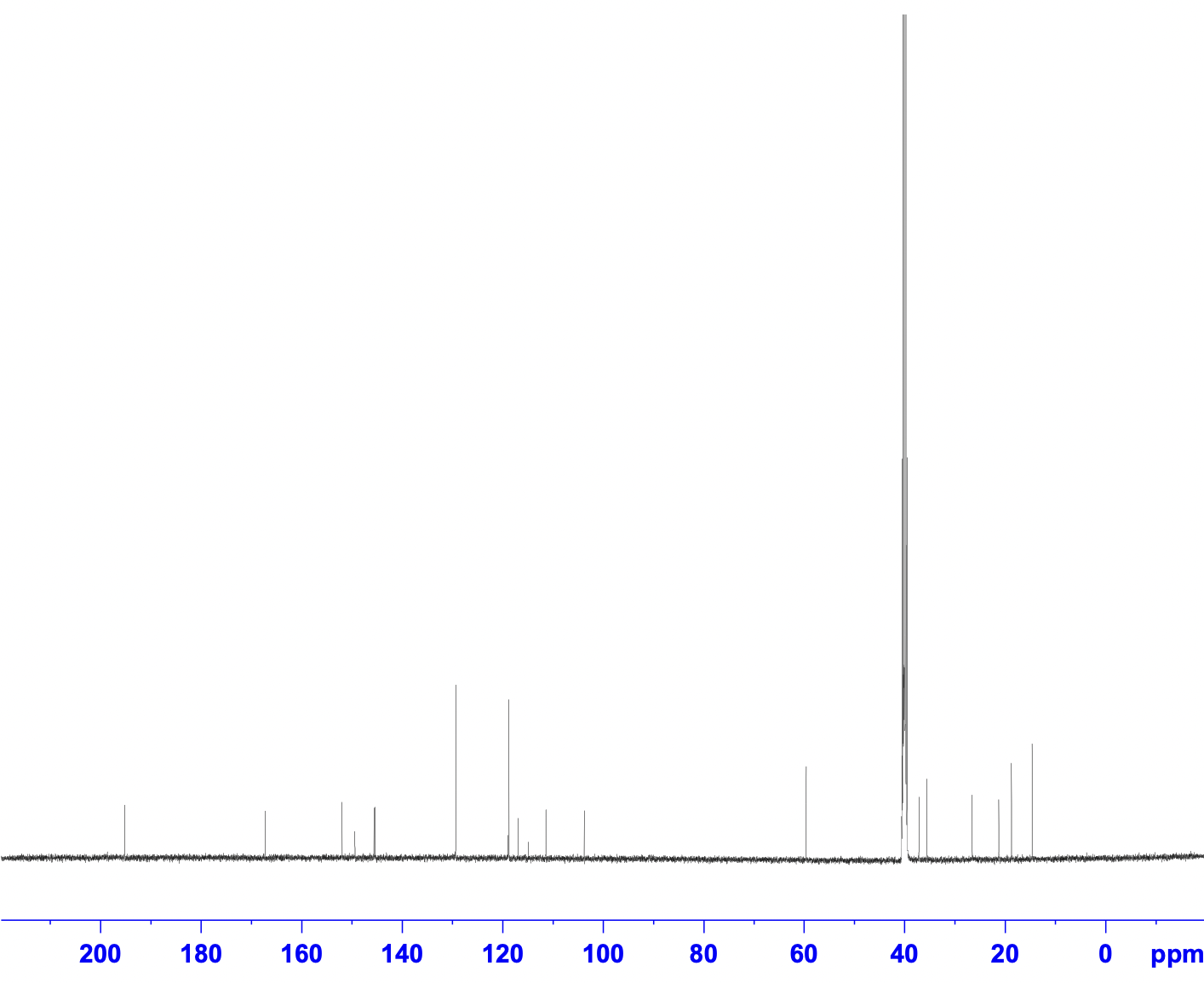

Mass-spectrum of compound **1b**


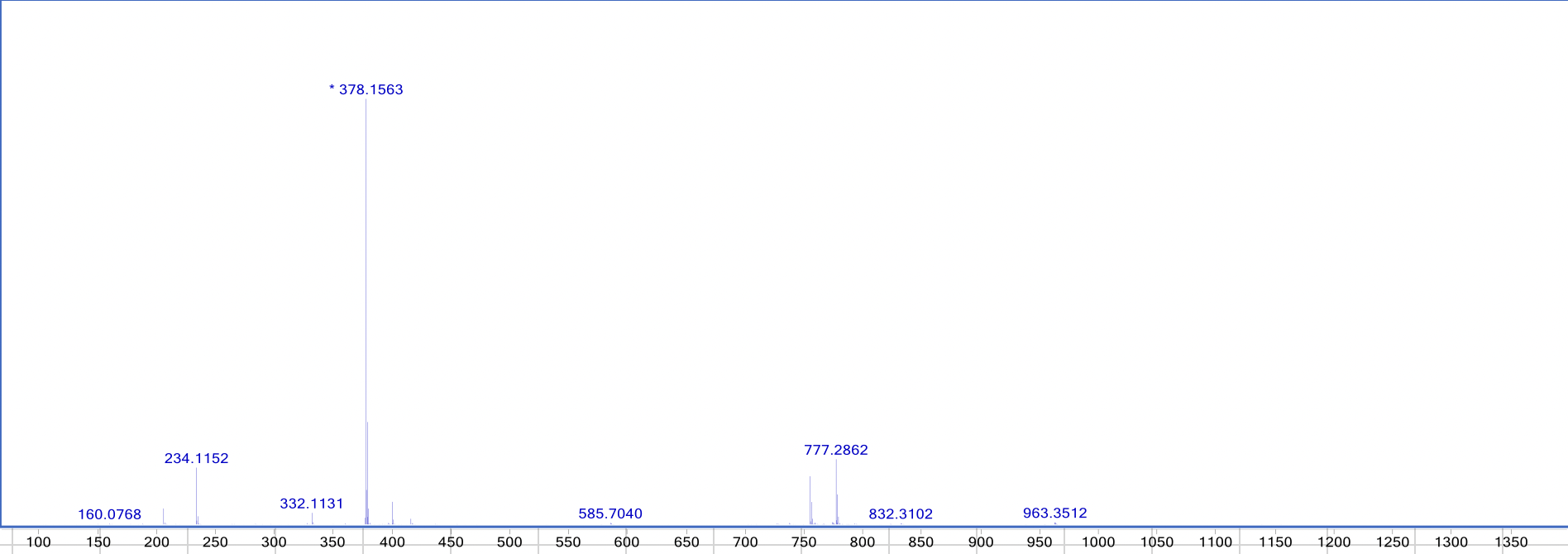


IR spectrum of compound **1c**


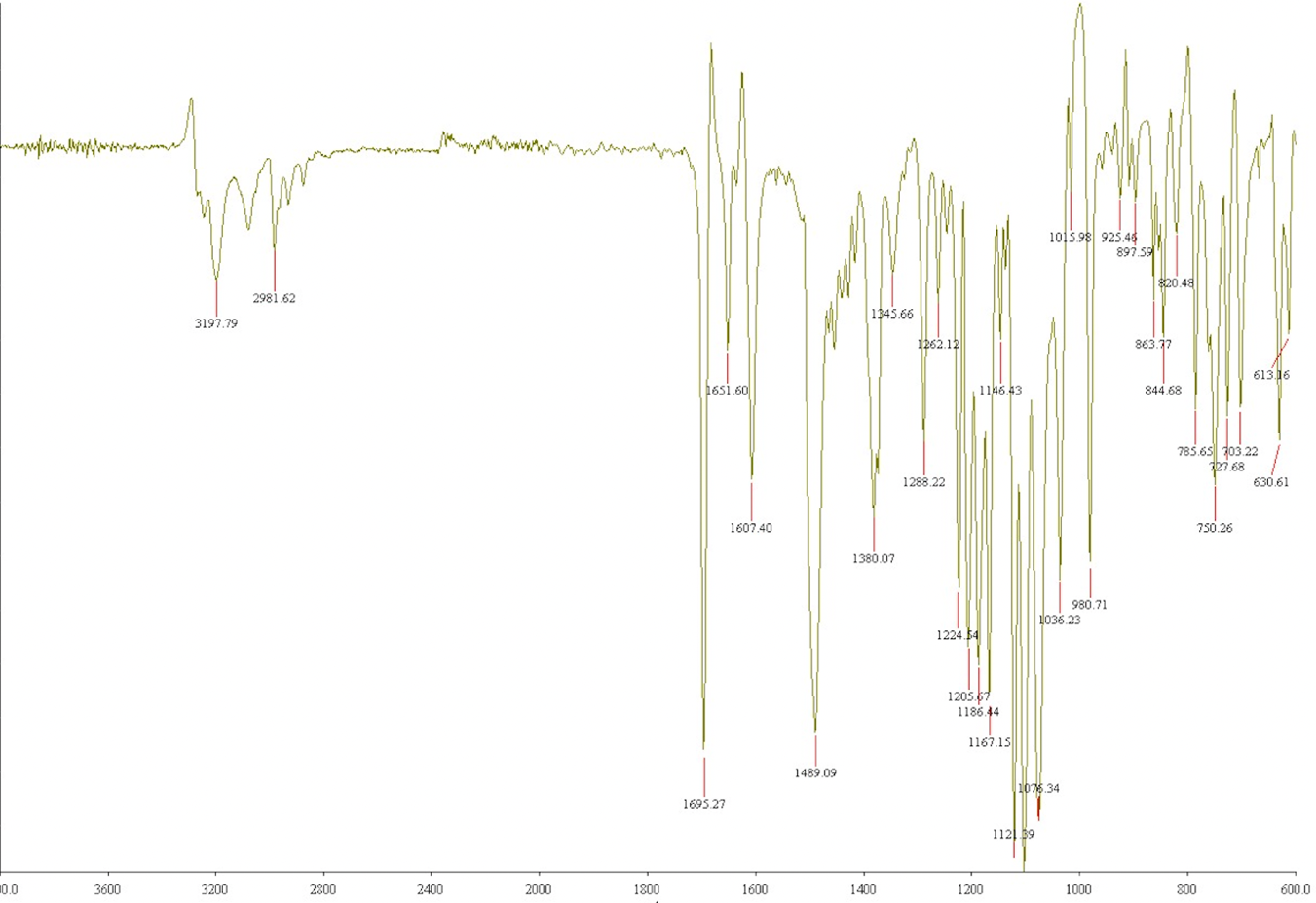


^1^H NMR spectrum of compound **1c**

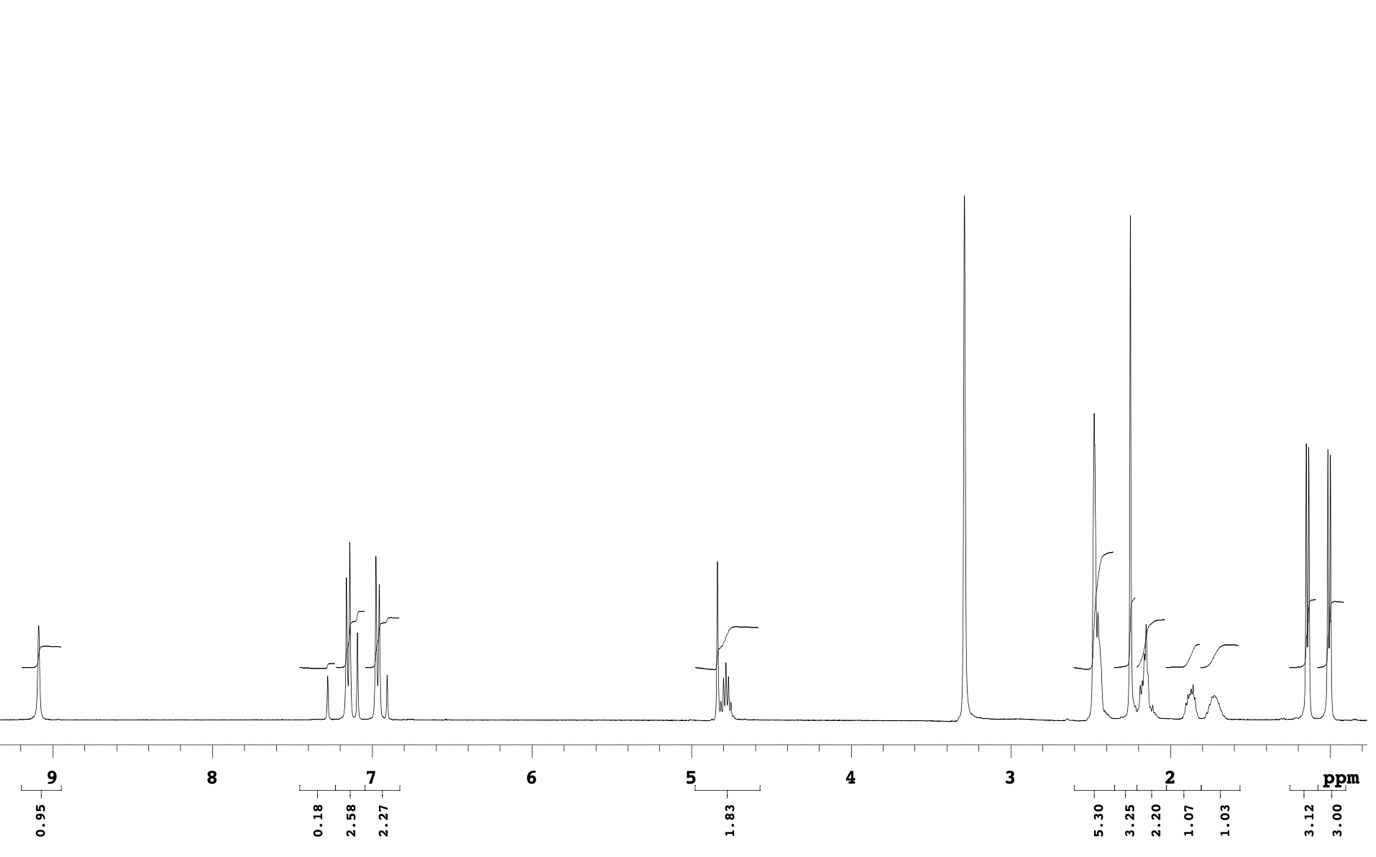


^13^C NMR spectrum of compound **1c**


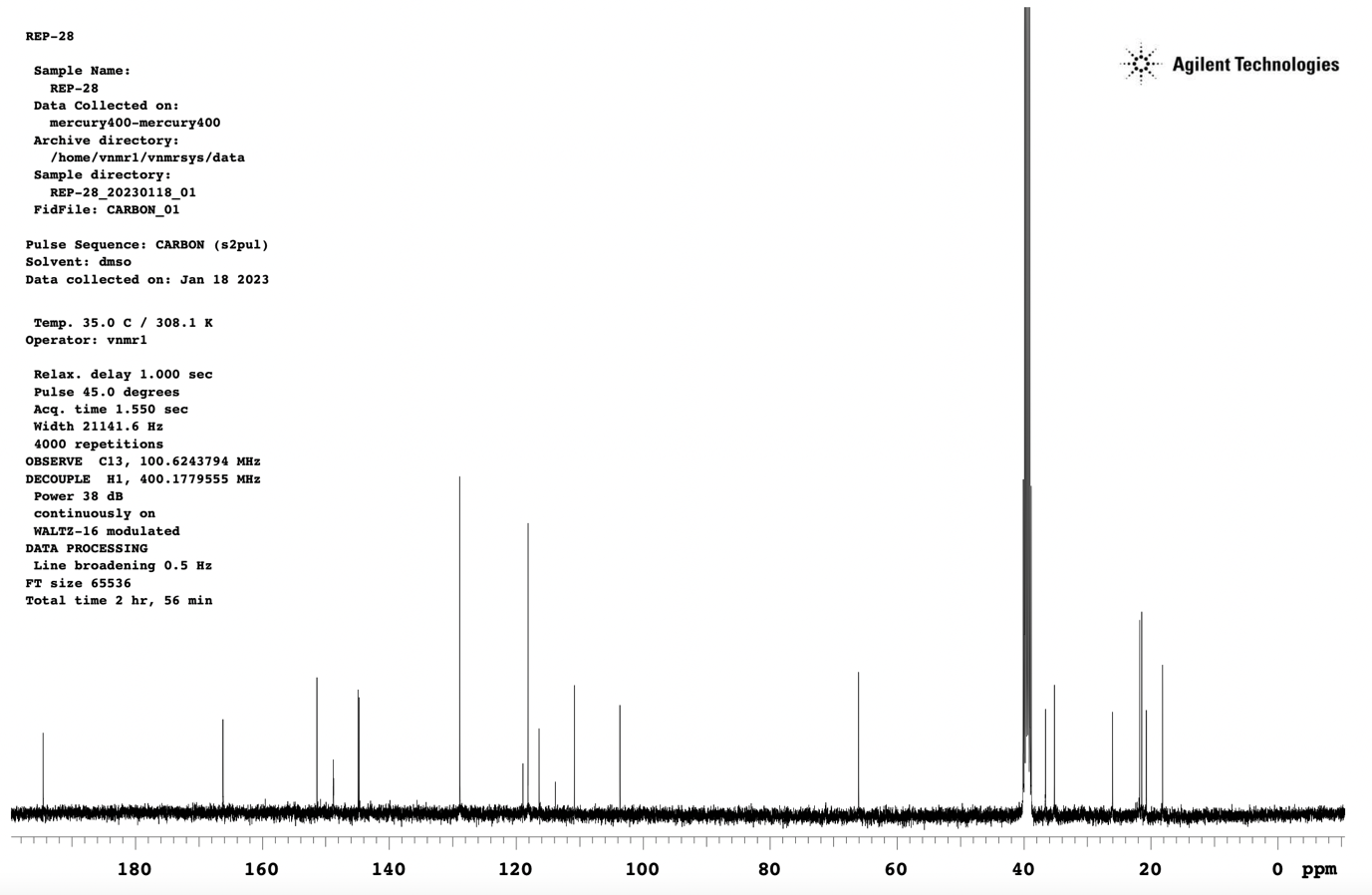

Mass-spectrum of compound **1c**


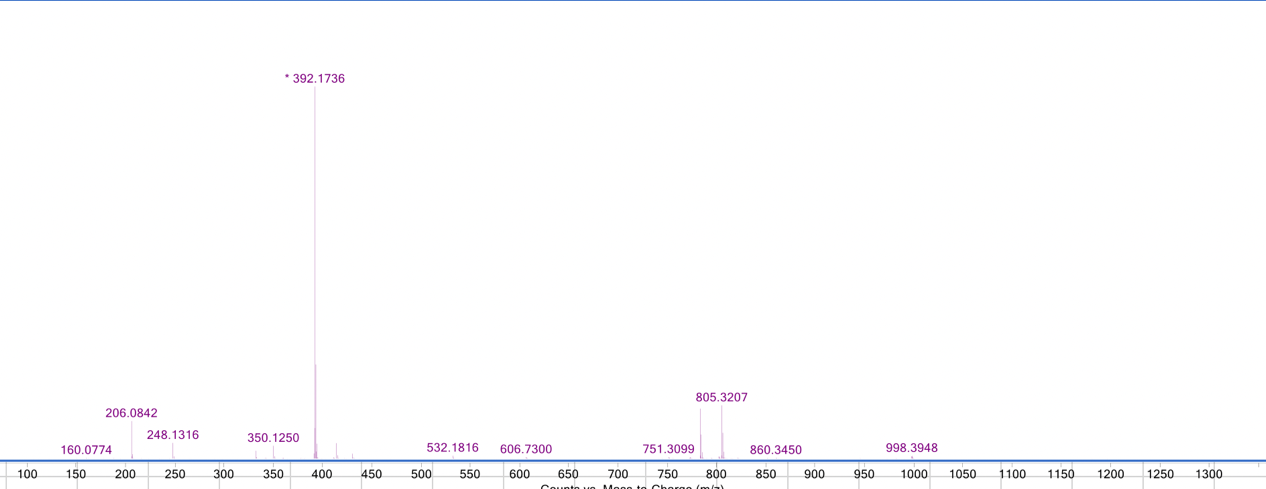


IR spectrum of compound **1d**


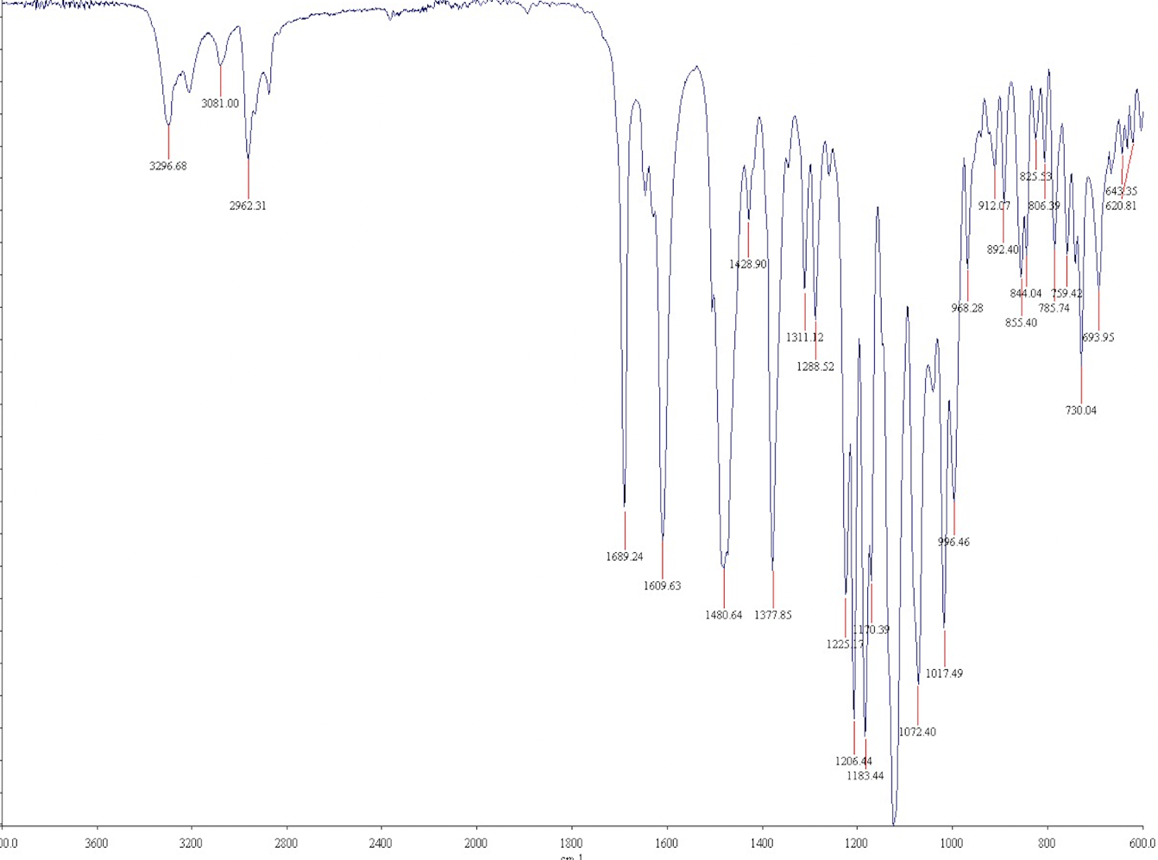


^1^H NMR spectrum of compound **1d**


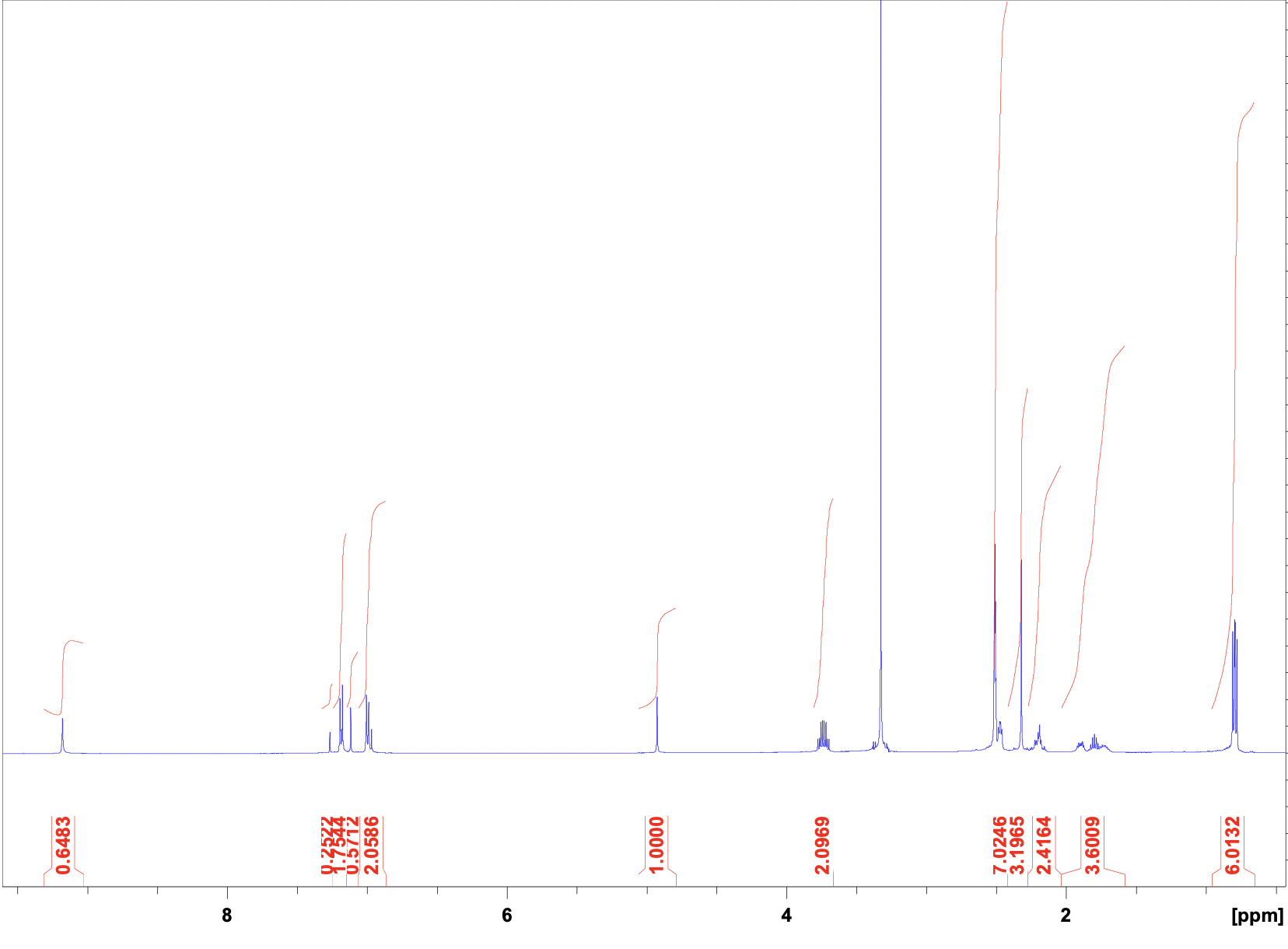

^13^C NMR spectrum of compound **1d**


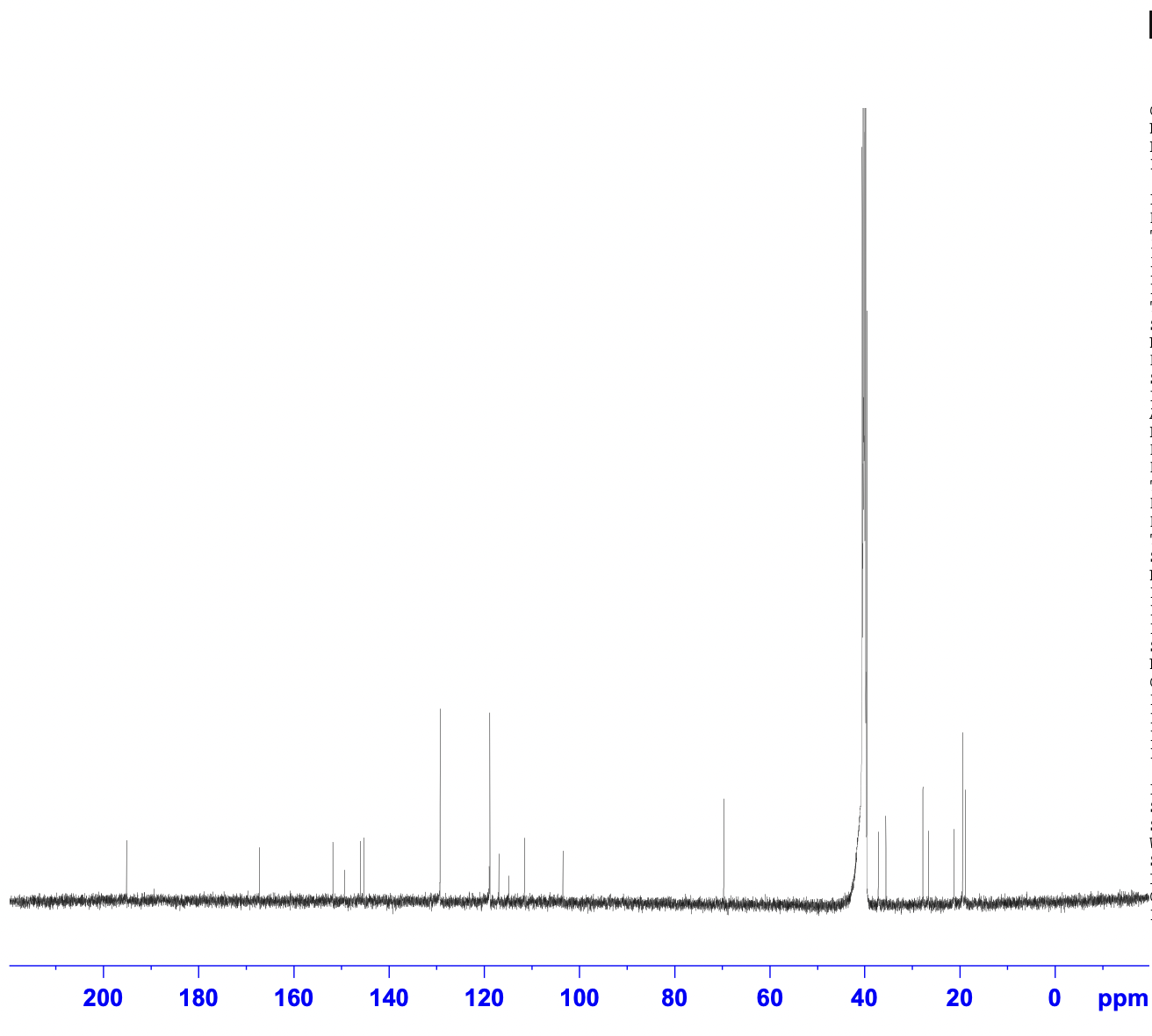

Mass-spectrum of compound **1d**


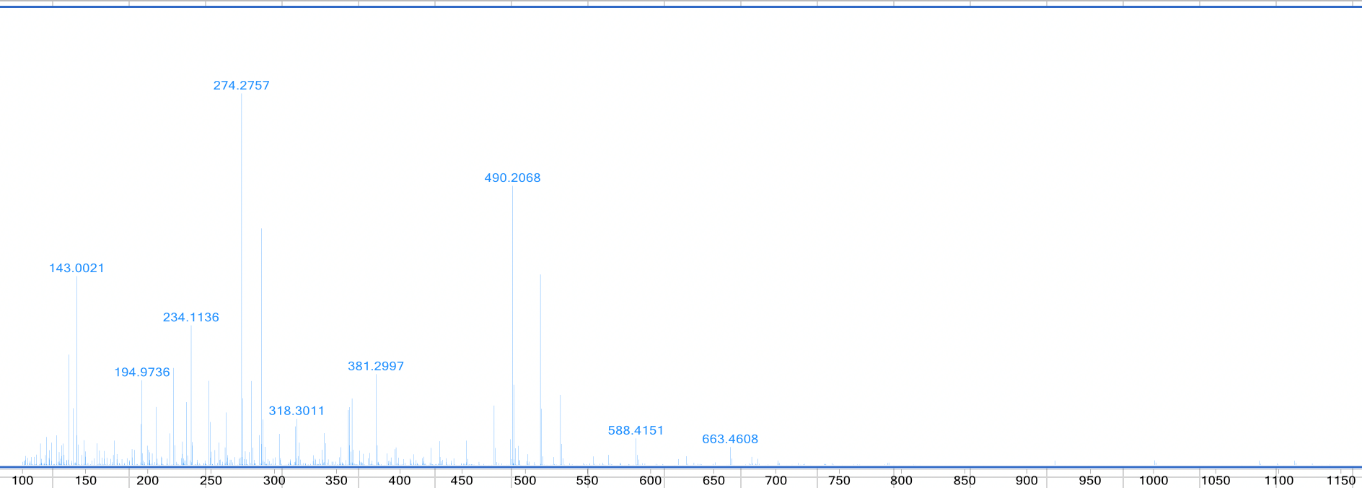


IR spectrum of compound **1e**


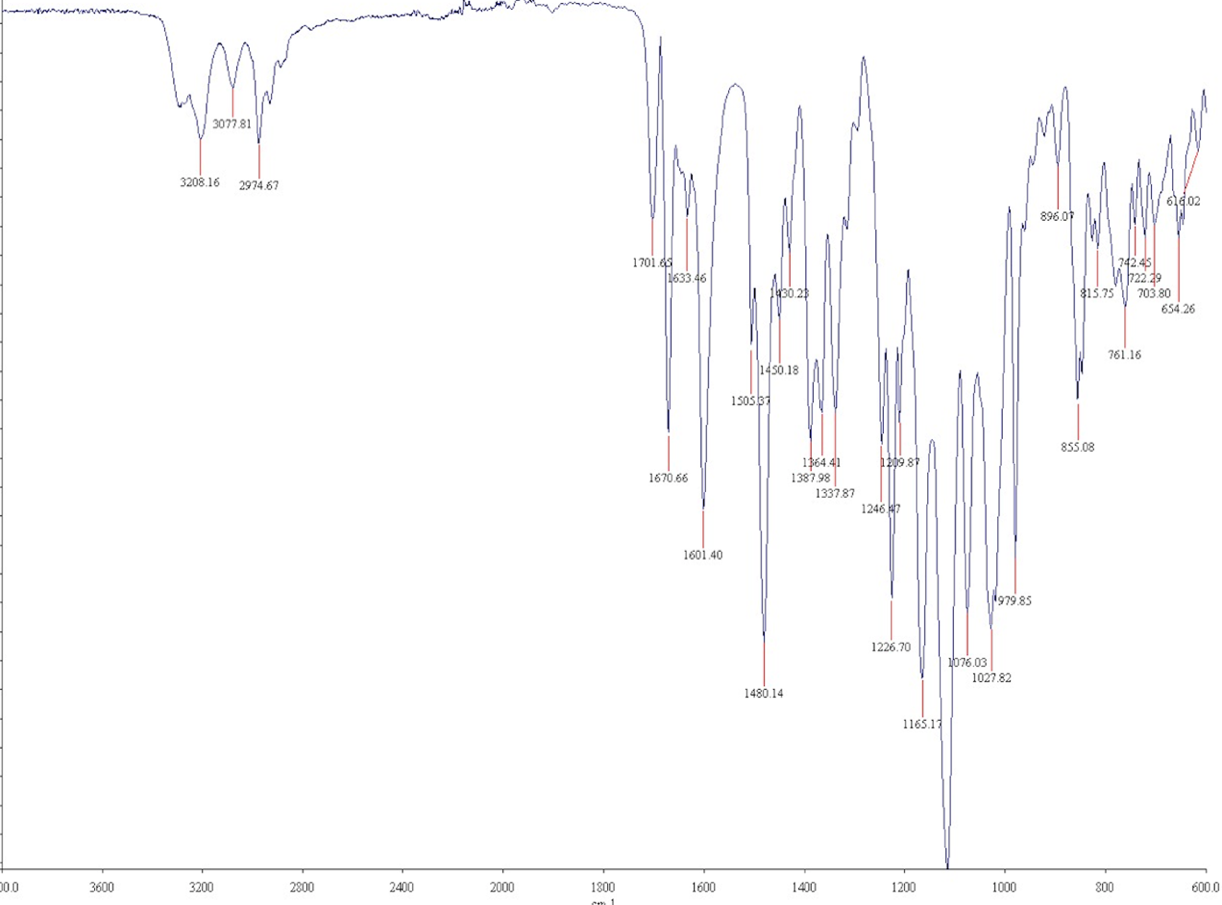


^1^H NMR spectrum of compound **1e**


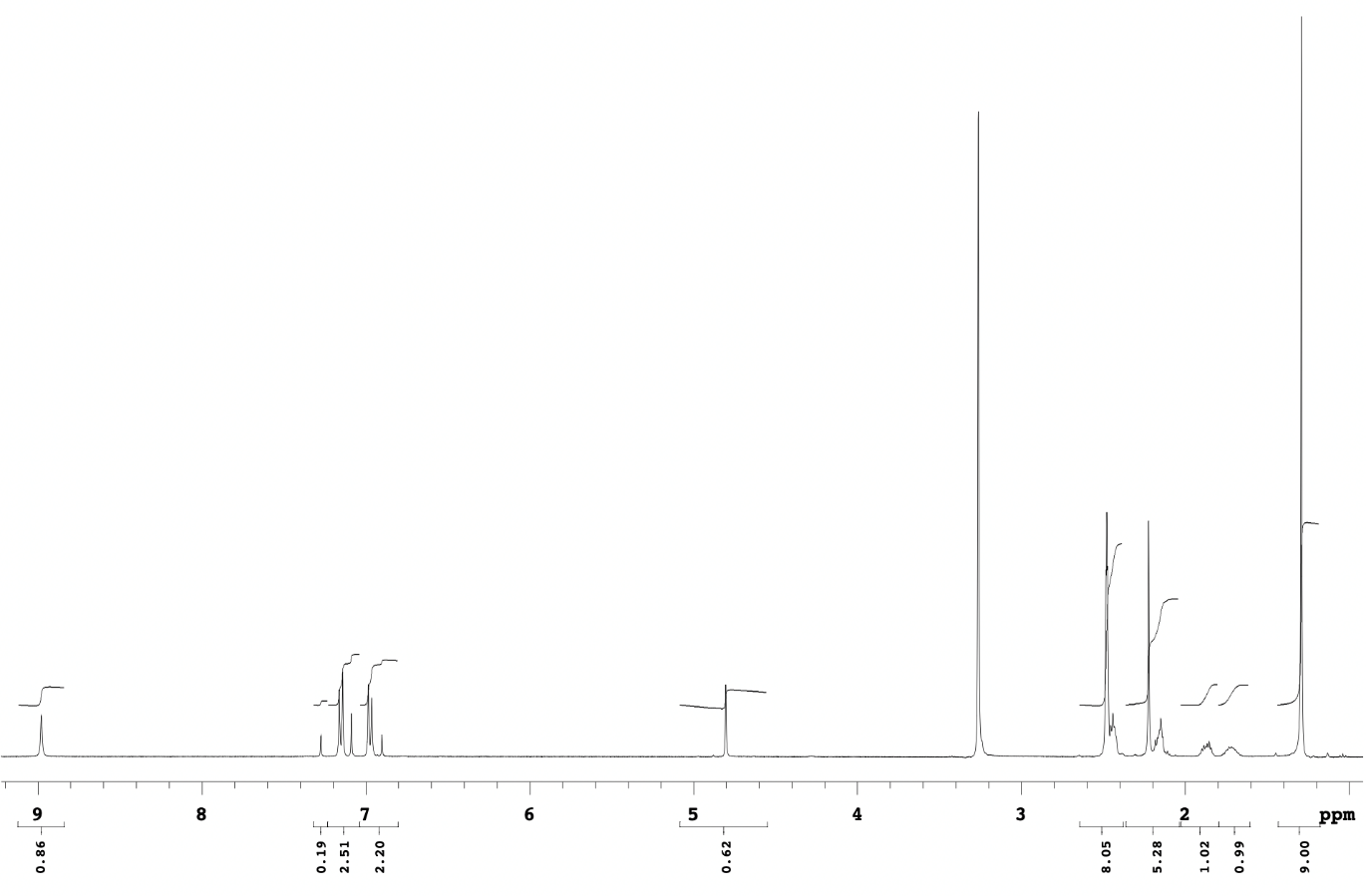

^13^C NMR spectrum of compound **1e**


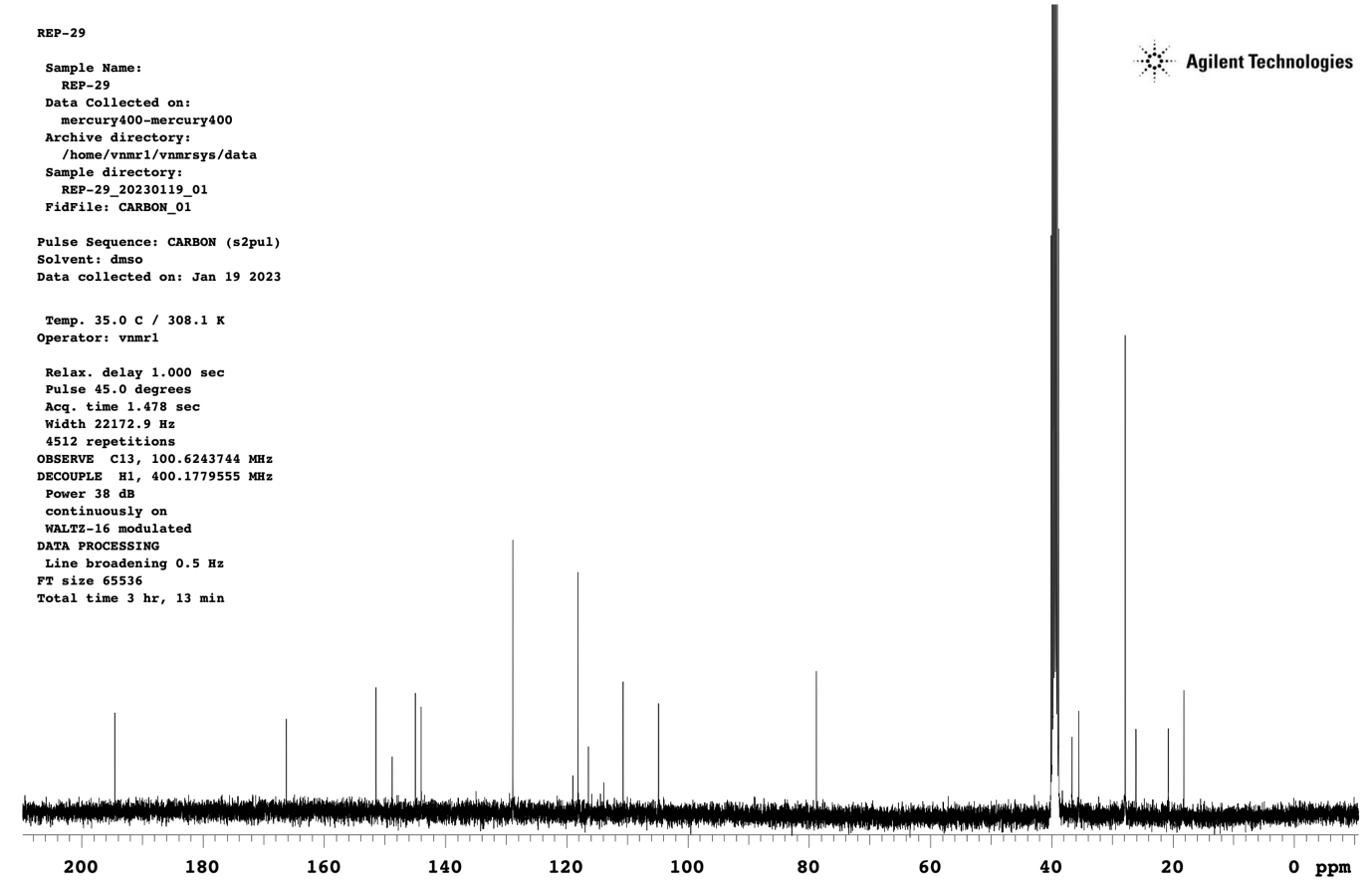

Mass-spectrum of compound **1e**


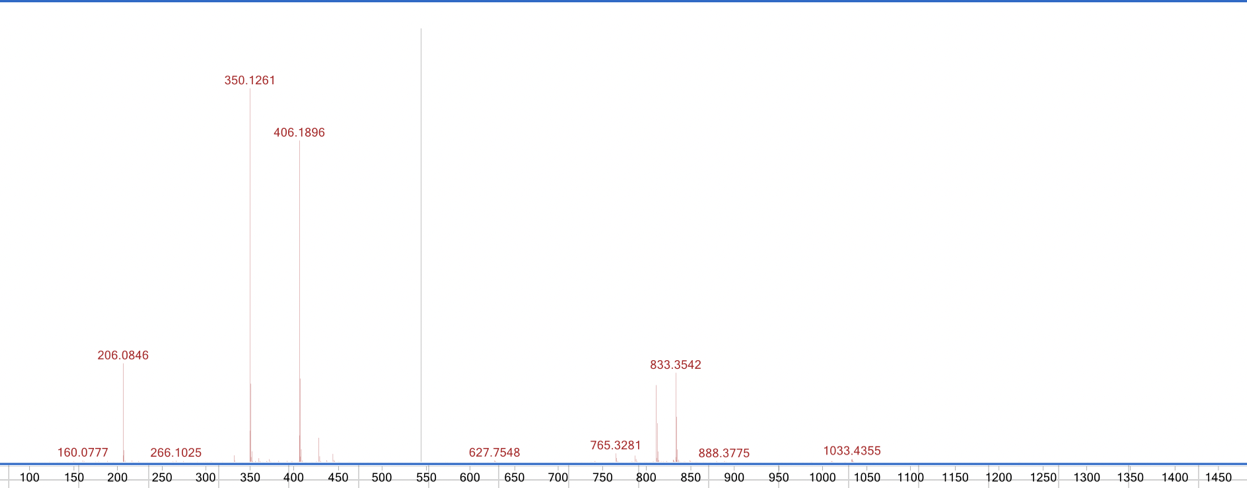


IR spectrum of compound **2a**


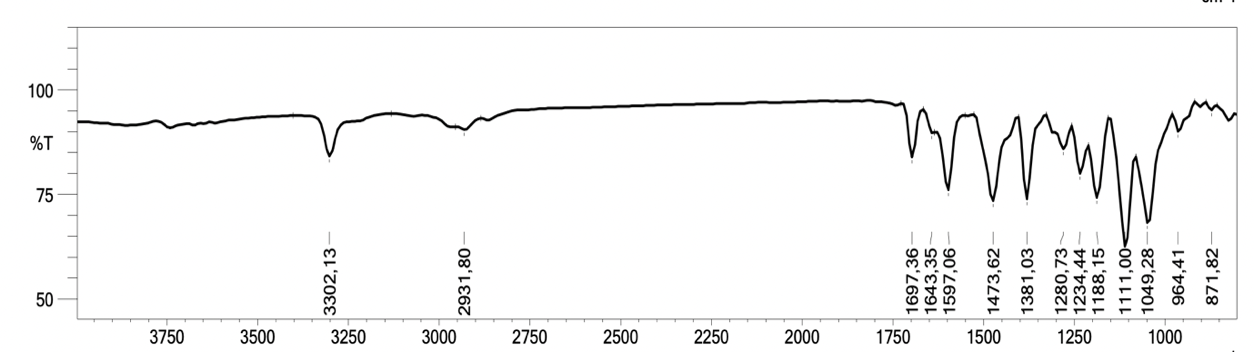


^1^H NMR spectrum of compound **2a**


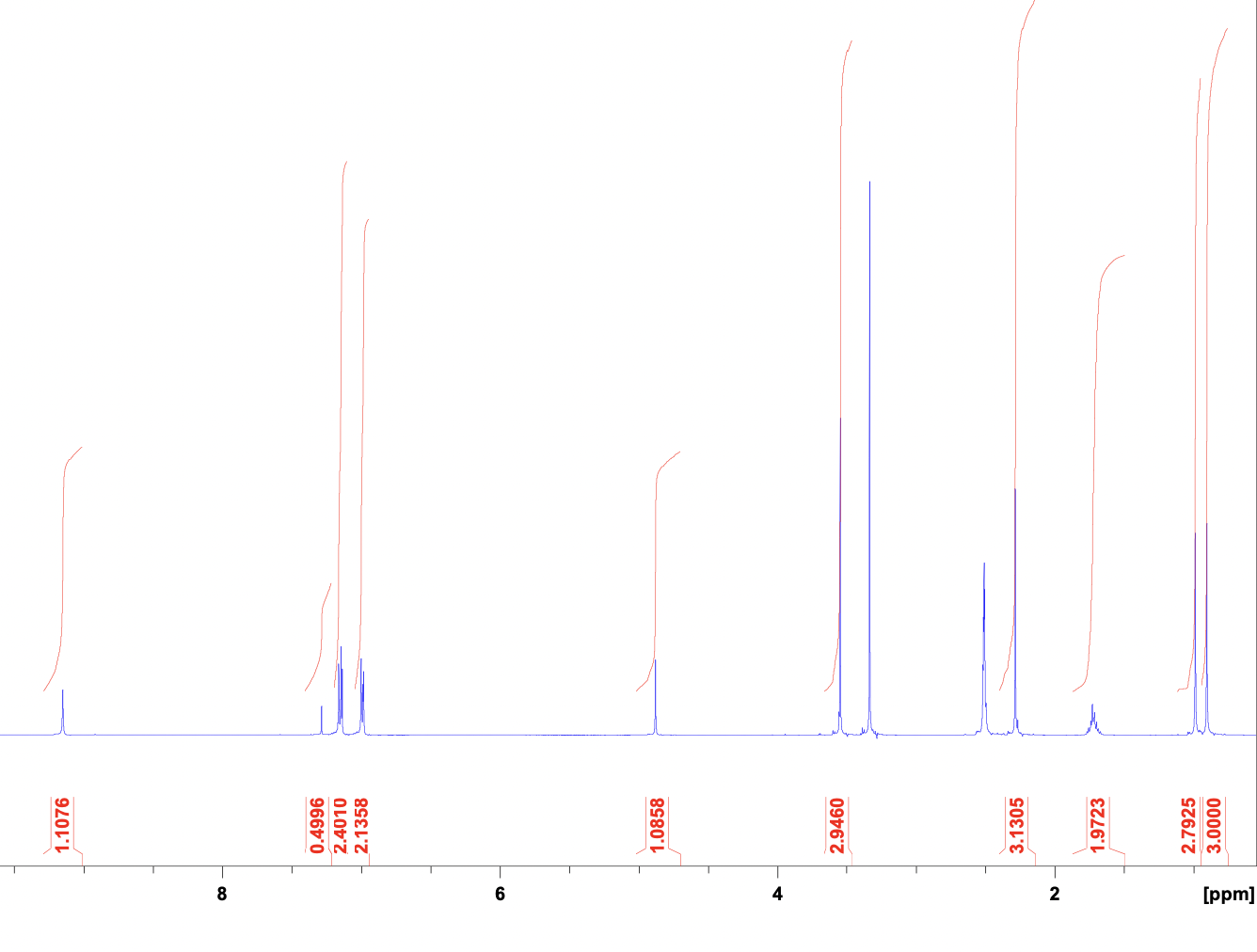

^13^C NMR spectrum of compound **2a**


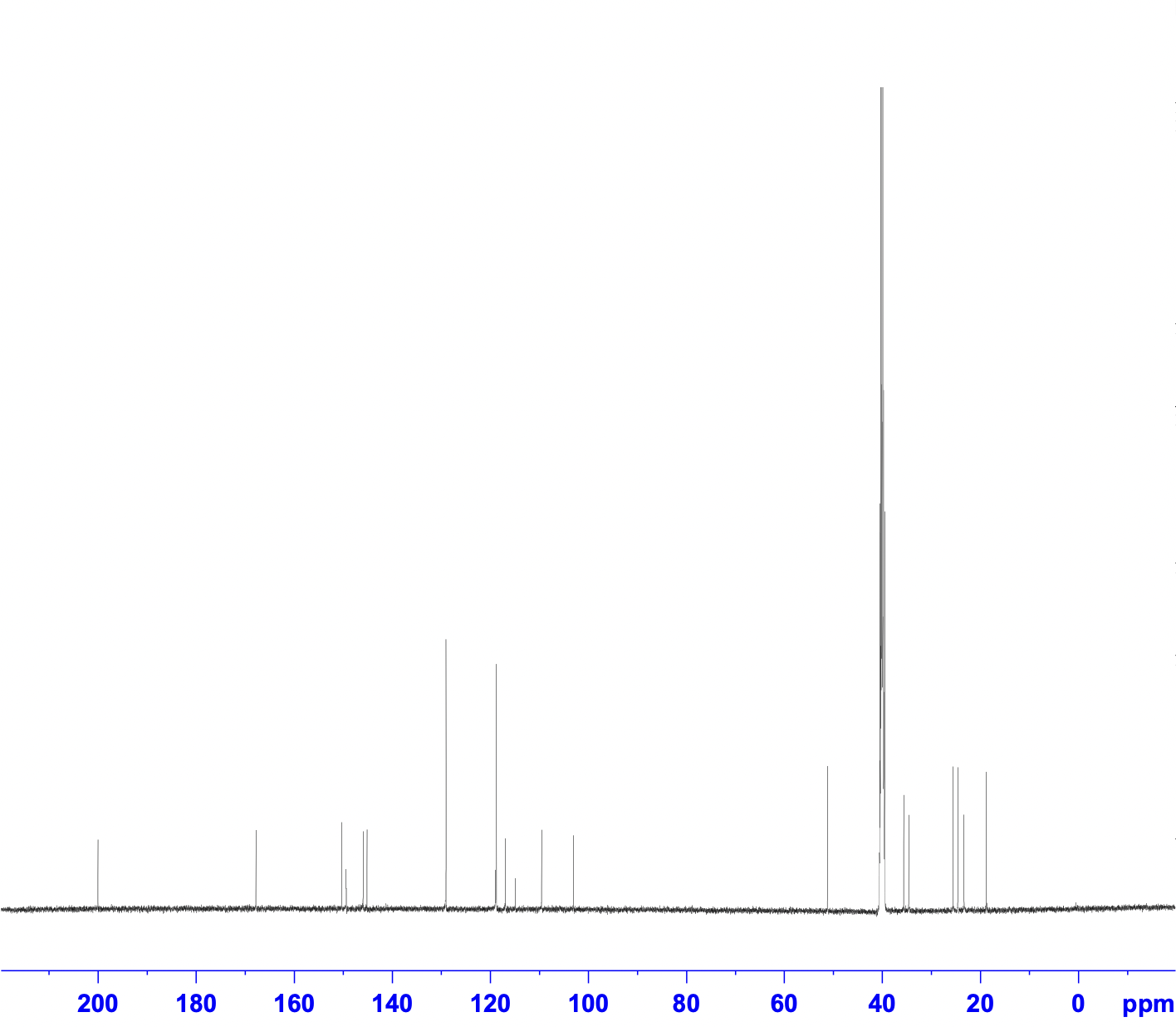

Mass-spectrum of compound **2a**


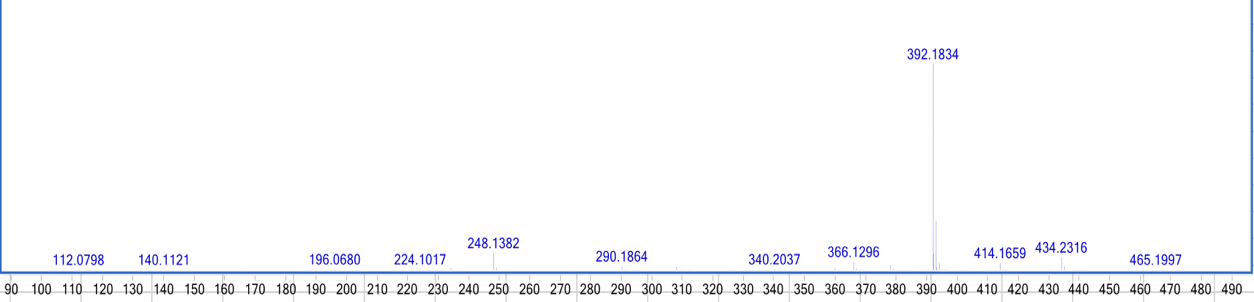


IR spectrum of compound **2b**


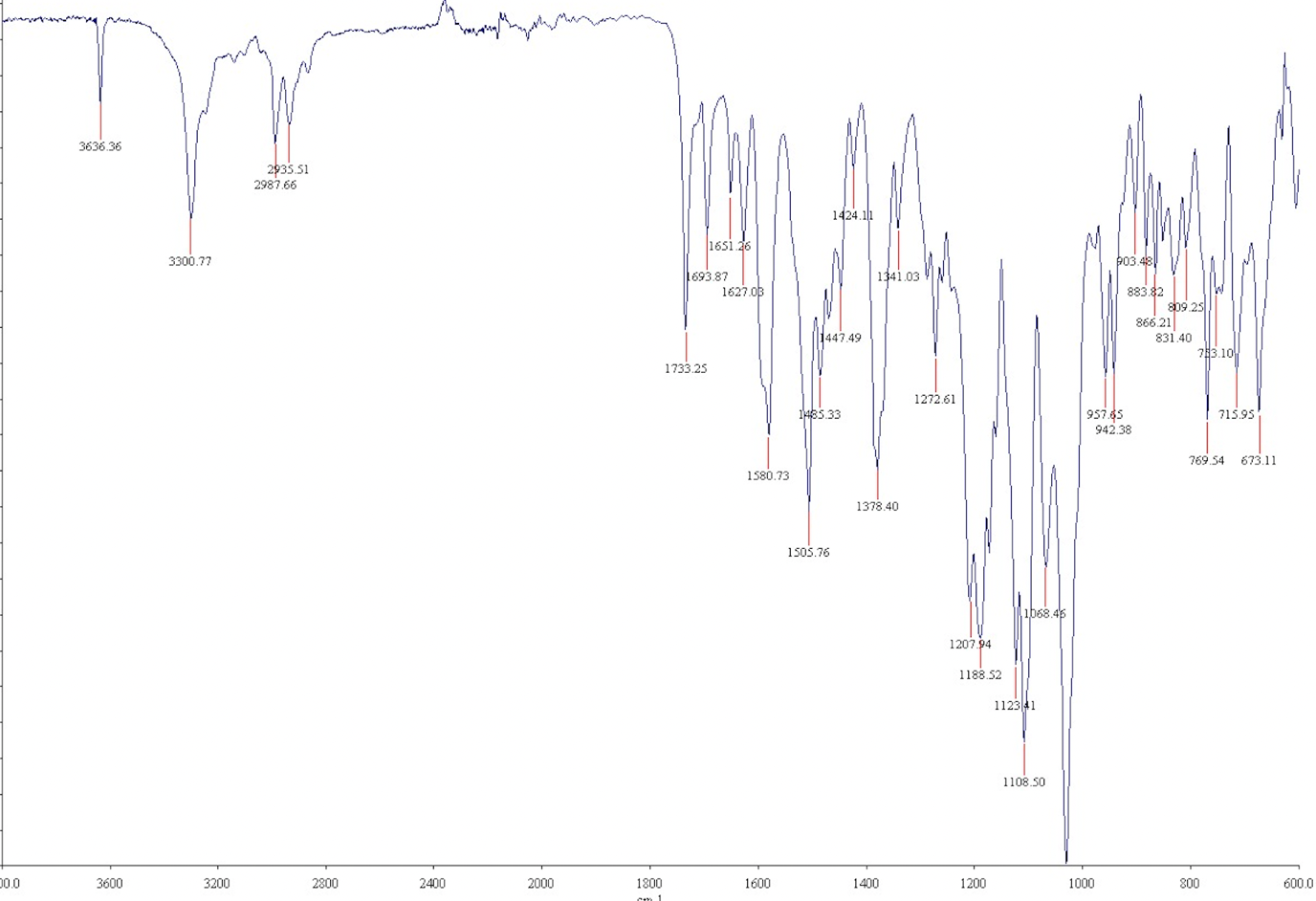


^1^H NMR spectrum of compound **2b**


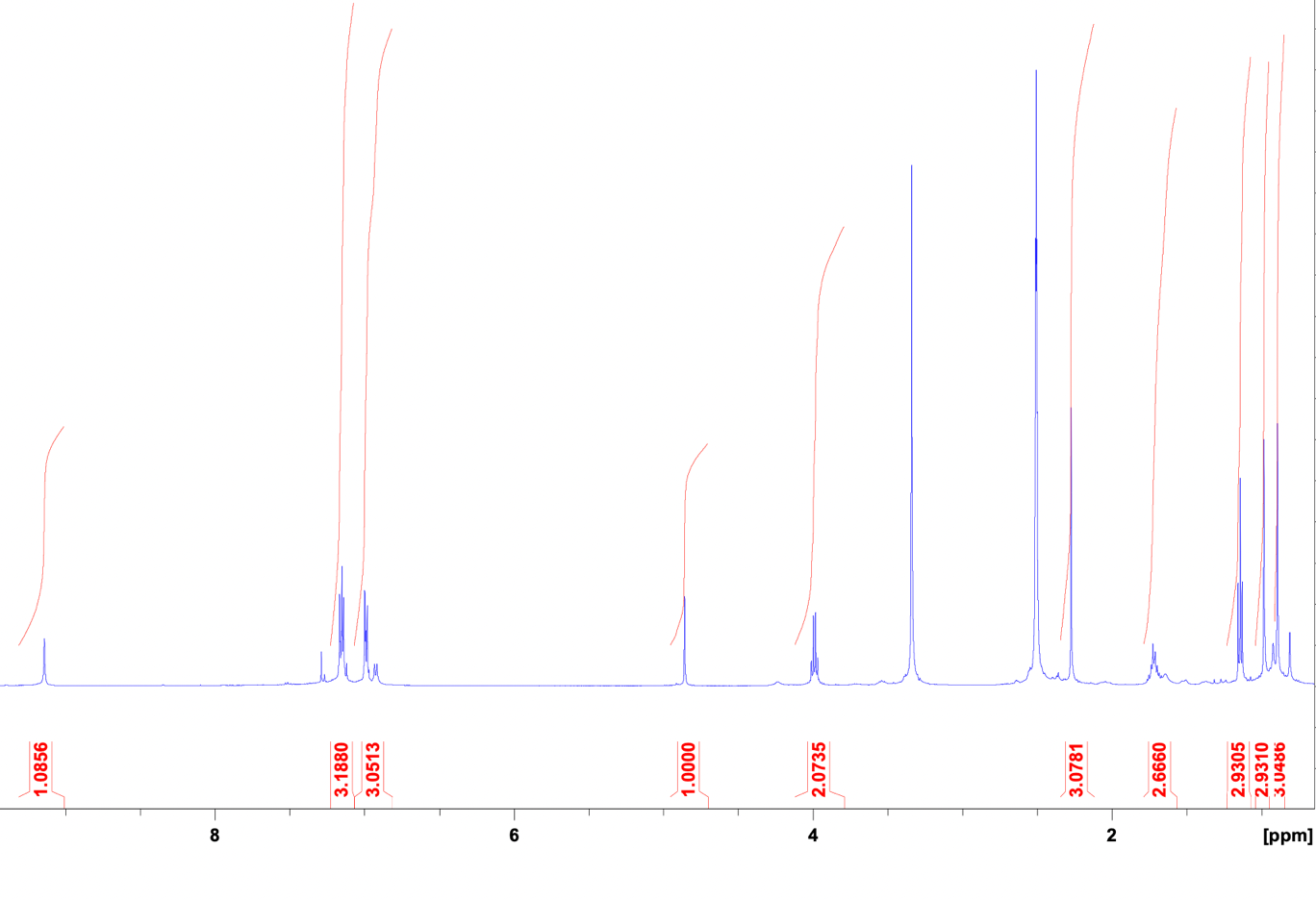

Mass-spectrum of compound **2b**


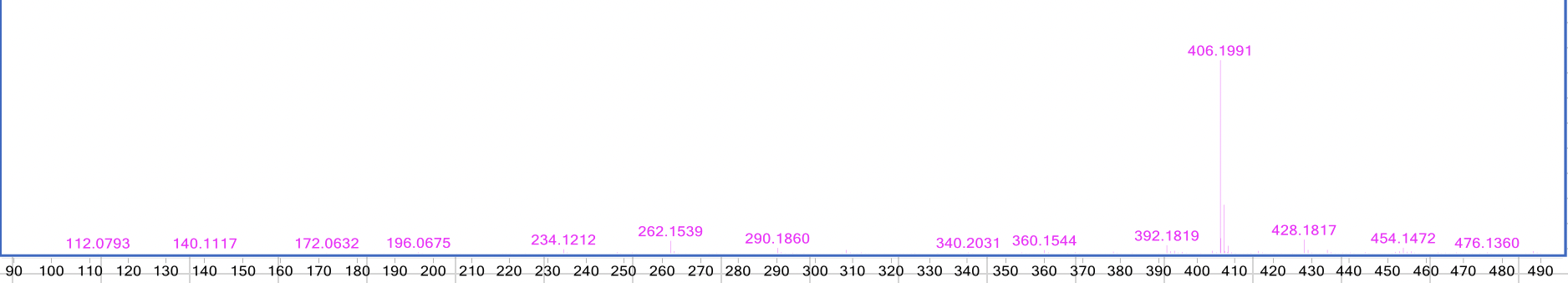


IR spectrum of compound **2c**


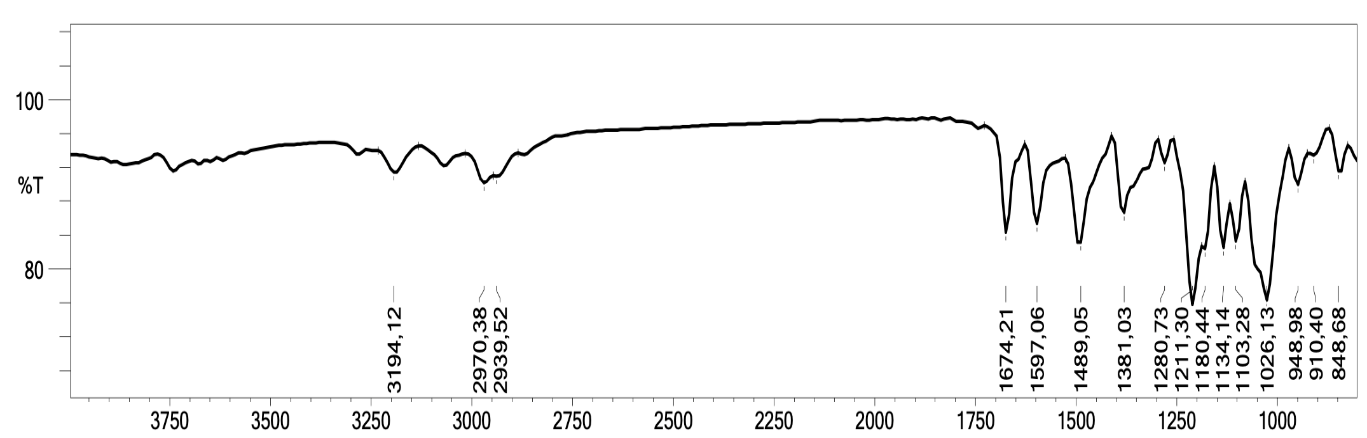


^1^H NMR spectrum of compound **2c**


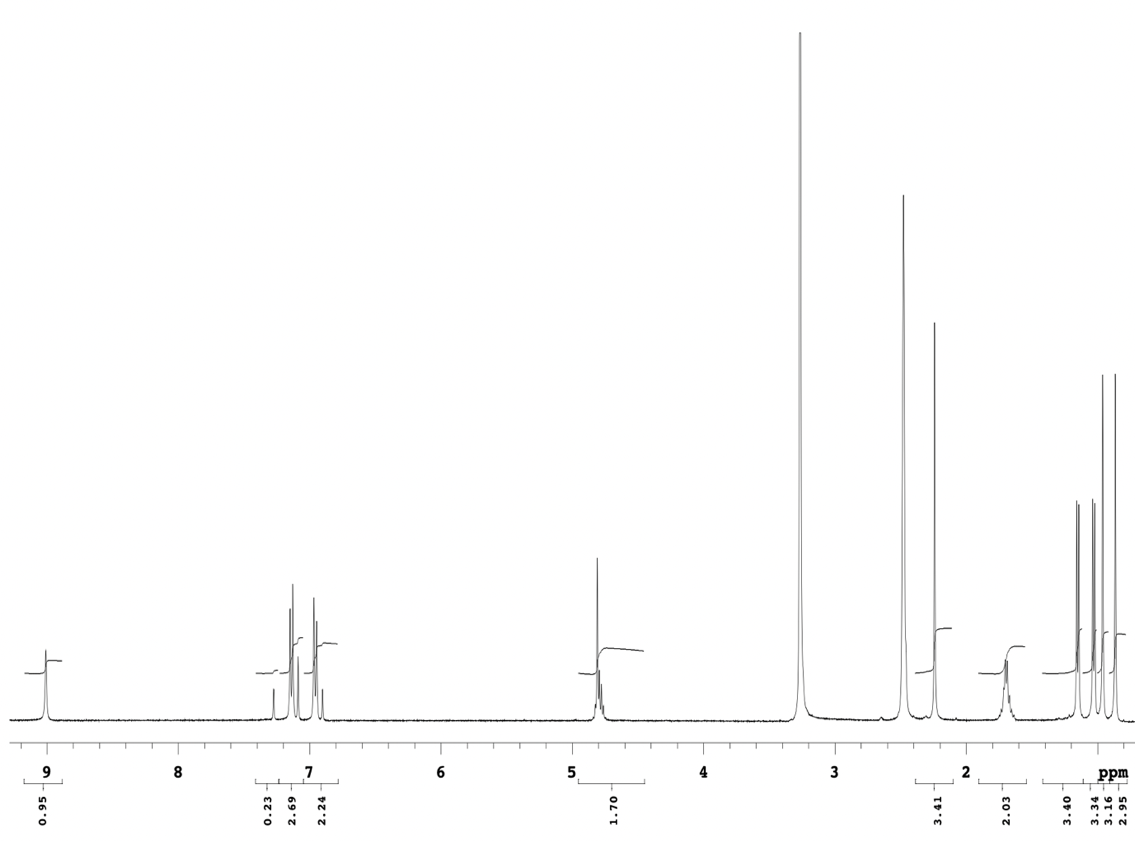

^13^C NMR spectrum of compound **2c**


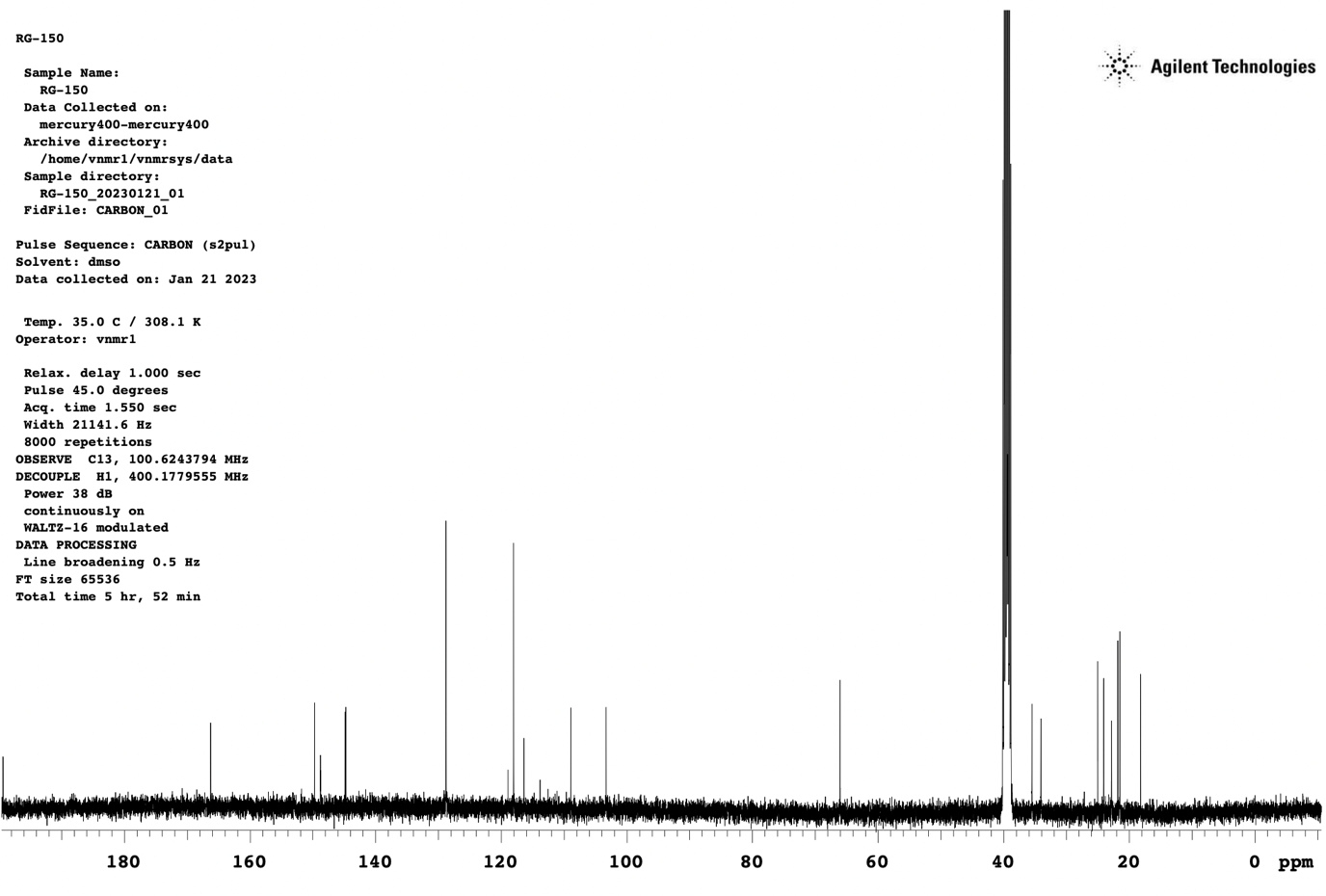

Mass-spectrum of compound **2c**


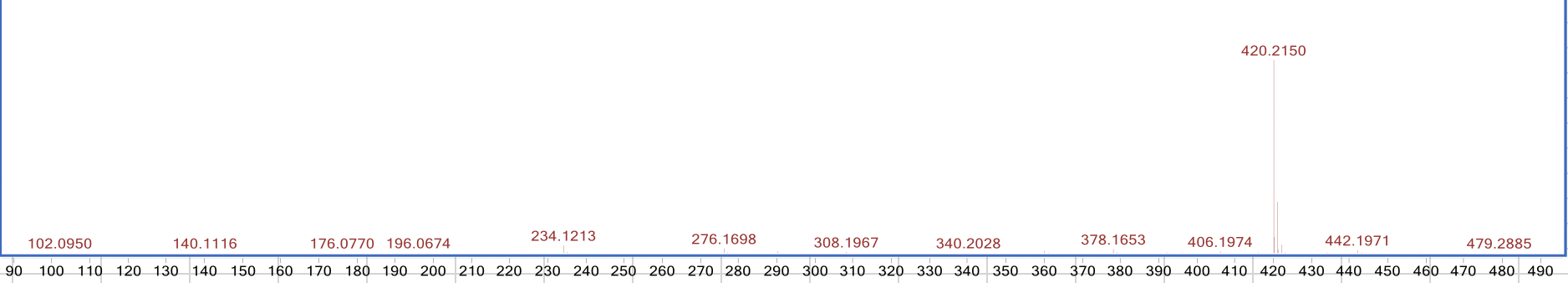


IR spectrum of compound **2d**


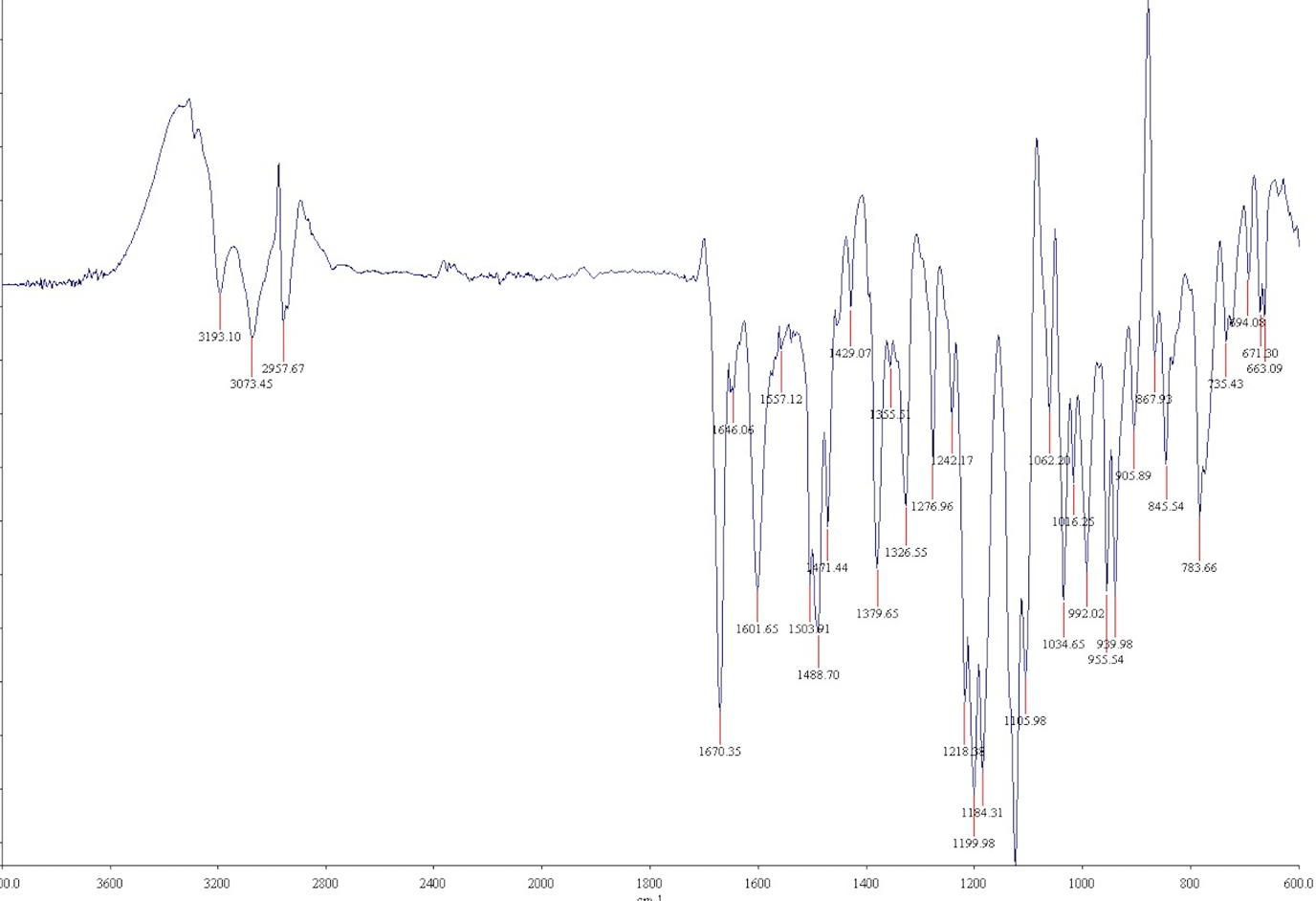


^1^H NMR spectrum of compound **2d**


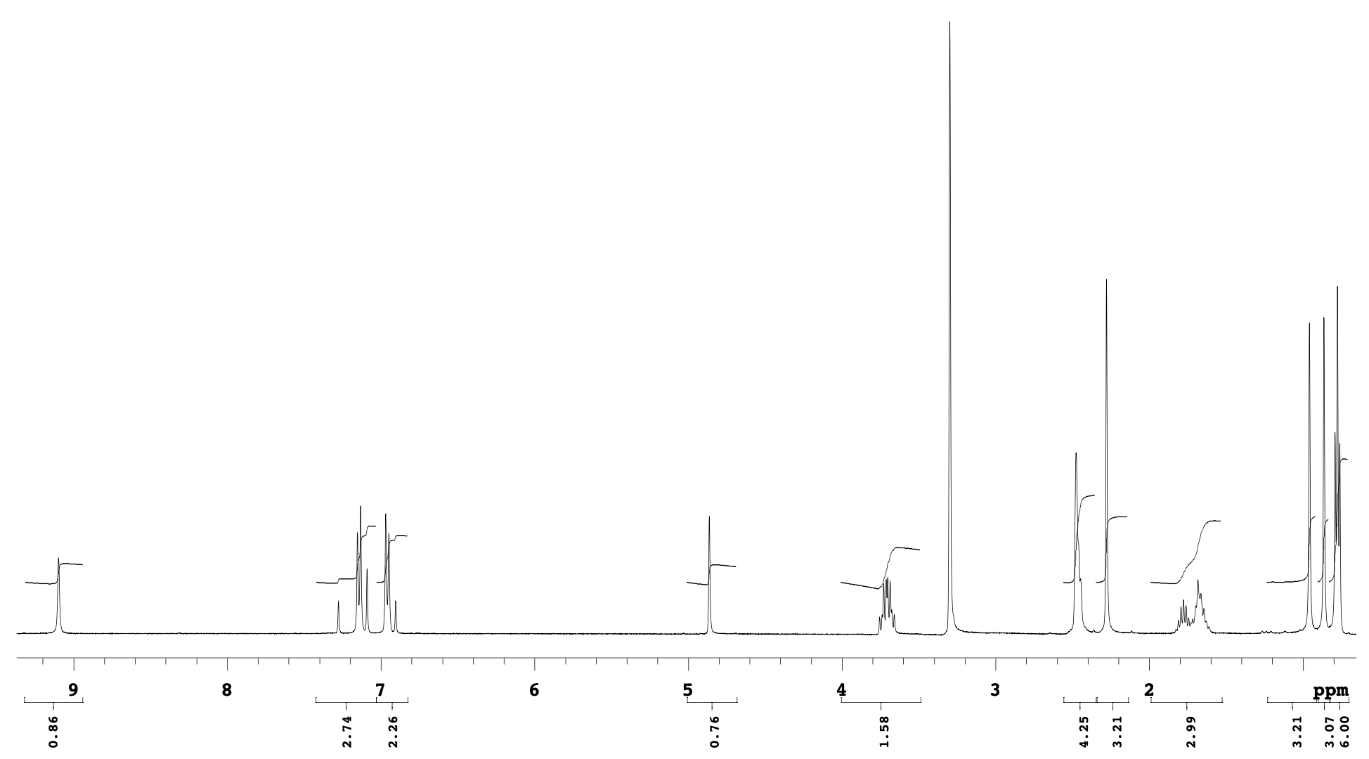

^13^C NMR spectrum of compound **2d**


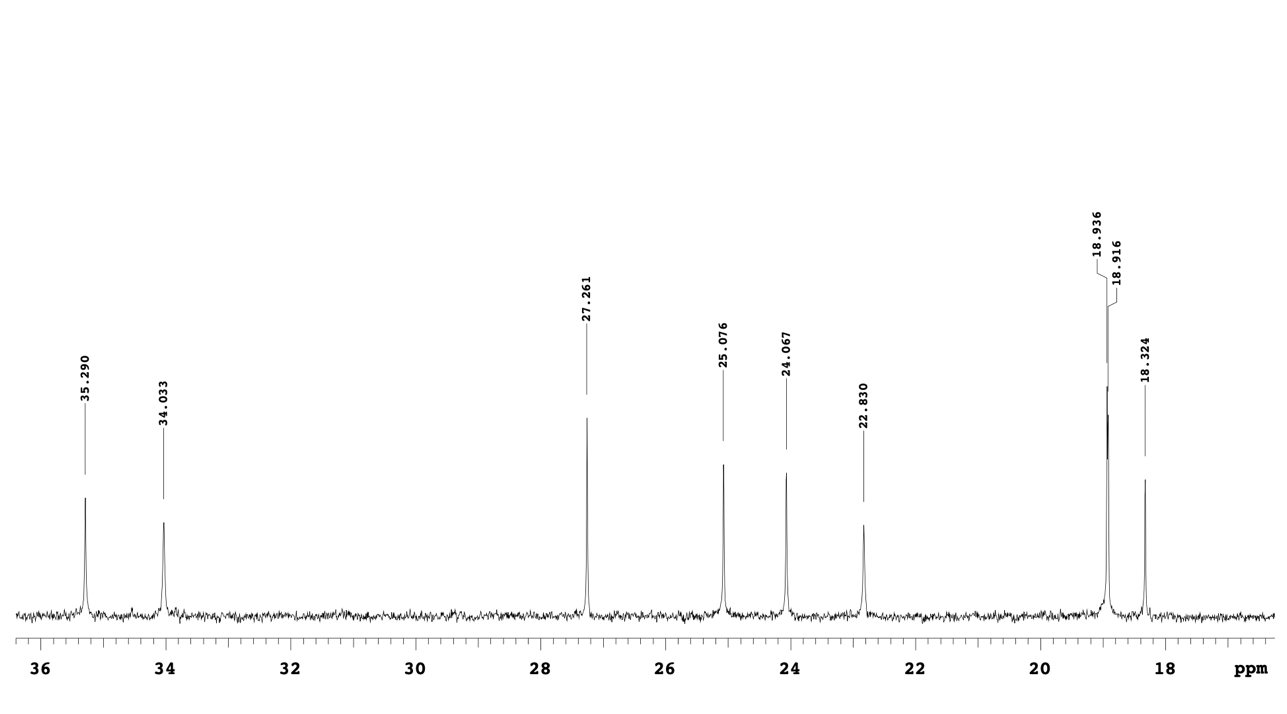


^13^C NMR spectrum of compound **2d**


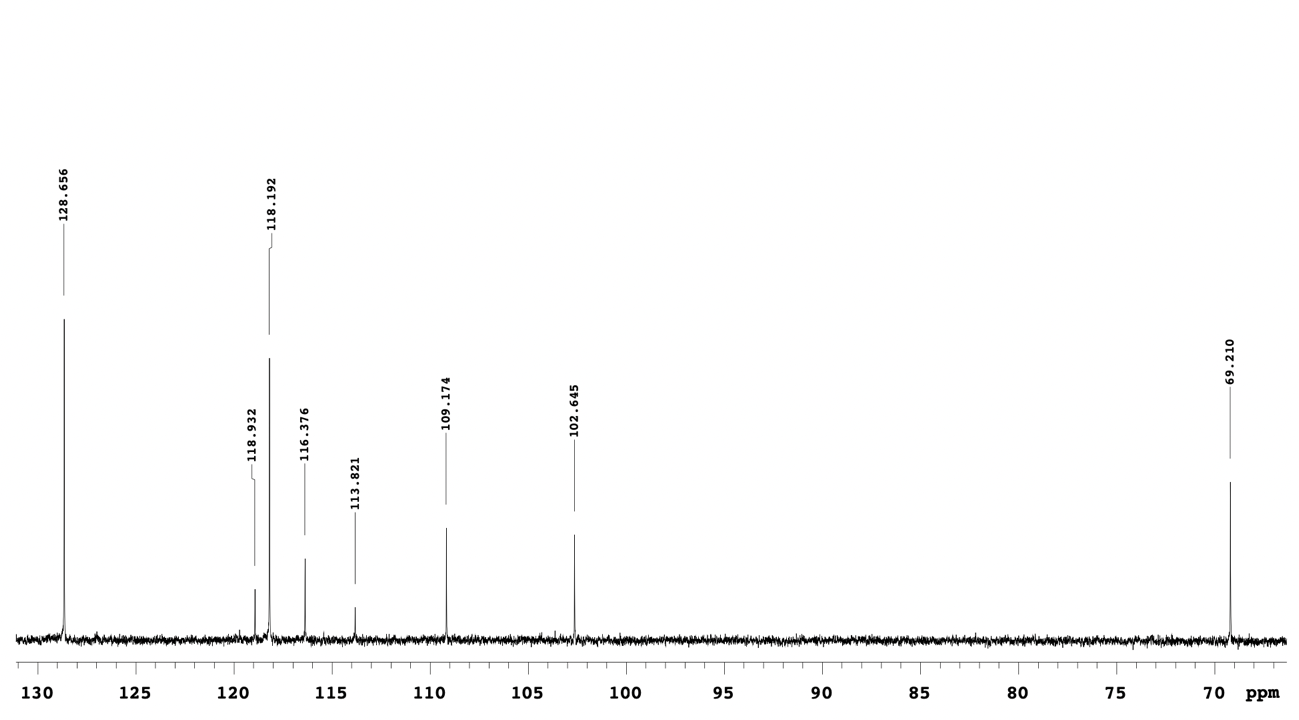

^13^C NMR spectrum of compound **2d**


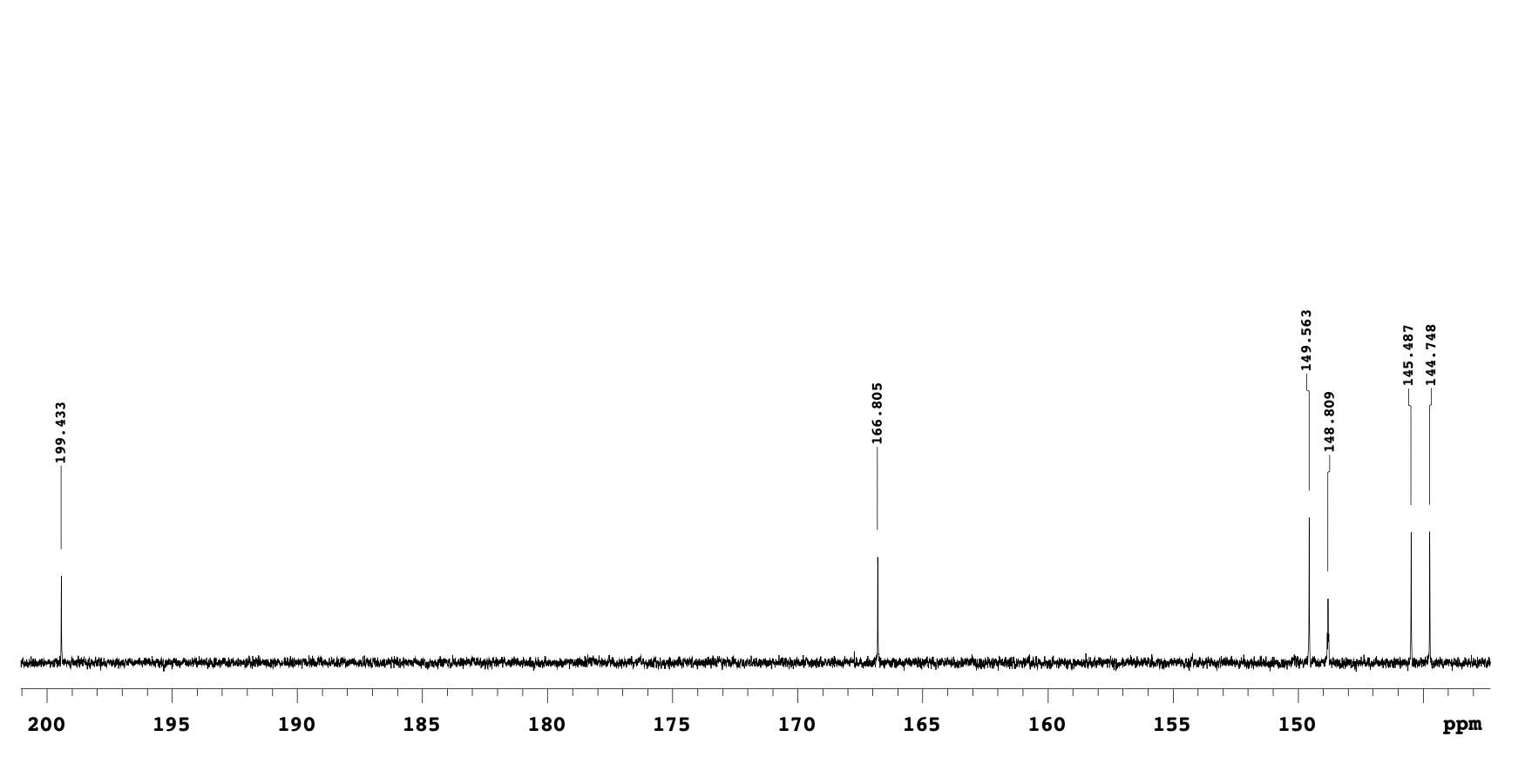


Mass-spectrum of compound **2d**


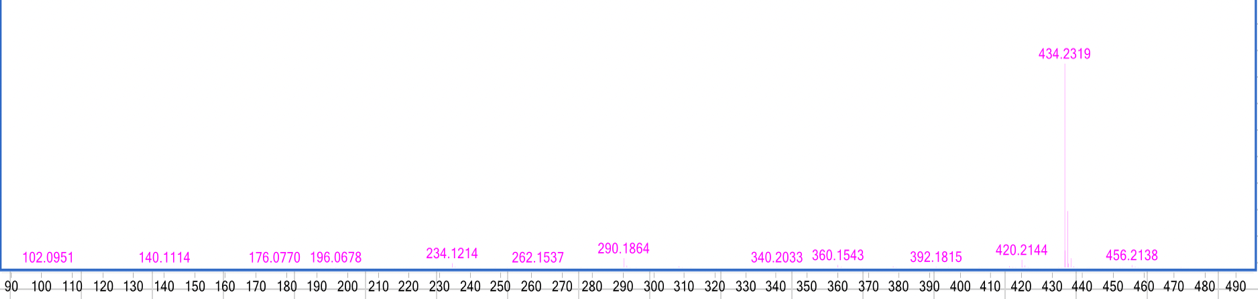


IR spectrum of compound **2e**


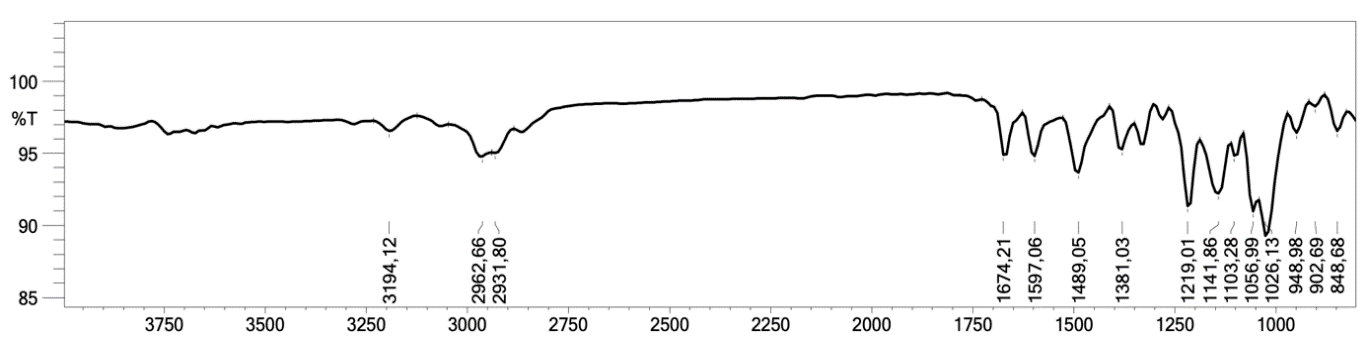


^1^H NMR spectrum of compound **2e**


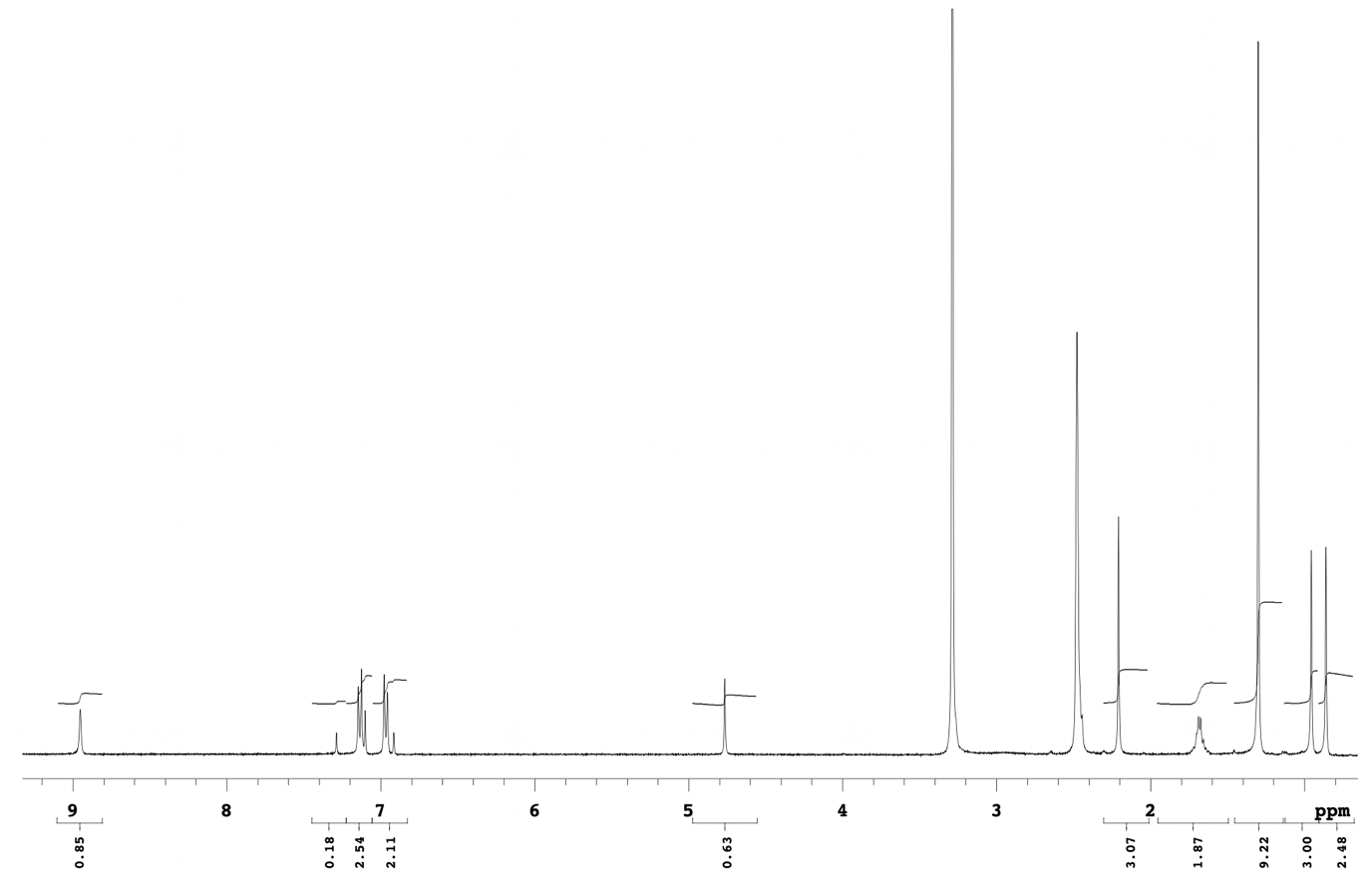

^13^C NMR spectrum of compound **2e**


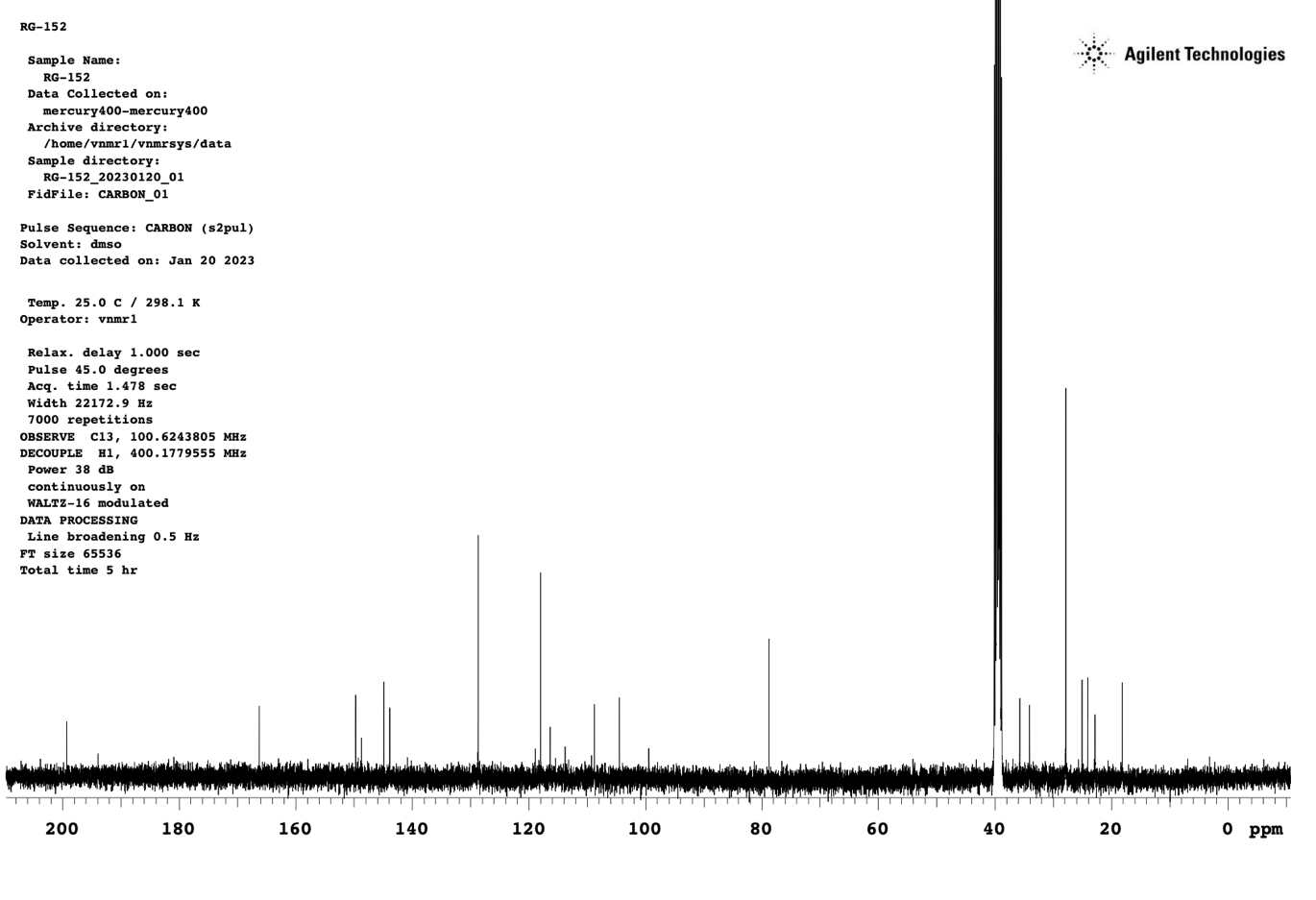

Mass-spectrum of compound **2e**


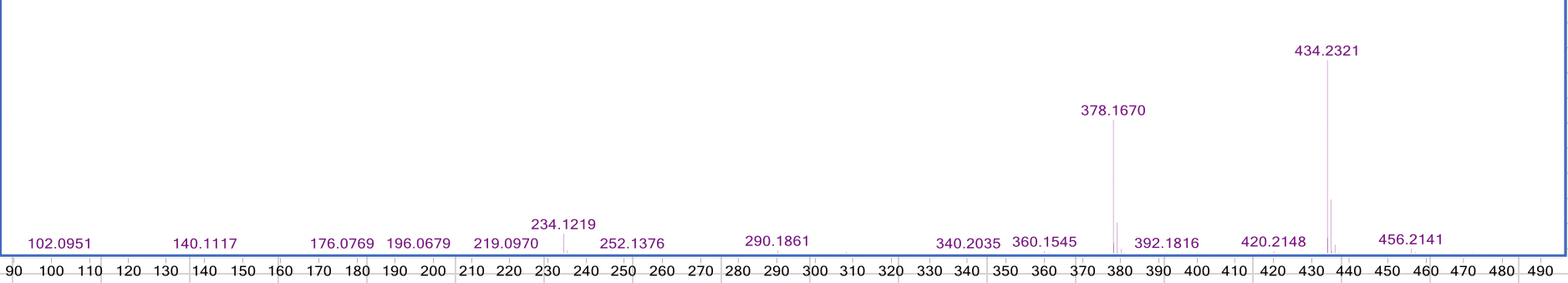


IR spectrum of compound **3a**


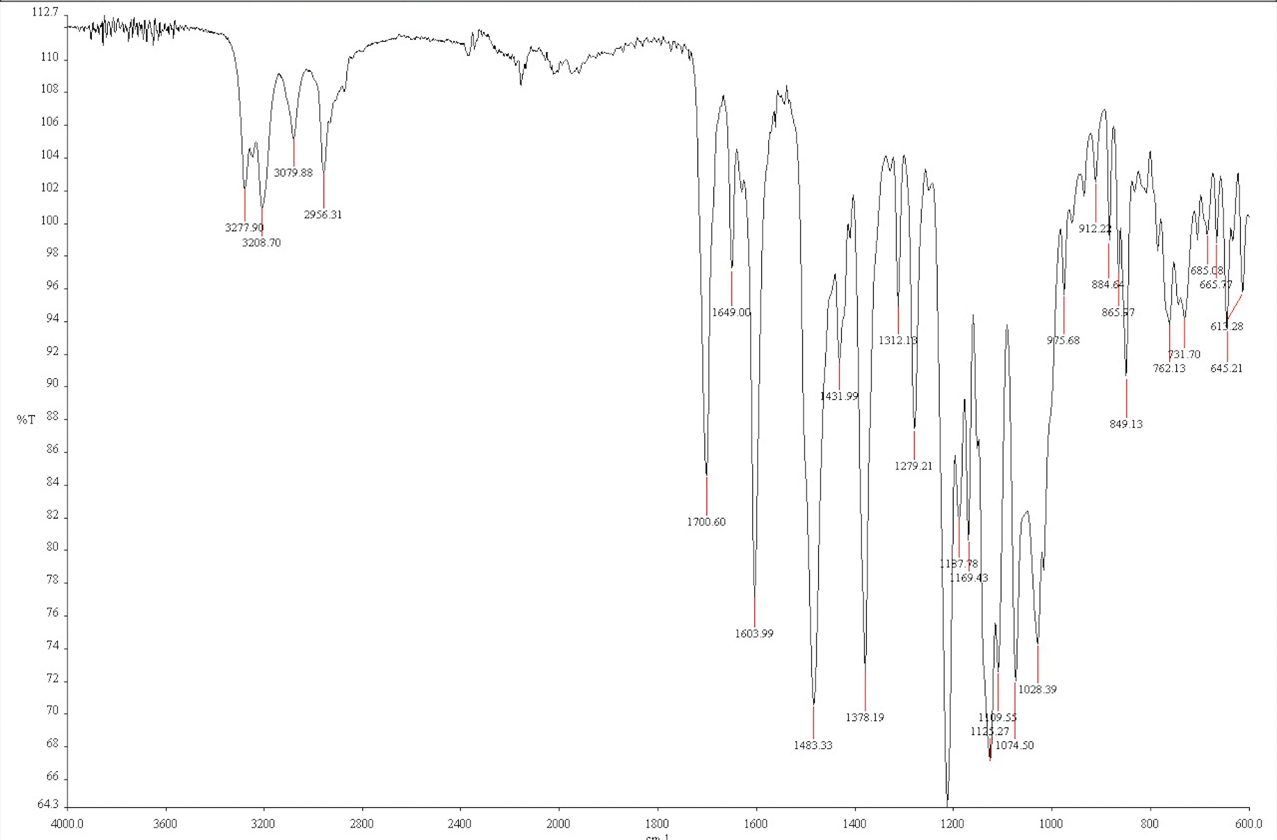


^1^H NMR spectrum of compound **3a**


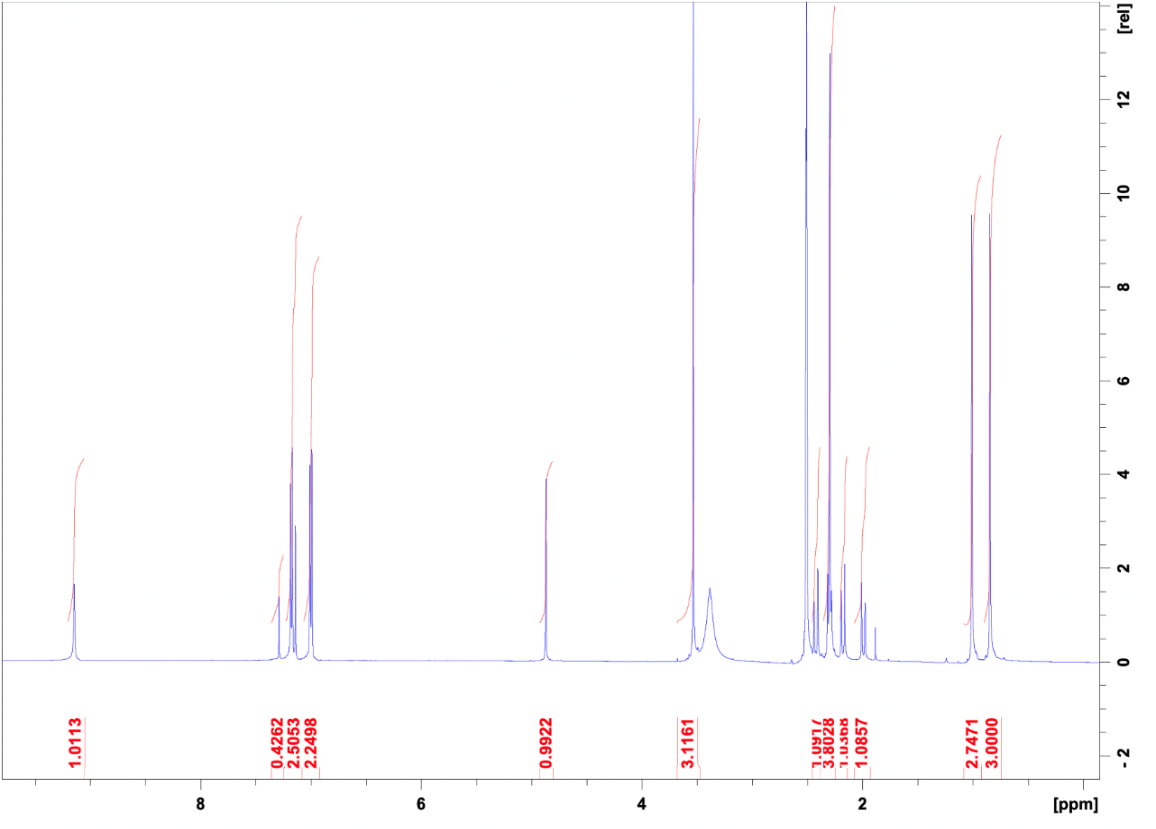

^13^C NMR spectrum of compound **3a**


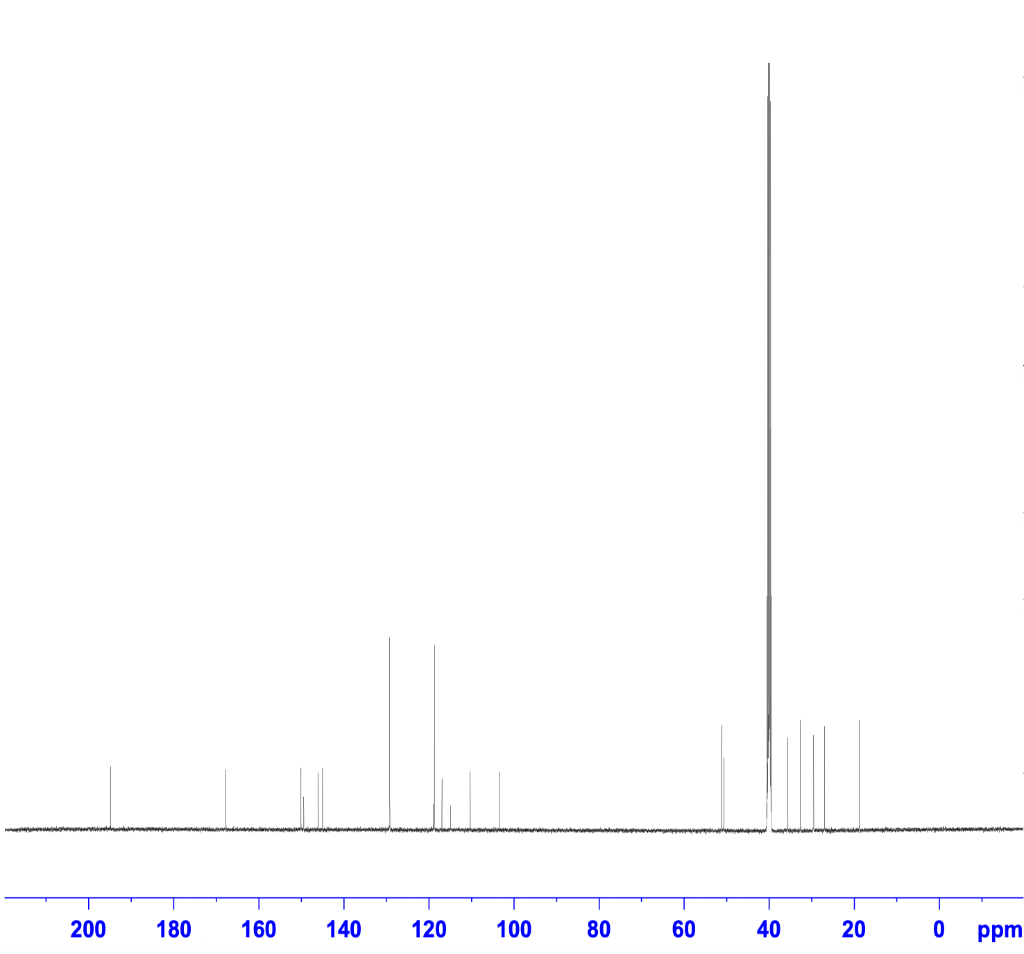

Mass-spectrum of compound **3a**


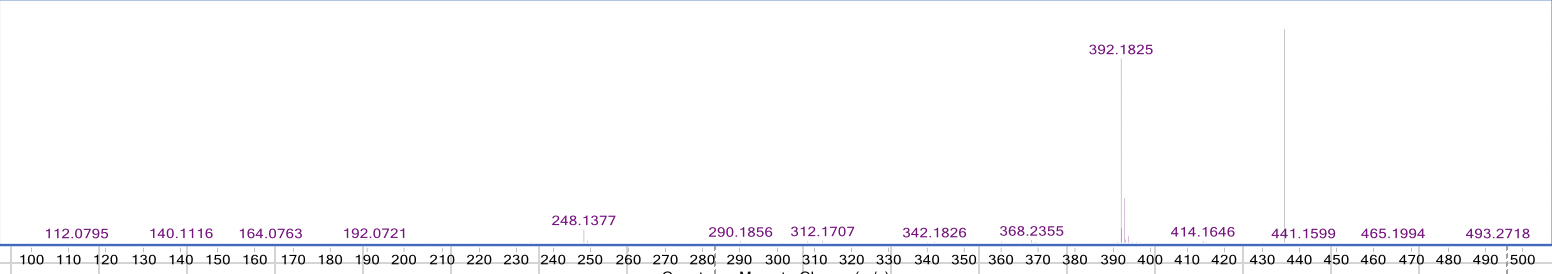


IR spectrum of compound **3b**


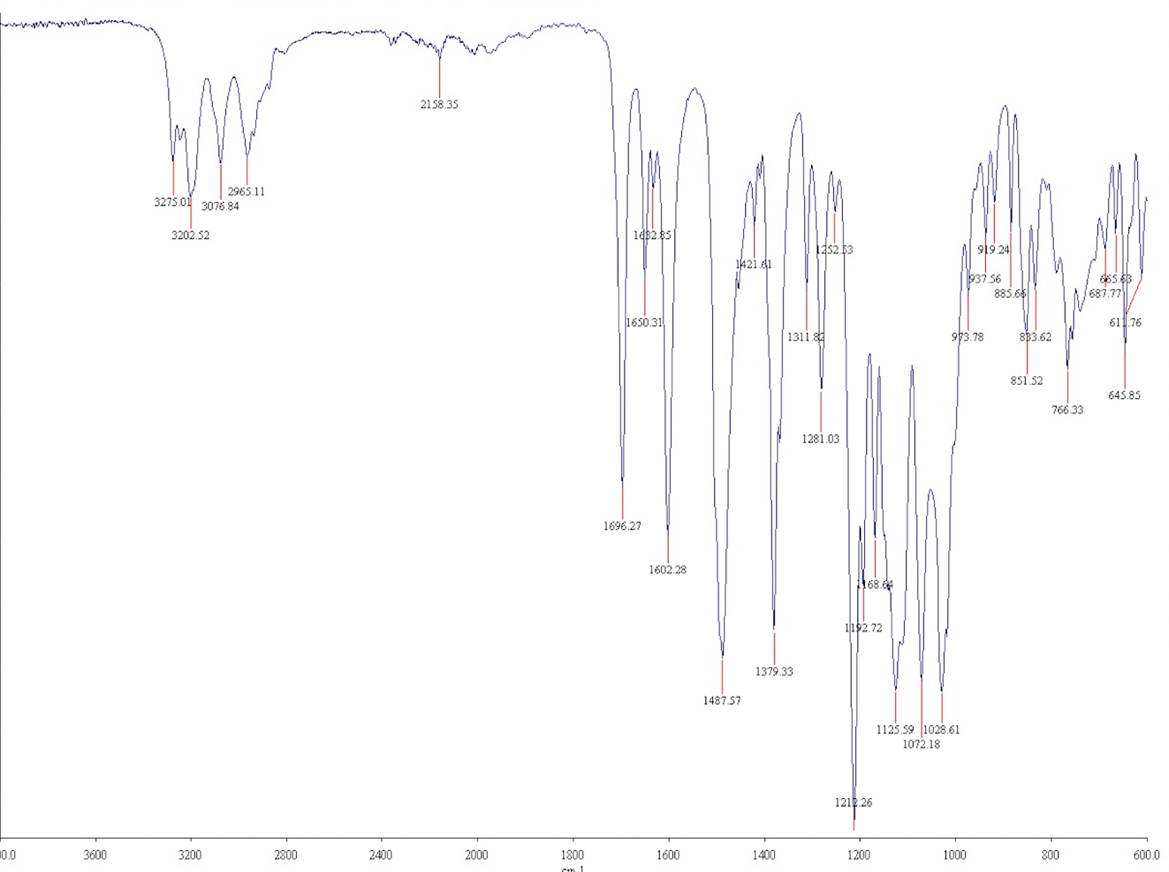


^1^H NMR spectrum of compound **3b**


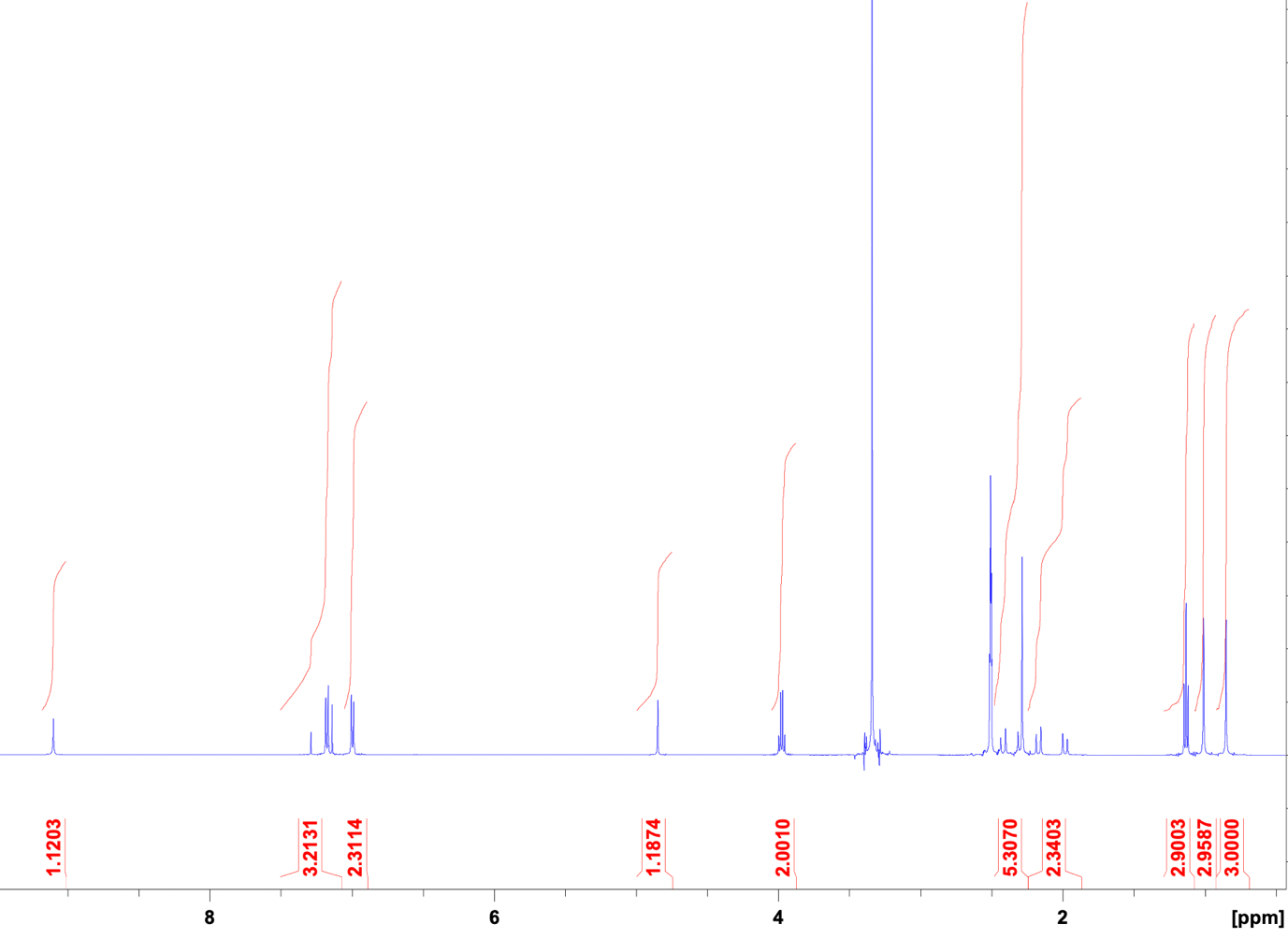

^13^C NMR spectrum of compound **3b**


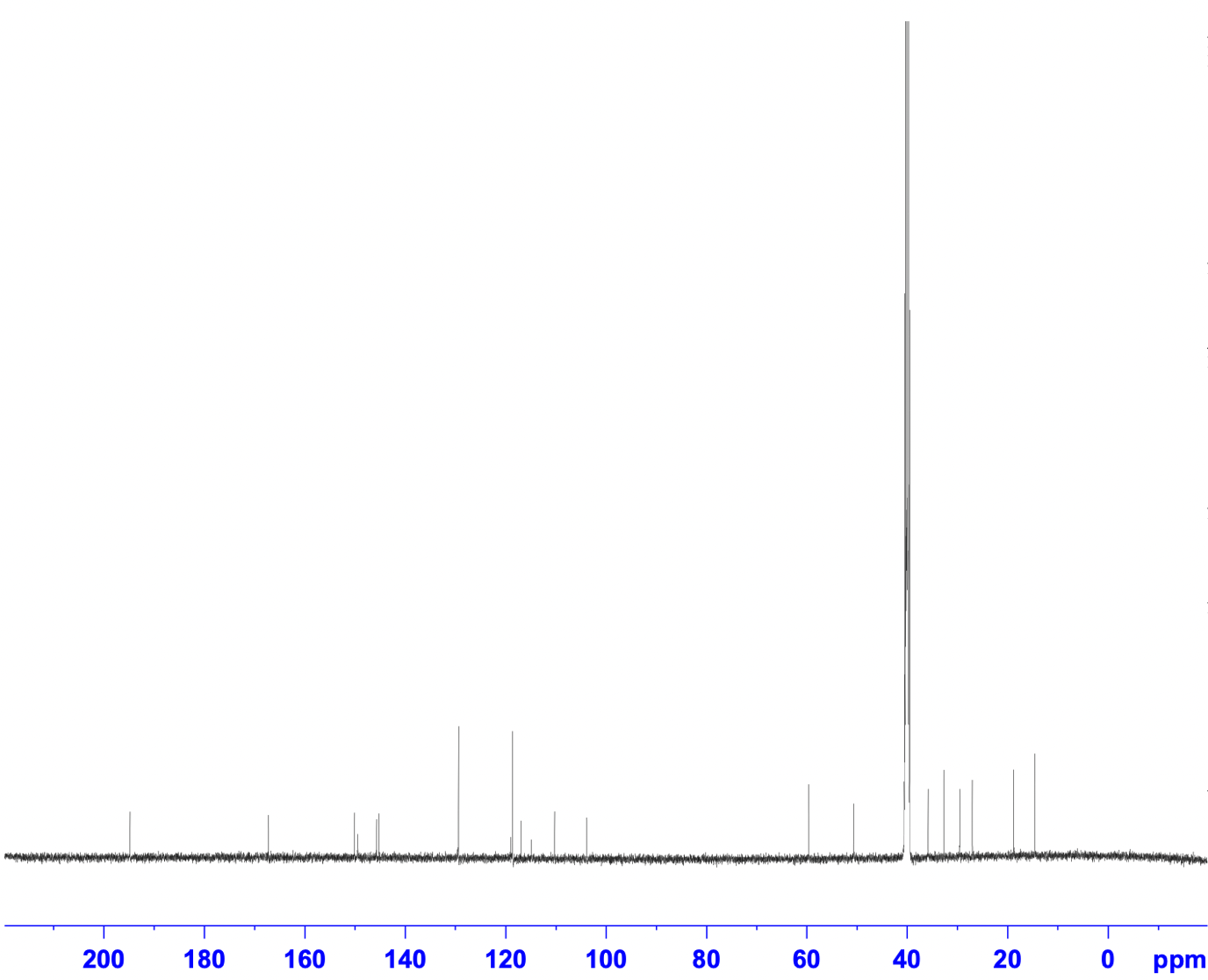

Mass-spectrum of compound **3b**


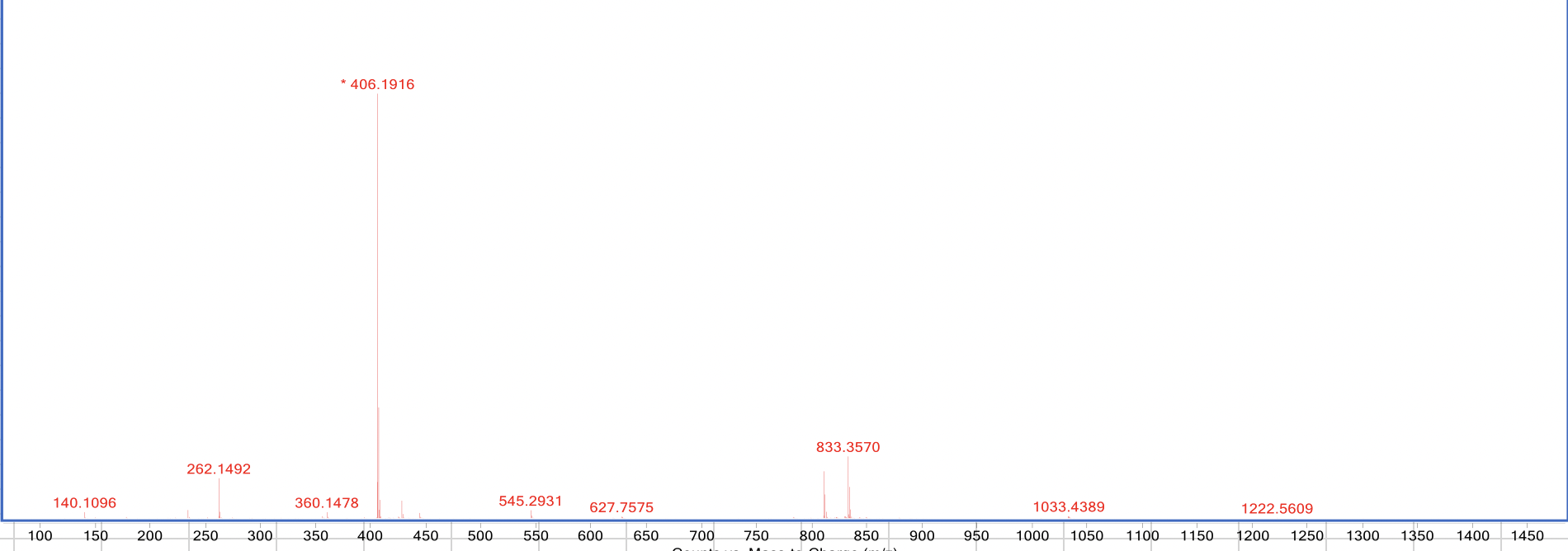


IR spectrum of compound **3c**


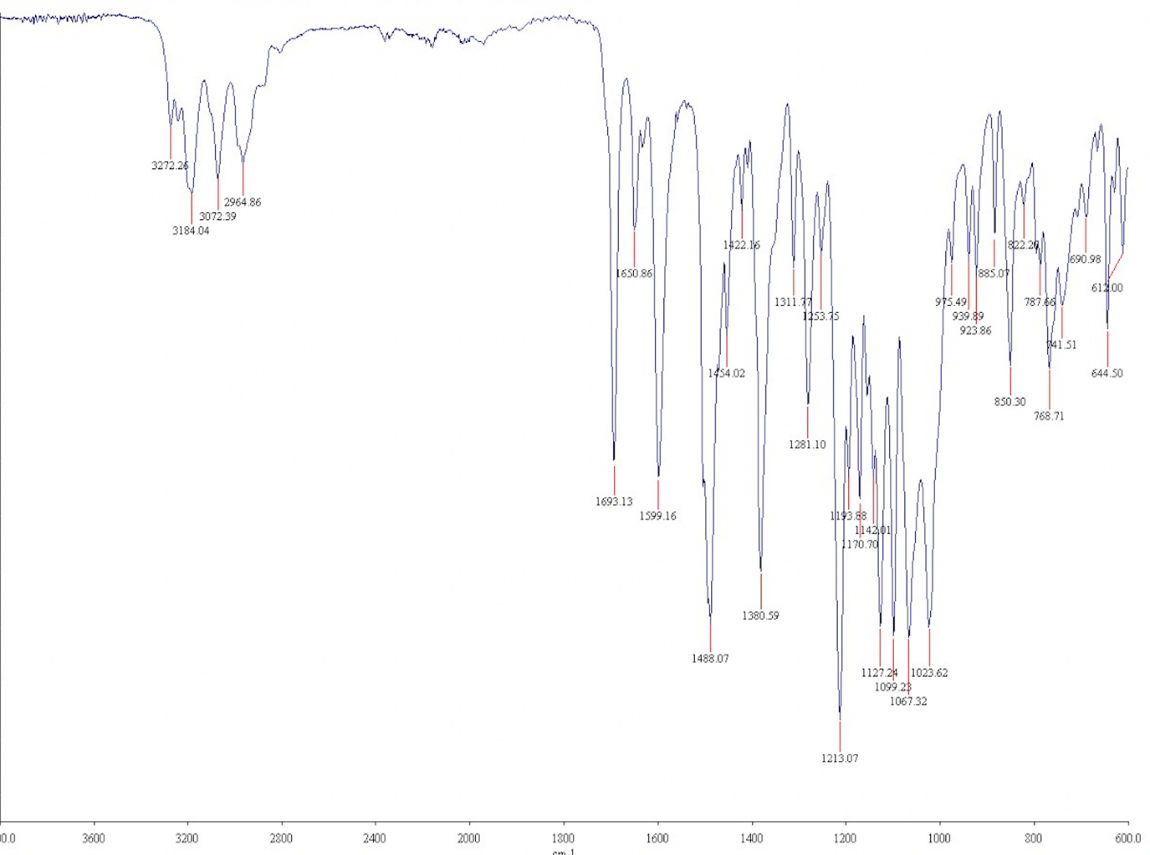


^1^H NMR spectrum of compound **3c**


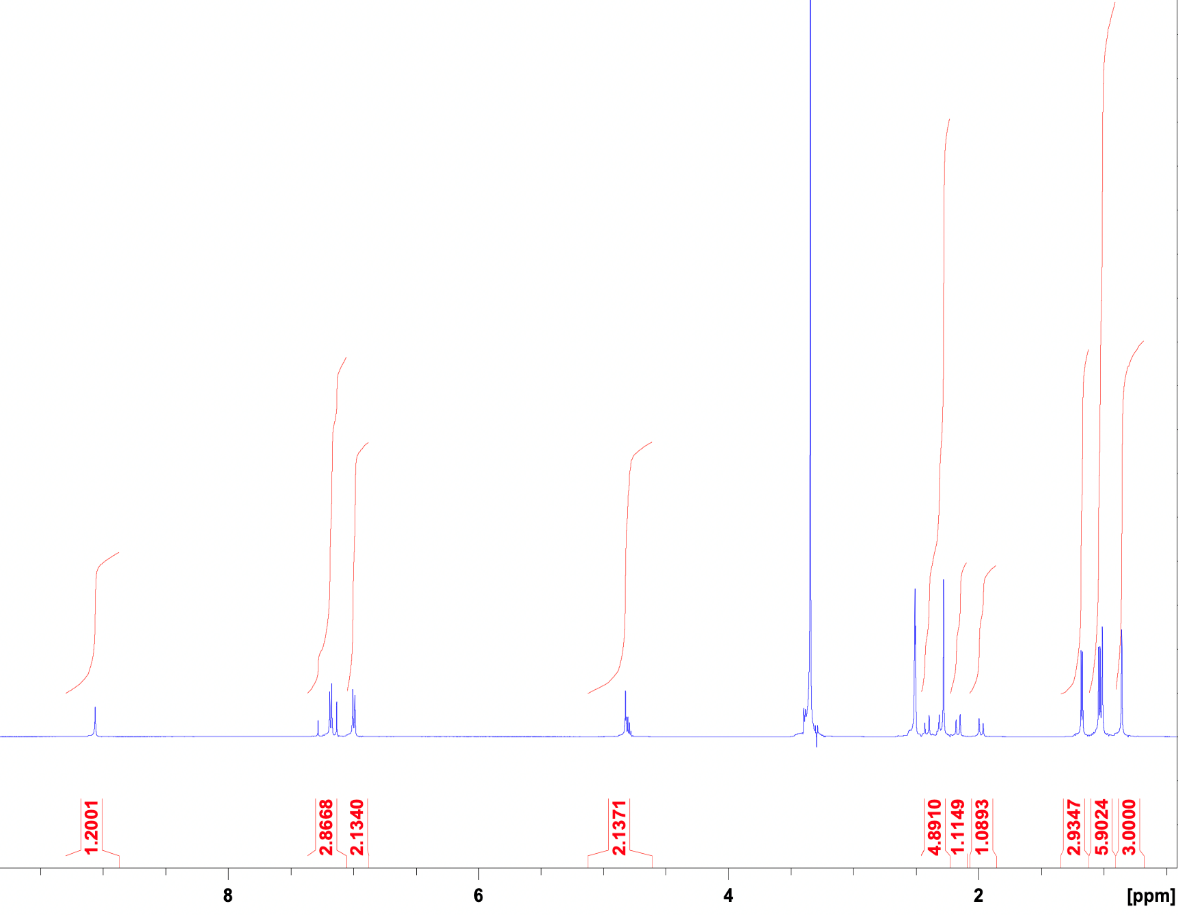

^13^C NMR spectrum of compound **3c**


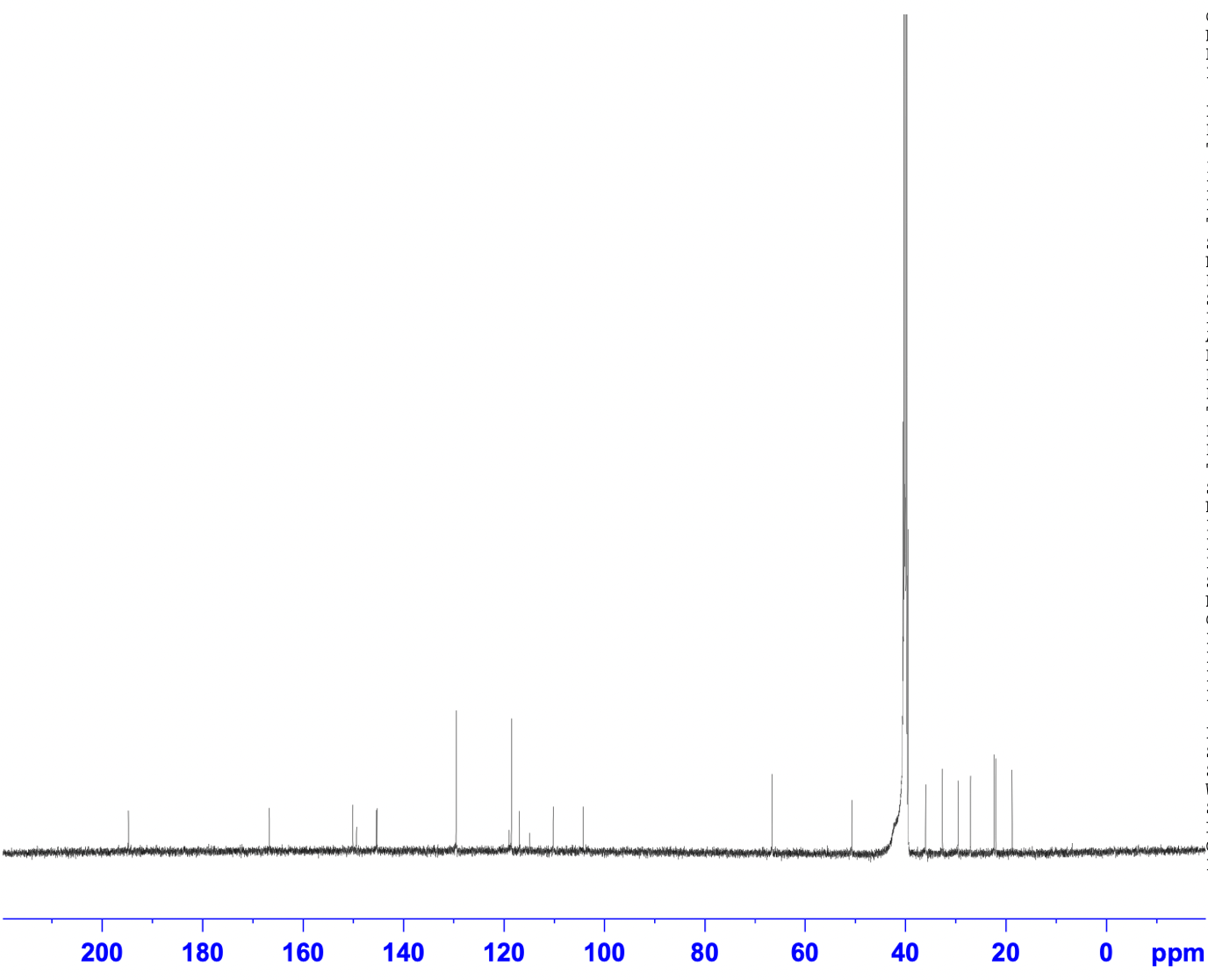

Mass-spectrum of compound **3c**


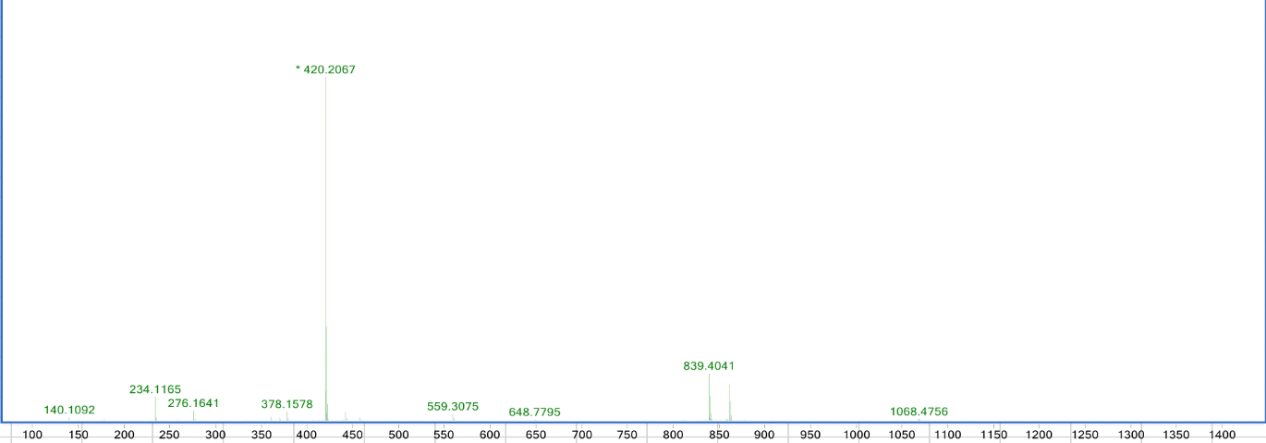


IR spectrum of compound **3d**


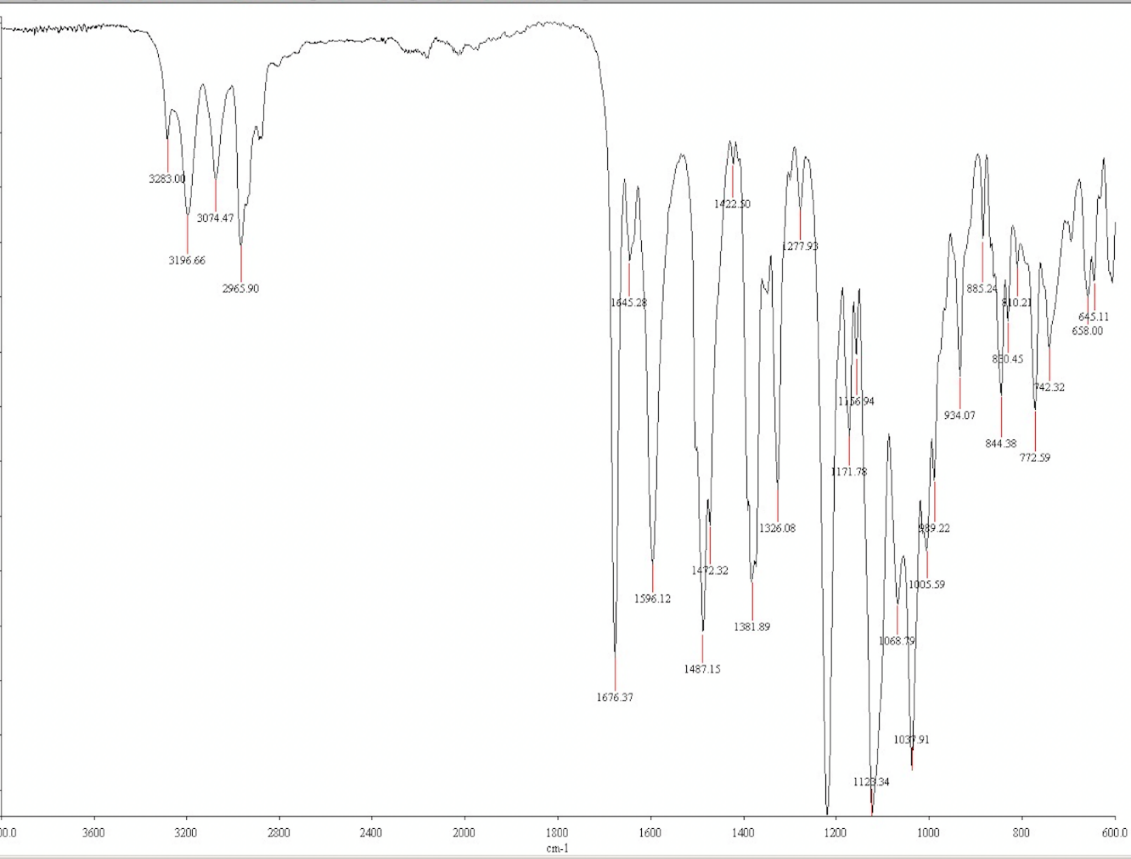


^1^H NMR spectrum of compound **3d**


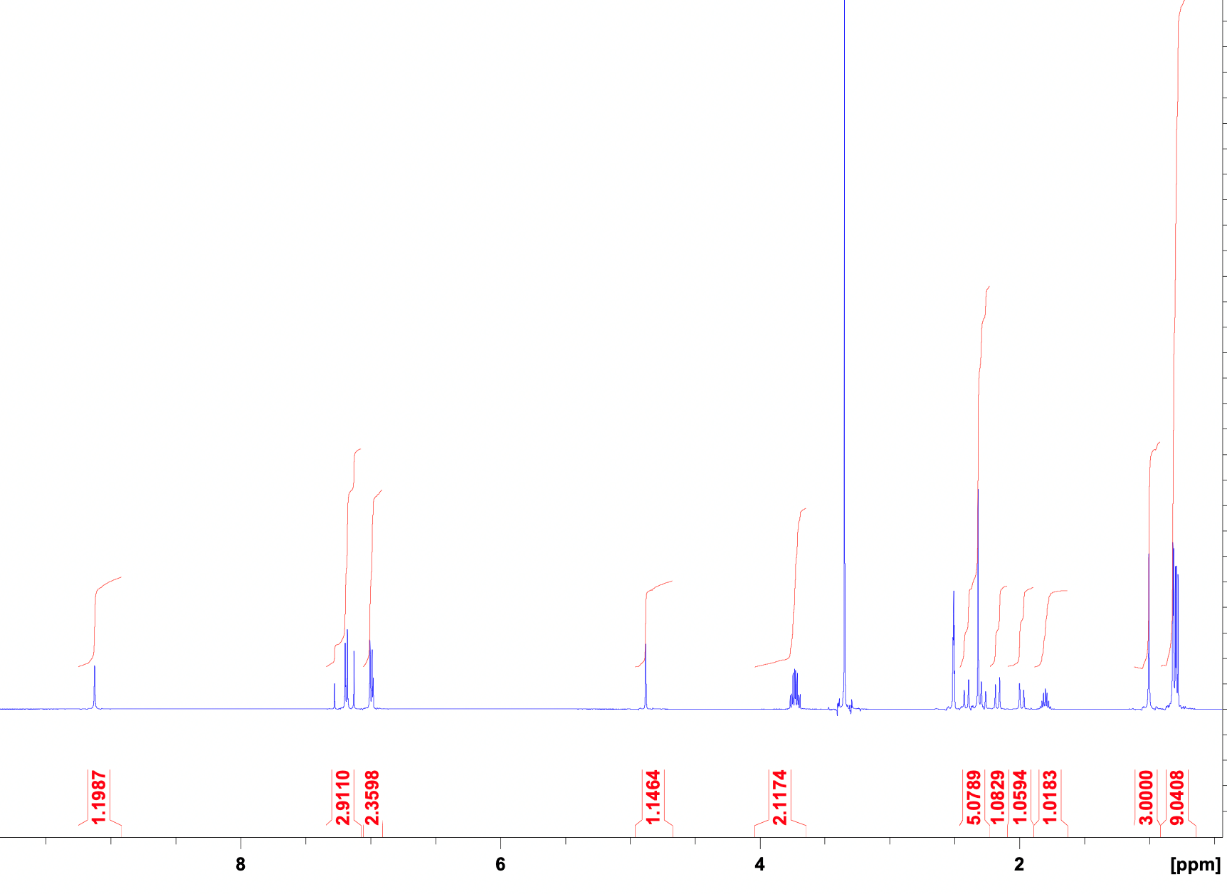

^13^C NMR spectrum of compound **3d**


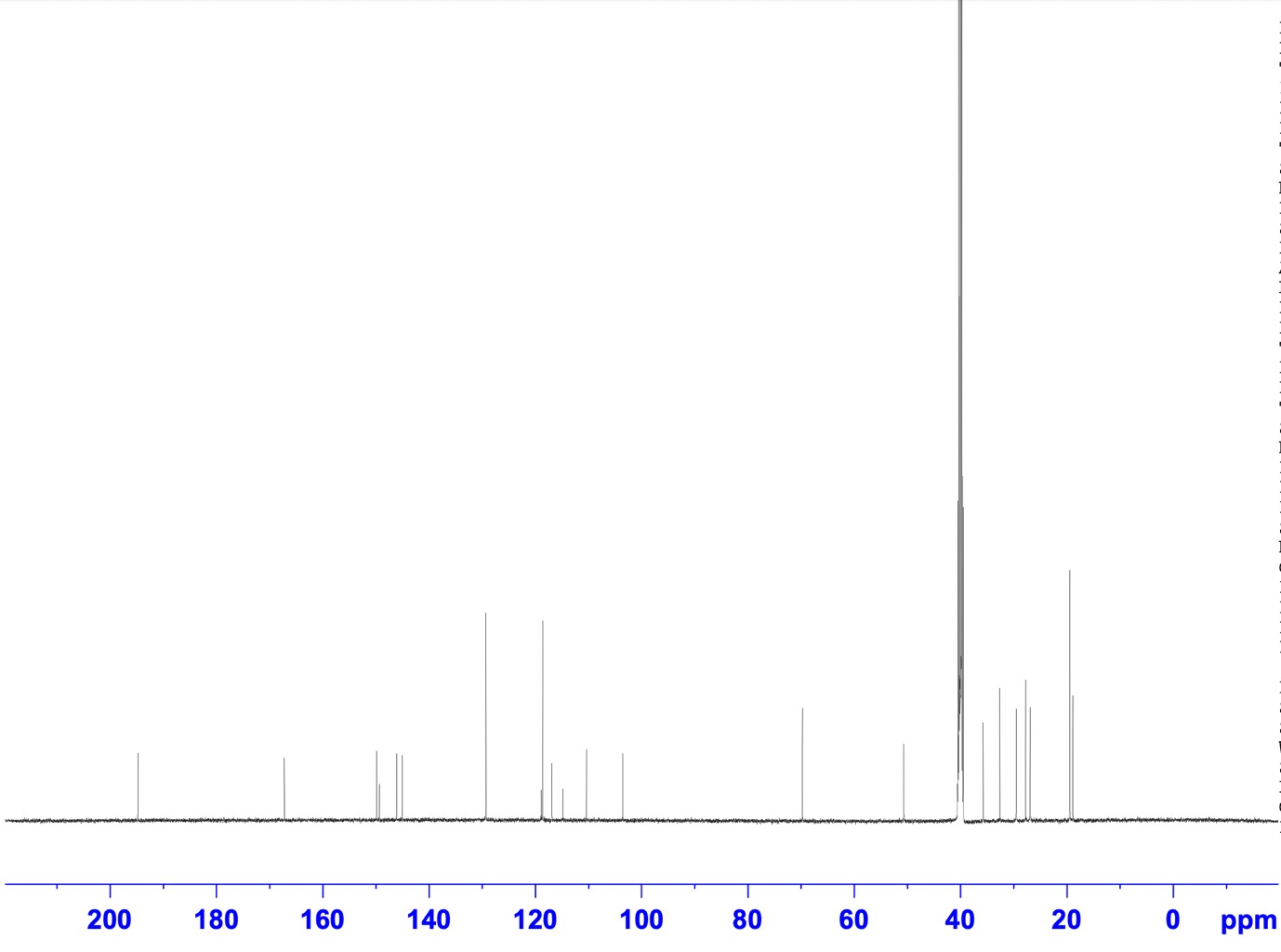

Mass-spectrum of compound **3d**


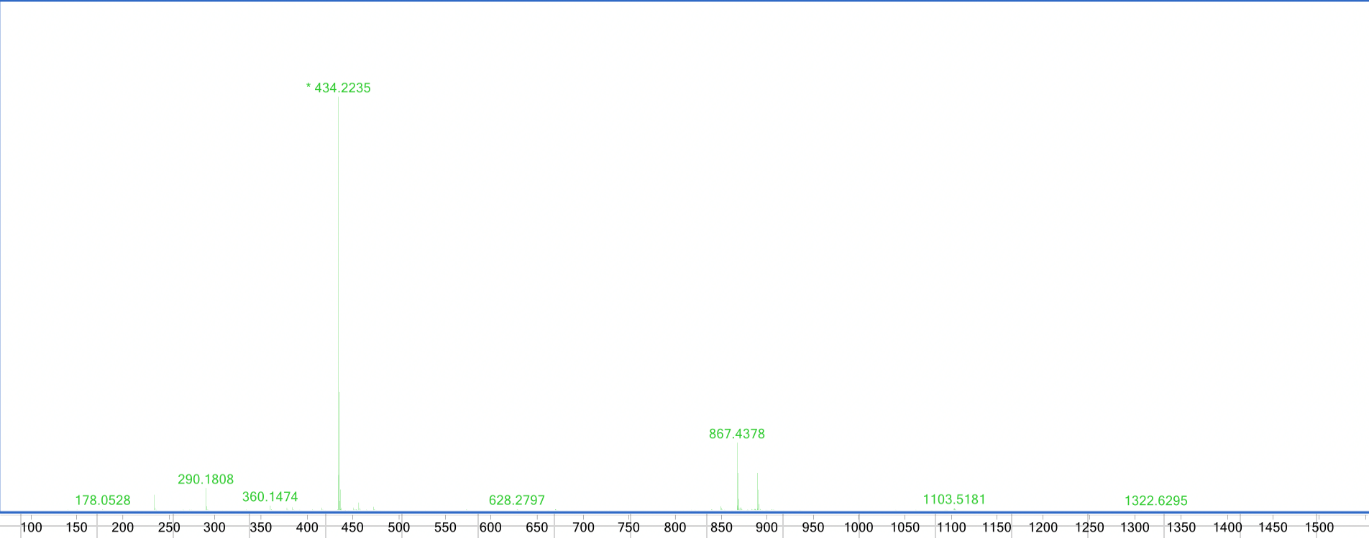


IR spectrum of compound **3e**


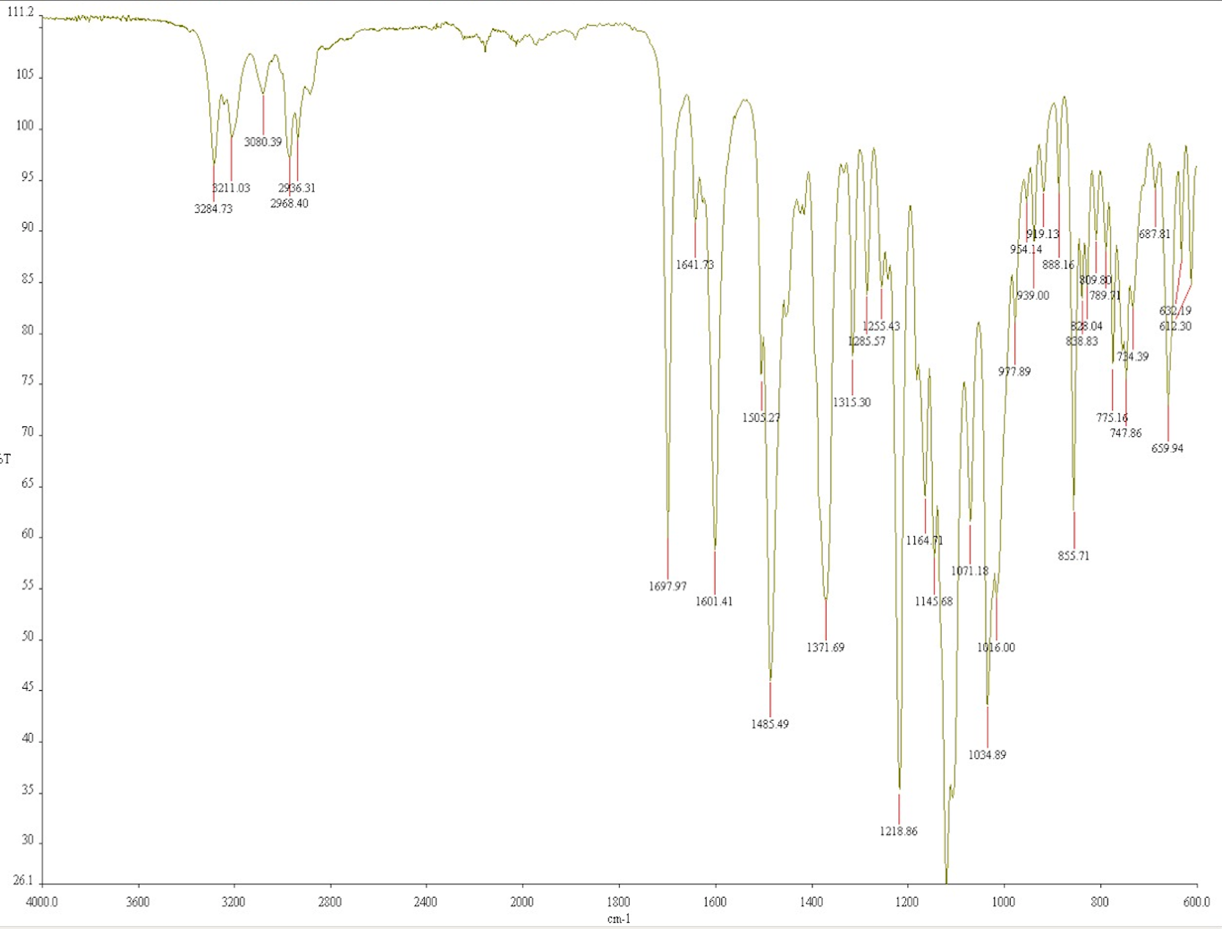


^1^H NMR spectrum of compound **3e**


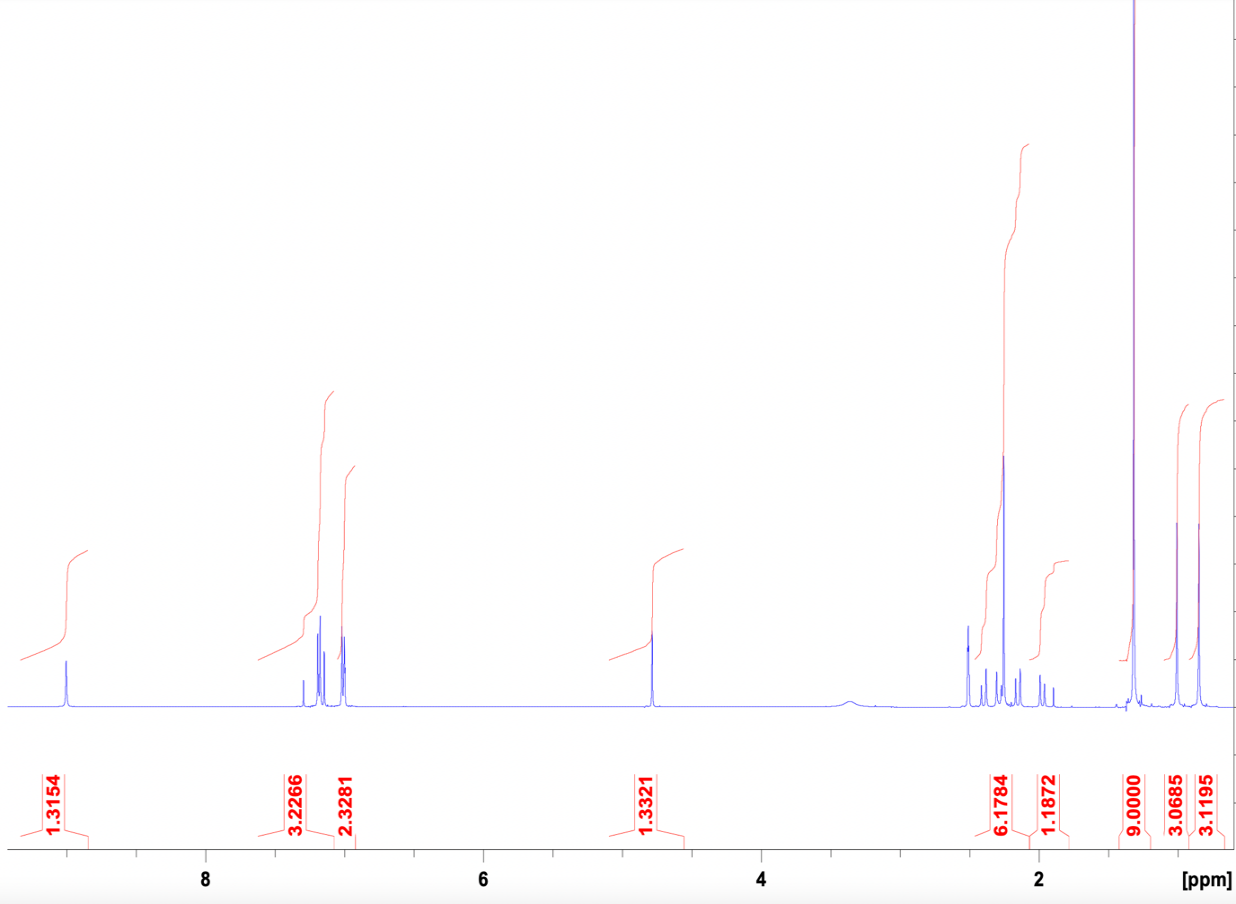

^13^C NMR spectrum of compound **3e**


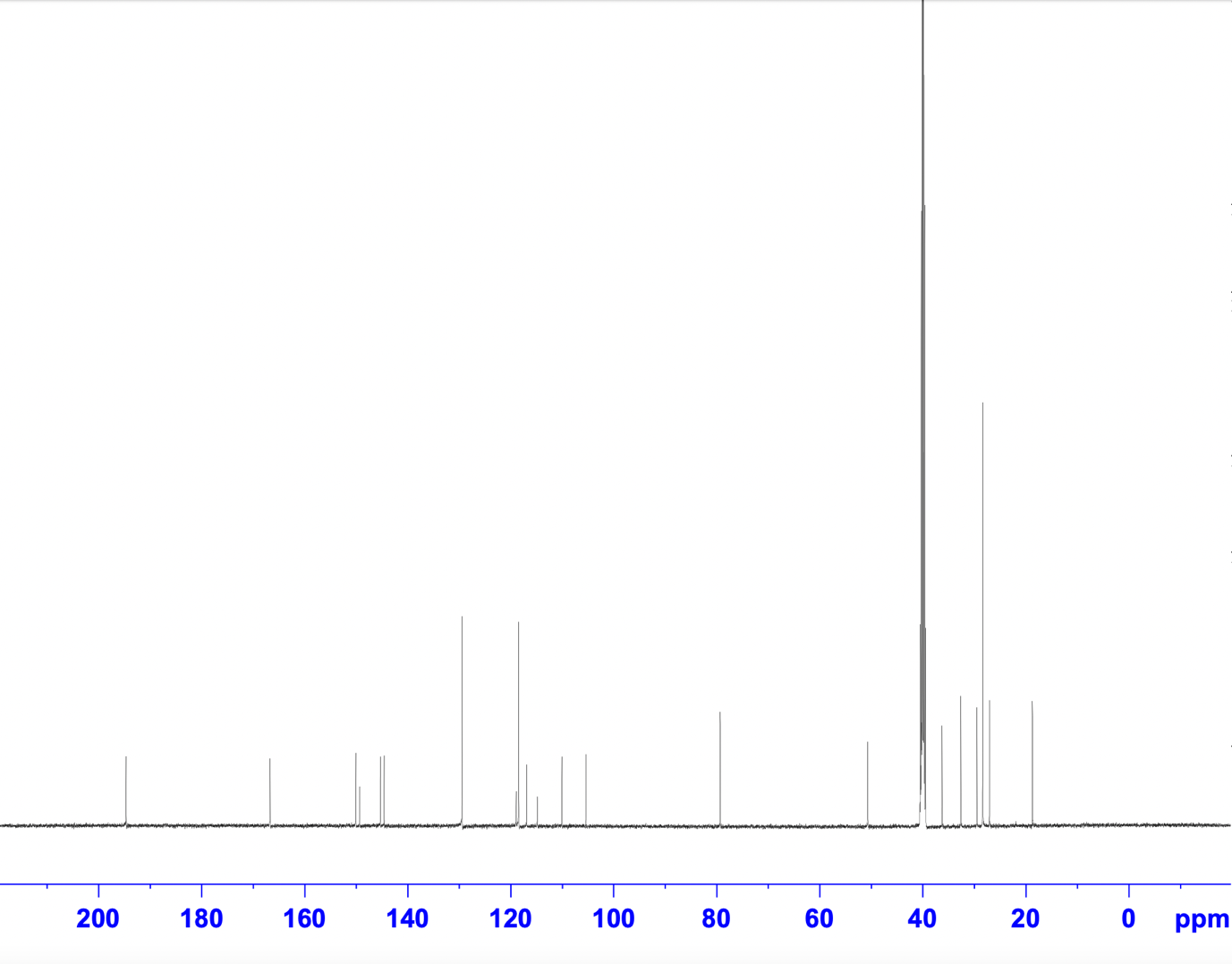

Mass-spectrum of compound **3e**


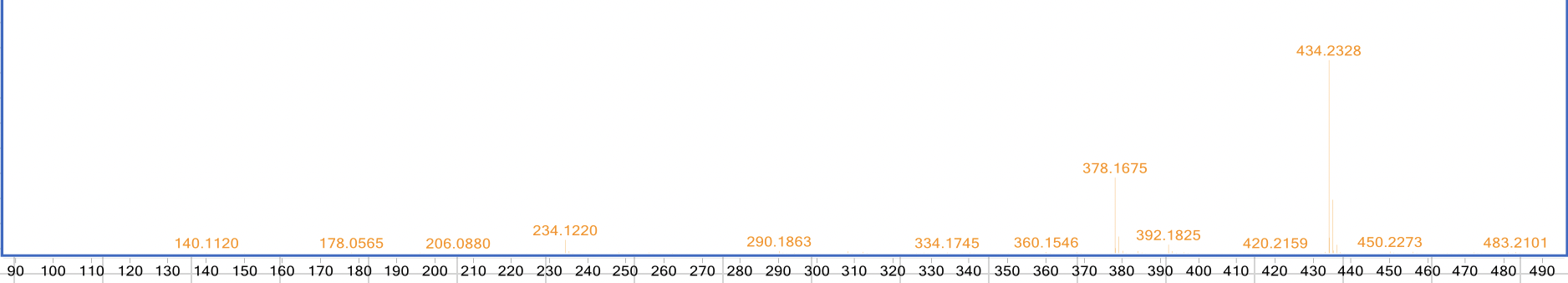

Supplement: Supplementary file 1 [file Supp_Inf.docx]
